# Paracrinicity: The Story of 30 Years of Cellular Pituitary Crosstalk

**DOI:** 10.1111/j.1365-2826.2007.01616.x

**Published:** 2008-01

**Authors:** C Denef

**Affiliations:** Laboratory of Cell Pharmacology, University of Leuven, Medical School Leuven, Belgium

**Keywords:** paracrine, autocrine, growth factors, peptides, neurotransmitters, cytokines

## Abstract

Living organisms represent, in essence, dynamic interactions of high complexity between membrane-separated compartments that cannot exist on their own, but reach behaviour in co-ordination. In multicellular organisms, there must be communication and co-ordination between individual cells and cell groups to achieve appropriate behaviour of the system. Depending on the mode of signal transportation and the target, intercellular communication is neuronal, hormonal, paracrine or juxtacrine. Cell signalling can also be self-targeting or autocrine. Although the notion of paracrine and autocrine signalling was already suggested more than 100 years ago, it is only during the last 30 years that these mechanisms have been characterised. In the anterior pituitary, paracrine communication and autocrine loops that operate during fetal and postnatal development in mammals and lower vertebrates have been shown in all hormonal cell types and in folliculo-stellate cells. More than 100 compounds have been identified that have, or may have, paracrine or autocrine actions. They include the neurotransmitters acetylcholine and γ-aminobutyric acid, peptides such as vasoactive intestinal peptide, galanin, endothelins, calcitonin, neuromedin B and melanocortins, growth factors of the epidermal growth factor, fibroblast growth factor, nerve growth factor and transforming growth factor-β families, cytokines, tissue factors such as annexin-1 and follistatin, hormones, nitric oxide, purines, retinoids and fatty acid derivatives. In addition, connective tissue cells, endothelial cells and vascular pericytes may influence paracrinicity by delivering growth factors, cytokines, heparan sulphate proteoglycans and proteases. Basement membranes may influence paracrine signalling through the binding of signalling molecules to heparan sulphate proteoglycans. Paracrine/autocrine actions are highly context-dependent. They are turned on/off when hormonal outputs need to be adapted to changing demands of the organism, such as during reproduction, stress, inflammation, starvation and circadian rhythms. Specificity and selectivity in autocrine/paracrine interactions may rely on microanatomical specialisations, functional compartmentalisation in receptor–ligand distribution and the non-equilibrium dynamics of the receptor–ligand interactions in the loops.

## Introduction

Paracrinicity is the process of short-distance communication between cells by way of substances released, shed or just ‘presented’ by a cell that affect a specific target on other cells in the neighbourhood. The substance reaches its target by diffusion in the extracellular space or by direct contact formation. Nowadays, it is recognised that paracrine communication is essential for body plan organisation and cell commitment during embryonic development, differentiation and proliferation of cells during postnatal growth and many functional activities of cells in the adult life of a multicellular organism. Paracrinicity is distinct from hormonal communication that occurs via the bloodstream and from neuronal communication that occurs in specialised synaptic structures. It is also distinct from communication between cells via gap junctions in the plasma membrane of neighbouring cells allowing passage of small messenger molecules (< 1000 Da) from the cytoplasm of one cell to that of another to co-ordinate the activity of groups of cells (1).

The present review looks into the history of how these concepts were born, how they were introduced in the field of neuroendocrinology and what the current picture of paracrinicity is in the context of the integrative functions of the pituitary gland. From early embryonic development to old age, the microenvironment surrounding the pituitary cells appears to consist of an extremely complex and ever growing number of players. We will focus mainly on the functional aspects of paracrinicity and, at times, implicate morphological aspects underlying these functions. More specialised aspects of morphogen and growth factor actions during development will only be reviewed when relevant for the later functions of the differentiated cells. The reader is referred to the excellent reviews in this field (2–6).

### From Cajal and Starling to Feyrter and beyond: walls segregating hormones, neurotransmitters and tissue factors broken down

The hypothesis of local humoral action can already be found in the classical paper of Bayliss and Starling, unequivocally demonstrating that gastrointestinal secretion is not only nerve-driven, as stated by the Pavlov doctrine, but also the result of the action of a hormone (secretin) produced in the duodenal mucosa and released in the blood in response to acid, stimulating exocrine secretion of the pancreas (7). At the end of the paper, a side observation is reported concerning the presence of a vasodilator substance different from secretin in the experimental duodenal extracts. In the summary section of the article, this reads as follows:

‘Acid extracts of the mucous membrane normally contain a body which causes a fall of blood-pressure. This body is not secretin, and the latter may be prepared free from the depressor substance by acting on desquamated epithelial cells with acid. There is some evidence of a specific localised action of the vasodilator substances which may be extracted from various tissues’.

Around the same period, Henri Dale had discovered histamine as a bioactive substance in mould ergot, which caused vasodilatation (8) and, in 1920, Popielski reported that histamine stimulates exocrine secretion from the stomach even after section of the vagal nerve. He suggested that the active principle with similar activity, found earlier in tissue extracts, might be histamine (9). The identity of this tissue factor as histamine was demonstrated in1927 by Best (10), but whether histamine was a hormone or a locally-acting factor remained unclear and clarification of its physiological significance in gastrointestinal function came only more than half a century later after James Black discovered histamine-2 receptor antagonists (11) and after the cell type producing histamine, the enterochromaffin-like cell, was identified in 1986 (12).

Another early suggestion for local regulation goes back to Ramon y Cajal at the end of the 19th century, who discovered a network of interstitial cells located between the myenteric nerve plexus and the smooth muscle layers and within these layers in the stomach and intestine (13). Cajal considered these cells as an interface system in the neurotransmission from the myenteric nerve perikarya to the muscle cells. Their stellate morphology and association in a network also indicated co-ordinating functions. For almost a century, the true nature of these cells as being neurones, Schwann cells, fibroblasts and macrophages has been debated. However, they are considered as specific cell types, sharing only certain staining characteristics with neurones. They have been shown to function as pacemaker cells in slow-wave peristaltic propulsion in the gastrointestinal tract. To this end, they use various chemical mediators and their activity is modulated by the innervating enteric nervous system.

In 1914, Masson (14) launched the idea that the solitary cells dispersed between the intestinal exocrine mucosa cells that fail to take up conventional stains are the enterochromaffin cells described earlier by Ciaccio. He suggested that they form a functional unit of hormone-secreting cells (15). In 1938, Friedrich Feyrter (16), an Austrian pathologist, described similar unstained cells (called in german ‘helle Zellen’ − clear cells) dispersed throughout pancreatic ducts and growing inwardly in clusters. Feyrter proposed that these cells form a functional unit of endocrine cells, in addition to the pancreatic islet cells (17). He included in that system the enterochromaffin cells of the intestine and the enterochromaffin-like cells of the stomach and called them ‘diffuse endocriene epitheliale Organe’. In 1953, he enriched the concept of the diffuse endocrine organs by also assigning the cells a paracrine function in his book, *Über die peripheren endokrinen (parakrinen) Drüsen des Menschen* (18). The merit of Feyrter has been that he considered dispersed endocrine cells as a novel type of organ, clearly distinguished from the classical concept of the body as being composed of compact organs (16), and broadened the sense of humoral communication over long distance by hormones to local communication by similar humoral factors acting locally. Feyrter also noticed the anatomical and functional relationship of the ‘helle Zellen’ with the submucosal part of the enteric nervous system, hereby uniting endocrine and neuronal regulation. Unfortunately, since the nature of the hormone-like substances that were postulated to act locally remained unknown, and since the relationship of them with secretory cells releasing true gastrointestinal hormones had not been illustrated by direct chemical identification, the notion of paracrinicity remained relatively silent for many years. A new impetus was given when Erspamer in 1952 identified serotonin in enterochromaffin cells (19).

In 1968, Pearse incorporated the endocrine/paracrine cells of the gut into a broader family of cells, called amine precursor uptake and decarboxylation (APUD) cells, on the basis of the neuronal characteristics that they displayed to manufacture monoamines by decarboxylating the precursor amino acid (20). In addition, they were found to produce biologically active peptides. The APUD cell family rapidly enlarged to some 40 members, including adrenal medulla and gut chromaffin cells, thyroid C cells, endocrine cells of the anterior pituitary, gut and pancreatic endocrine cells, carotid body chief cells, Merkel cells, melanocytes, endocrine cells of the placenta and thymus and sympathetic ganglia cells. However, since the role of the APUD cell monoamines has not been defined for each of the APUD cell types and some peptide-producing cells were found that lacked the typical APUD characteristic, the cell family was preferentially named the diffuse neuroendocrine system on the basis of expression of both hormonal and neuronal markers (15).

A parallel track towards the concept of local humoral control came from studies on carcinoid tumours in the gastrointestinal tract. The latter were first defined by Siegfried Oberndorfer (21, 22) in 1907 and proposed to be endocrine tumours by Gosset and Masson in 1914 (14). The endocrine nature of carcinoid tumours was suspected from the strong fibrosis that they produce in surrounding tissue and in remote areas, such as the heart, retroperitoneum and lungs. Feyrter had already identified a blood pressure increasing substance in extracts of carcinoid tumours in 1936 (23), whereas, in 1941, Selberg found blood pressure lowering material (24). In 1953, carcinoid tumours were shown to produce serotonin and, in 1964, Hallén suggested that the fibrosis associated with carcinoids (25) was probably due to local serotonin (26), although conclusive evidence still needs to be given today (27).

### Growth factors in tumours, salivary glands, serum and pituitary

Of great impetus in realising the importance of local humoral control has been the discovery of nerve growth factor (NGF) and epidermal growth factor (EGF) (28). Viktor Hamburger (29) paved the way to these discoveries by showing that peripheral tissues promote the growth of sensory and sympathetic neurones (30). In addition, Bueker demonstrated that implantation of a mouse sarcoma in the limb field of the chicken embryo results in an invasion of nerve endings from the embryo into the tumour area (31). These findings were reinvestigated by Levi-Montalcini who observed that the tumoural tissue stimulates the growth not only of closely positioned nerves, but also of many sympathetic and sensory neurones in the whole embryo. An impressive accidental observation speeded up the identification of the tumour factor. When treating a tumour extract with a phosphodiesterase preparation from snake venom to remove nucleic acids, Cohen and Levi-Montalcini (32) found that the snake venom preparation itself was contaminated by an impurity with the same biological activity as the tumour factor. Furthermore, the ‘activity’ of the snake venom contained amounts several thousand times greater than the tumour. This led to the isolation and purification by Cohen in 1959 (33) of what is now know as NGF and to the determination of its structure by Angeletti and Bradshaw in 1971 (34). A similar enrichment of NGF was found in extracts of mouse salivary glands. Further exploration of NGF-like material in the latter source unexpectedly led to the discovery of a substance that induced precocious eyelid opening and tooth eruption in neonatal mice (35). Cohen named the latter activity EGF and, together with Savage and Inagami, determined its amino acid sequence in 1972 (36). Cohen also characterised the EGF receptor and discovered its autophosphorylating tyrosine kinase activity, hereby opening an enormous new field of research. Interestingly, shortly thereafter, a factor first identified in 1936, urogastrone, which inhibits gastric acid secretion, was found to be similar to EGF (37–39).

A development initially not related to the concepts of local control, but later on was found to be of enormous importance, was the demonstration of factors in serum that are required for the growth, migration or survival of cells in culture. Since purification of these serum factors proved to be very difficult, some investigators tried to bypass this problem by testing pituitary hormone preparations for growth-promoting activity. In 1973 Armelin and Gospodarowicz were the first to identify a fibroblast growth factor (FGF) in the pituitary (40). This was subsequently purified by Gospodarowicz (41) and identified as a 14-kDa basic protein (basic FGF) (42). A 17-kDa acidic FGF was later isolated from the brain and shown to be highly angiogenic in the presence of heparin (43). This growth factor has since been recognised as one member of a large family of locally-acting factors essential for: (i) positional determination during embryonic development and (ii) controlling cell growth, repair, motility and survival during adult life. It also plays a prominent role in tumour development. Later, the pituitary also proved to be the source of another very important growth factor, vascular endothelial growth factor (VEGF) (44).

### Somatostatin and the big bang of paracrinicity

With the advent of techniques for high-speed peptide sequencing, peptide synthesis, immunoassay and immunocytochemistry in the late 1960s/early 1070s, knowledge of the chemical identification and tissue localisation of neuropeptides accelerated rapidly. It was soon realised that substances originally identified as hormones or neuromodulators also were putative paracrine factors. One of the first examples was somatostatin. This peptide was purified and identified by Guillemin's and Schally's group (45) in 1973 as the somatotrophin release-inhibiting factor released from the mediobasal hypothalamus into the portal blood vessels in the median eminence. Soon after, it was also identified by immunostaining in isolated and clustered cells of the gastrointestinal mucosa and pancreatic island d-cells (46). These cells showed peculiar morphological and topographical arrangements that were suggestive of a local control function on neighbouring cells. In the pancreatic islets, d-cells are mainly located between the β-cells, clustered in the middle of the islet and α-cells clustered in the periphery (47). In the stomach, mucosal d-cells send long cellular extensions along the nonluminal side of the mucosa (48).

From the 1980s, the research on local control reached an explosive phase. Several additional modes of local communication were discovered. In 1980, Sporn and Todaro introduced the concept of autocrine control, an autocrine factor being a substance released by the cell and affecting the cell of origin itself. They showed that cells that had been transformed by an oncogene in culture no longer required serum supplements because they themselves produced or overproduced the essential growth factors. The first growth factors identified on this basis were transforming growth factor (TGF)-α and TGF-β. This discovery opened an immense field of investigations on the role of autocrine growth factors in tumourigenesis and tumour progression. Several of these growth factors have been shown to be effective physiologically in the positional determination of cell fate, in expanding progenitor cell populations during embryonic development and in preserving cell diffentiation and survival in adult life (2–6). Many of these autocrine growth factors also influence cellular function and contribute to the pathogenesis of tumours (49).

Concepts of local control were also born in the field of Immunology. This year marks the 50th anniversary of the discoveries of interferon (IFN) (50). The subsequent finding that the supernatants of cultured lymphocytes contain soluble factors which enhance antibody production, led to the discovery of several factors affecting B cell growth and differentiation, called now interleukins (IL) (51). The first interleukin being cloned was IL-2 (52). IL-6, cloned 3 years later (53), was the first interleukin for which biological effects outside the immune system were described (51). These pleiotrophic factors were also shown to be local regulators of tissue cell turnover, the inflammatory response and tissue repair (54–56). Cytokines were also identified in specific cells in endocrine glands (57, 58) and neural tissue (59), as well as in hormone (57) target cells, and shown to modulate hormone or neurotransmitter action and help maintain tissue homeostasis and plasticity (60). Through the latter research the notion of a diffuse neuro-immuno-endocrine network in the body was born (59).

In parallel with these studies, a vast number of neuropeptides were discovered within the central and peripheral nervous systems and in the solitary cells in the diffuse neuroendocrine system (61). One of the first to be identified was Substance P in 1931 (62, 63). Substance P was described as a substance present in brain and gut that stimulated smooth muscle contraction in a way not blockable by atropine and which also lowered blood pressure. The peptide was sequenced by Susan Leeman in 1970. An enormous repertoire of neuropeptides which also act in non-neuronal tissues have since been discovered (64).

On the other hand, classical neurotransmitters, such as acetylcholine and noradrenaline, which were initially thought to be neurone-specific, were also discovered in discrete cells in several peripheral tissues (65–71). Serotonin, first identified in enterochromaffin cells, was later also found in many other tissues (72–75). In addition, purines (76), fatty acids and fatty acid derivatives (77), and nitric oxide (NO) (78) are synthesised and released locally to affect neighbouring cells. Importantly, the classical endocrine organs (ductless glands), pituitary, gonads, ovaries, the adrenal cortex and the thyroid gland, all contain cells which, in addition to hormones, produce certain of these messenger molecules which affect diverse functions within the gland. Today, more than 100 different bioactive substances have been identified in the anterior pituitary gland (Table 1) and most have been localised in specific cell types (Table 2). Considerable evidence suggests that these messengers effectively exert a local function during particular physiological changes. Thus, treatment with substances either blocking synthesis or release, or the action of these factors, results in obliteration of the changed functional response.

**Table 1:** Signalling molecules identified in the adenohypohysis.

Signalling molecules
Neurotransmitters
Acetylcholine
GABA
Purines
ATP, ADP
Arginine derivatives
Nitric oxide
Agmatine
Fatty acid derivatives
Prostanoids
Anandamide
2-arachidonoylglycerol
Retinoic acid
Neuropeptides
Vasoactive intestinal peptide
Galanin
Gastrin-releasing peptide
Neuromedin B
α-melanocyte-stimulating hormone
γ-melanocyte-stimulating hormone
β-endorphin
N-pro-opiomelanocortin
Enkephalins
Dynorphin
Nueropeptide Y
Substance P
Neurokinin B
Neuromedin U
Neurotensin
Vasopressin
Oxytocin
Delta-sleep-inducing peptide
Vascular growth factor peptides
Calcitonin
Calcitonin-R-stimulating peptide
Calcitonin gene-related peptide
Intermedin
Adrenomedullin, proadrenomedullin N-terminal 20 peptide
Atrial natriuretic peptide
B-type natriuretic peptide
C-type natriuretic peptide
Angiotensin II
Gastrin
Endothelins
Thyroid-releasing hormone
Gonadotrophin-releasing hormone
Growth hormone-releasing hormone
Somatostatin
Corticotrophin-releasing hormone
Urocortin
Urocortin II
Prolactin-releasing peptide
Ghrelin
Cocaine and amphetamine-regulated transcript
Orexin A
Orexin B
Neuropeptide B
Neuropeptide W
Apelin
26Rfa
Hormones and derivatives
Growth hormone
Prolactin
Cleaved prolactin
Glycoprotein hormone α-subunit
Growth factors
Fibroblast growth factor (FGF)-2, FGF-4, FGF-8
Epidermal growth factor, transforming growth factor-α, neuregulins,
Insulin growth factor (IGF)-I, IGFII
Nerve growth factor, glial cell line-derived neurotrophic factor
Transforming growth factor (TGF)-β1, TGF-β3, Activins, Inhibin,
Bone morphogenetic protein (BMP)-2, BMP-4
Pancreatitis-associated protein
Dll1, Dll3, Dlk1
Cytokines
Interleukin (IL)-1, IL-2, IL-6, IL-10, IL-11, IL-12
Leukaemia-inhibitory factor, interferon-γ, tumour necrosis factor-α, vascular endothelial growth factor,
Angiopoietins
Migration inhibitory protein, ciliary neurotropic factor, oncostatin M,
Leptin
Tissue factors
Insluin frowth factor-binding proteins
Follistatin, Noggin,
Interleukin-1 antagonist
Annexin 1
Adiponectin
Adiponutrin
Resistin

**Table 2:** Cellular Distribution of Signalling Molecules in the Anterior Pituitary.

	Cell types (L, lactotrophs; S, somatotrophs; G, gonadotrophs; T, thyrotrophs; C, corticotrophs; FS, folliculo-stellate cells)					
	
Signalling molecule	L	S	G	T	C	FS
Acetylcholine					▪	
GABA		▪				
Nitric oxide			▪			▪
Vasoactive intestinal peptide	▪					
Pituitary adenylate cyclase-activating peptide			▪			
Galanin	▪	▪		▪	▪	
Gastrin-releasing peptide	▪	▪			▪	
Neuromedin B				▪		
Neuromedin U					▪	
Corticotrophin-releasing hormone					▪	
Urocortin	▪	▪			▪	
Urocortin II					▪	
Neuropeptide Y	▪	▪	▪	▪	▪	
Atrial natriuretic peptide	▪		▪		▪	
C-type natriuretic peptide			▪		▪	
Neurotensin			▪	▪		
Dynorphin			▪			
Enkephalins		▪	▪	▪	▪	
Pro-opiomelanocortin			▪	▪	▪	
Angiotensin(ogen)			▪			
Calcitonin			▪			
Calcitonin gene-related peptide			▪			
Adrenomedullin, proadrenomedullin N-terminal 20 peptide			▪			
Intermedin					▪	
Substance P		▪		▪		
Endothelin-1	▪	▪				
Endothelin-3	▪		▪			
Vasopressin					▪	
Oxytocin	▪					
Ghrelin	▪	▪		▪		
Cocaine and amphetamine-regulated transcript	▪		▪		▪	
Orexin A	▪					
Orexin B					▪	
fibroblast growth factor-2			▪			▪
Transforming growth factor-α	▪	▪				
Transforming growth factor-β1	▪					▪
Transforming growth factor-β3	▪					
Activin			▪			
Inhibin			▪			
Leukaemia-inhibitory factor						▪
Migration inhibitory protein						▪
Interleukin-1				▪		
Interleukin-6						▪
Vascular endothelial growth factor	▪	▪	▪	▪	▪	▪
Angiopoietins			▪			
Leptin		▪	▪	▪	▪	▪
Follistatin			▪			▪
Annexin-1						▪

Another mode of local humoral control of cells is by so-called juxtacrine factors. This mode of control was discovered by Massagué who observed that the TGF-α precursor can be expressed as a plasma membrane-anchored polypeptide on the surface of cells and bind to EGF receptors on adjacent cells, in this way inducing both cell adhesion and cell division (79). Since then, many other examples of juxtacrine communication have been observed with mediators including several members of the EGF family, tumour necrosis factor (TNF)-α, colony-stimulating factor, platelet-activating factor and annexin-1. Juxtacrine communication provides a mechanism of strict spatial control of activation of one cell type by another, in contrast with paracrine control where the factor acts in the fluid phase within an action radius determined by its diffusion gradient. The active domain of a juxtacrine polypeptide can be cleaved from the cell surface by regulated proteolysis which will, if needed, abolish spatial specificity and which, in turn, will optimise diversity of communication but, by spreading, the signal could also be the start of disregulation.

Finally, some regulatory factors do not need first to be released by the cell and then activate a plasma membrane receptor, but they can also be active inside the cell of production after moving to another compartment and binding and activating receptors inside the cell. The latter mode of local control is called intracrine action (80).

## Early suggestions for local control in the anterior pituitary gland

Until 1970, the anterior pituitary gland did not attract attention in terms of local control systems. It was considered as a classical endocrine organ in which each hormone was produced by a specific cell type (one hormone–one cell type theory) with no obvious anatomical subdivision according to cell types except, in most mammalian species, for the intermediate lobe. Histochemical staining procedures were used to distinguish the different cell types (81). However, a proportion of the cells were considered hormone-free as they failed to be stained by these histochemical procedures and were named ‘chromophobes’. A peculiar group of cells that did not contain secretory granules was discovered by electron-microscopic examination by Rinehart and Farquhar in 1953 and called follicular cells (subsequently folliculo-stellate cells) (FS cells) (82). These were the first cells for which a local function was suspected, although the proposals for such function remained little defined. As FS cells engage in phagocytosis of hormonal cells, they were thought to have a local house-keeping role in removing dying cells and waste products. Moreover, FS cells show a stellate shape with long cytoplasmic extensions between the granulated hormonal cells and often associate among each other to form tiny follicles filled with fluid or colloid material. On these grounds, the cells were thought to have some role in local transport of material.

The path to experimental evidence for local control of and by the hormonal cells was found by two other endeavors. One was morphological, the other functional. The classical one cell type-one hormone theory stated that each pituitary hormone was produced by a specific cell type but the theory was based on histochemical procedures used to discriminate one cell type from another. Final proof could only be given when the hormone itself was identified in the cell. In 1970, Nakane (83) was the first to report that, indeed, growth hormone (GH), prolactin (PRL), adrenocorticotrophic hormone (ACTH) and thyroid-stimulating hormone (TSH) were stored in separate cell types but that many gonadotrophs showed immunoreactivity for both luteinising hormone (LH) and follicle-stimulating hormone (FSH). Another important morphological feature observed by Nakane (83) was that, although at first glance the different pituitary cell types were intermingled, they did not appear distributed homogeneously over different areas of the gland and within a particular cell cord. Nakane also observed close associations between somatotrophs and corticotrophs and between gonadotrophs and lactotrophs. Some of the PRL cells embraced the oval-shaped gonadotrophs with long cellular processes and were therefore named ‘cup-shaped’ PRL cells (83). Nakane suggested that the gonadotroph–lactotroph association might have functional consequences, although he only proposed that the cup exerted some kind of hindrance for secreted material to diffuse to the blood vessels.

A second path that led to exploring local control systems stemmed from efforts made to purify the cell types of the pituitary. The first success of enrichment of somatotrophs and lactotrophs was reported by Wess Hymer and was achieved by sedimentation of dispersed rat pituitary cells at unit gravity through a serum albumin gradient (84, 85). Large cells sediment faster than smaller cells and, as not all pituitary cell types are of the same average size, they enrich according to type. When the dispersed cells in the enriched populations were established in monolayer culture and compared with the less enriched ones, it became evident that their responses were functionally different (86). In 1976, we succeeded in highly enriching gonadotrophs by the latter technique (87). The success was owed to the choice of the animal model: instead of adult, 14-day-old female rat pituitaries were used, from the knowledge that female rat pituitary at that age contains more gonadotrophs and secretes more FSH than at any other time in life (88–90), and that, due to this high functional activity, gonadotrophs were expected to be hypertrophic or at least advanced in development compared to other cell types and would therefore sediment faster and, in this way, separate from other cell types. Indeed, the fastest-sedimenting cells were large gonadotrophs and they were enriched to a purity of approximately 75%. Most of these gonadotrophs contained FSH and LH. When we challenged these gonadotrophs with gonadotrophin-releasing hormone (GnRH) in culture, they released unexpectedly high amounts of FSH compared to adult animals (87). Another unexpected observation we made was that the magnitude of the FSH response to GnRH was different between these gonadotrophs and smaller gonadotrophs isolated in other fractions from the sedimentation gradient (91, 92). Also the FSH : LH ratio showed marked differences among fractions and these differences also were sex-dependent, males showing more heterogeneity than females (91, 92). These findings clearly suggested functional heterogeneity among the gonadotrophs, which raised the next question: what is the mechanism of functional heterogeneity? Do these differences represent differences between true gonadotroph subtypes or differences in hormone synthesis/secretion caused by alterations of the microenvironment due to sorting out certain cell types. We tested the latter hypothesis by comparing separated with recombined cell populations and found that recombination of the purified gonadotroph population with the gonadotroph-poor population again altered the FSH : LH ratio in the response to GnRH (93). These data suggested that signals from nongonadotroph cells contribute directly or indirectly to the mechanism of differential control of FSH and LH secretion. This observation was of particular importance since, up to now, all GnRHs stimulate both LH and FSH release, implying that a local mechanism in the pituitary must exist that is capable of controlling FSH independently of LH secretion. The cell separation and recombination experiments suggested to us that such a local mechanism might rely on intercellular communication with other pituitary cell types. Some years later, we showed that this gonadotroph-poor population consists mainly of PRL cells, some GH cell and nonhormonal cells, most of which are FS cells (94) and, as will be discussed below, all these cell types appear to be involved in cross-talk with gonadotrophs. FS cells are an important source of follistatin and follistatin attenuates the action of activin on FSHβ synthesis and secretion in a paracrine manner (95).

The next natural question was: do gonadotrophs also affect the function of PRL, GH and FS cells? Indeed, they appear to do so. When purified gonadotrophs were mixed with the PRL cell population, we found that GnRH unexpectedly evoked PRL release.

## Gonadotrophs signal to lactotrophs, somatotrophs and corticotrophs

### Gonadotrophs acutely affect PRL and GH secretion in early postnatal rat pituitary

In the 14-day-old rat model, we examined PRL secretion in the PRL cell population from which gonadotrophs had been removed by unit gravity sedimentation. GnRH had no effect on PRL release, as expected. However, when the 75% pure gonadotroph population was mixed with the PRL cell population in a 1 : 3 ratio and established in monolayer culture, at high cell density in order to optimise cell–cell contact, GnRH did evoke PRL release. These data were not published at that time as we had started to culture cells as three-dimensional aggregates, which were felt to be superior in terms of their tissue-like organisation (96). We were very lucky that this culture system was brought to our attention by Dr J. J. Cassiman, a colleague in our institute, who used it for studying cell adhesion mechanisms of fibroblasts. He and his PhD student, B. Vanderschueren, found that fibroblasts do not proliferate in an aggregate configuration and even are auto-digestive (97). Since pituitary cell monolayer cultures are usually over-grown with nonhormonal cells, which at that time were considered as fibroblasts, particularly when plated at low density and when culture medium was supplemented with serum, we felt the aggregate system was physiologically more reliable than the monolayer cultures. Moreover, aggregates can easily be used in a perifusion system, allowing the examination of rapid secretory responses as a function of time. In perifused aggregates, both the magnitude of secretory responses and the sensitivity to secretagogues were considerably greater than those of static incubations of monolayer cultures (98). With this technology and the background information from our previous studies on 14-day-old rat gonadotrophs, we clearly demonstrated the first evidence for communication between gonadotrophs and lactotrophs (99). When perifused pituitary cell aggregates from 14-day-old female rats were exposed to a GnRH pulse, an acute and dose-dependent stimulation of PRL release was seen from doses as low as 10 pm. The PRL response occurred in dopamine-free condition and also during coperifusion with 10 nm dopamine that on its own suppressed basal PRL secretion to more than 85%. No such response was seen in the gonadotroph-poor PRL cell-enriched aggregates. However, when a small percentage of a population consisting of 75% gonadotrophs was coaggregated with the lactotroph preparation, GnRH elicited a clear-cut acute stimulation of PRL release, the magnitude of which increased with the proportional number of gonadotrophs added to the lactotrophs. Thus, the GnRH effect on PRL release appeared to be mediated by gonadotrophs or required the presence of these cells in the vicinity of the lactotrophs. Also, aggregates from 14-day-old male rats showed this response. In aggregates from adult rats, however, no PRL response to GnRH could be elicited, at least not at day 4 in culture. When gonadotrophs from the 14-day-old rat were coaggregated with an enriched PRL cell population of adult males, GnRH did elicit PRL release, indicating that the PRL-releasing signal is produced only by immature gonadotrophs, but that lactotrophs remain responsive to it up to adult age. A curious phenomenon was that, when aggregates from adult rats were maintained for 3 weeks in culture, they acquired some PRL responsiveness to GnRH (99). In the coaggregates, thyroid-releasing hormone (TRH) stimulated PRL release, as expected, but did not affect LH or FSH release, indicating that the gonadotroph-lactotroph communication is not a random bidirectional communication. We also obtained evidence that the signal from gonadotrophs is a secreted molecule. A large number of purified gonadotrophs was incubated for 3 h in the absence and presence of GnRH and, subsequently, the spent media was perifused over the lactotroph population; we found that the spent medium obtained without GnRH stimulated PRL release, but that the spent medium, obtained in the presence of GnRH, and perifused together with an excess GnRH antagonist, induced a considerably higher PRL release.

We also looked at GH secretion in these 14-day-old rat aggregates and found an impressive GH response to GnRH as well. However, the secretory response was dual (100). During the GnRH pulse, GH release was rapidly inhibited, whereas, after stopping the application, a rapid rebound secretion of GH was observed that only slowly returned to initial basal release values. The total amount of GH released after the exposure to GnRH was considerably higher than that inhibited during GnRH exposure, suggesting that GnRH had both inhibitory and stimulatory actions on GH release. The latter conclusion was confirmed by the finding that pertussis toxin pretreatment turned the initial GnRH-induced inhibition of GH secretion into a stimulation.

The paracrine cross-talk with lactotrophs and somatotrophs was also confirmed by coaggregation of a lactotrophs/somatotroph population from 14-day-old rats with cells of the gonadotrophic cell line αT3-1 (101). It was found that GnRH induced a stimulation of PRL release and a dual effect on GH release, although the magnitude of the response was smaller than in normal pituitary. Furthermore, medium conditioned by αT3-1 cells contained PRL secretory and GH-inhibitory substance(s) (101).

We found out that, *in vivo*, the gonadotroph–lactotroph and gonadotroph–somatotroph secretory cross talk may be a process typical for the early postnatal period (102). No PRL or GH secretory response to GnRH was seen in freshly isolated intact pituitaries from day 1 (P1) rats; some response was seen on P3, rose in magnitude on P5 and decreased thereafter to be almost absent at the age of 14 days. As to GH secretion, some stimulation was seen at P1, followed by a rebound upon withdrawal of GnRH; both stimulation and rebound augmented in magnitude until P5 and became low again at P14. Apparently, establishing the 14-day-old rat pituitary cells in culture rejuvenates the cells to an earlier developmental phenotype. The significance of this observation remains unknown, but the findings inspired us to also investigate developmental actions of GnRH on lactotrophs and somatotrophs.

### Gonadotrophs manage lactotroph and somatotroph development

Independently, Bégeot *et al*. (103) reported that GnRH stimulates the development of lactotrophs in rat Rathke's pouch explants. Since the latter effect could be mimicked by treatment with the free glycoprotein hormone α-subunit (αGSU) and blocked by an LH antiserum cross-reactive with αGSU, the authors proposed that, during fetal development, GnRH exerts a paracrine action within the pituitary anlagen. GnRH is present in the rat embryonic pituitary as soon as embryonic day (E) 14 (104) and in amniotic fluid as soon as E12 (105) and the GnRH receptor is present in Rathke's pouch as early as E12 as well (104).

We have shown that GnRH mRNA is expressed in Rathke's pouch explants (106). Furthermore, treatment of Rathke's pouch explants with a GnRH antagonist depressed the development of lactotrophs, consistent with a paracrine action of GnRH in the explants. Importantly, the endogenous GnRH appeared only active (or present) provided some adjacent mesenchym was retained within the explant (106), suggesting a much broader paracrine system is operating during lactotroph development. Although GnRH is not essential for the development of gonadotrophs and lactotrophs, since these cells still develop in the hypogonadal mouse (that lacks GnRH) (107), it may have a trophic action at E11–E12, which is long before the terminal differentiation of these cells at E17–E18 (108, 109). The mechanism of that early action, however, remains unknown, but other factors seem to compensate the absence of GnRH, indicating the robustness of the lactotroph developmental track.

A gonadotroph-lactotroph axis is also supported by *in vivo* experiments in a transgenic mouse model (110, 111). By targeting the diphtheria toxin A gene selectively to the gonadotrophs with the bovine αGSU promoter fragment, the great majority of gonadotrophs is destroyed as examined in newborn mice (P1–3), at a time that oestrogens are not produced or are not active yet. The number and size of PRL cells and the size of the clusters they make, as well as PRL mRNA level, were significantly reduced in the DTA mouse pituitary compared to the wild-type mice, with no alterations seen in thyrotrophs and ACTH cells. However, there was no change seen in the number and size of GH cells or GH mRNA level. Other studies also revealed no changes in somatotroph number in adult transgenic mice with targeted ablation of gonadotrophs, nor did they find a change in late fetal life (112, 113). It is possible that the inhibitory action of gonadotrophs on GH cell proliferation is compensated by other factors or is not operational at P1–3 or that inhibition of somatotrophs is downstream of the stimulatory effect on lactotrophs. In that case, there would be barely an effect seen when lactotrophs are depressed.

A trophic action of GnRH on PRL production has also been noticed in the sheep fetal pituitary (114). Consistent with the trophic action of gonadotrophs on lactotrophs is that, in the hypogonadal gonadectomised mouse, treatment with GnRH slightly increases PRL synthesis even in adult life (115).

### Gonadotrophs act through mitogenic and recruitment signals

As far as tested in the 14-day-old rat pituitary aggregate cell culture system, treatment with GnRH for 40 h was found to enhance cell cycle entrance of lactotrophs and to lower that of somatotrophs (116). GnRH also inhibited the mitogenic effect of GH-releasing hormone (GHRH) on somatotrophs (117). GnRH also increased the total number of cells containing PRL mRNA within 40 h (118), suggesting that new lactotrophs are recruited by differentiation of a progenitor cell type into PRL-expressing cells, rather than to a mitogenic action on pre-existing lactotrophs alone. Conversely, the total number of cells expressing GH mRNA was significantly reduced by GnRH (117). Also, prolonged treatment of aggregates with GnRH was found to expand the population of cells expressing PRL mRNA as well as of cells expressing αGSU mRNA (119).

By means of cell separation and recombination experiments, we again showed that the developmental actions of GnRH depend on the presence of gonadotrophs (116). Mediation by paracrine factor(s) was indicated by the finding that medium conditioned by a highly enriched population of gonadotrophs cultured in the presence of GnRH, mimicked the effects of GnRH (120, 121). Also, medium conditioned by the gonadotroph precursor cell line αT3-1 contained material stimulating development of lactotrophs and inhibiting development of somatotrophs (101). Four substances with a selective mitogenic effect on lactotrophs were partially purified by high-performance liquid chromatography fractionation, whereas two other substances were isolated with antisomatotroph action (120, 121). These data clearly indicated that separate factors determine reciprocal development of lactotrophs and somatotrophs.

### Gonadotroph–lactotroph axis in adult life, a context-dependent system?

It has repeatedly been shown that peripheral injection of GnRH increases plasma PRL concentrations in adult rats (122–124), mice, hamsters (125), monkeys (126) and humans (127–133). Endogenous GnRH seems to exert a similar effect since treatment of castrated female monkeys with a GnRH antagonist causes PRL plasma concentrations to drop (126, 134) and hyperprolactinemia induced by oestrogen/progesterone treatment is attenuated by administration of a GnRH antagonist (135). Also in female rats, blockade of endogenous GnRH by injection of anti-GnRH antiserum causes hypoprolactinemia (136). The finding of a PRL response to GnRH is, however, not always consistent and may depend on hormonal status, sex, circadian rhythms and other physiological and pathological conditions. Some investigators found GnRH to increase plasma PRL concentrations in normal women during the follicular (127) and luteal phase (133) of the cycle and in postmenopausal women (131), whereas others found that the response was significantly greater in the luteal phase and in progesterone-treated menopausal women compared to women in early follicular phase (132). During the luteal phase, LH and PRL pulses are synchronous (133). Other studies only found a PRL response to GnRH in women during the periovulatory period and not during the late follicular or the midluteal phase (128–130). Pretreatment with testosterone 6 h prior to GnRH administration allowed such a response to occur in the follicular phase (137). A clear-cut rise in plasma PRL level was also seen in girls (average age 10 years) with Klinefelter syndrome (primary gonadal failure) (138). In the latter study, oestrogen treatment was found to decrease the PRL response to GnRH. Interestingly, the latter study is probably better comparable to the PRL response we have observed in immature rat pituitary since we found that addition of a low physiological dose of oestradiol (30 pm) in the culture medium facilitated the PRL response to GnRH but that a high dose attenuated it (Denef *et al*., unpublished observations). In men, a PRL response to GnRH was only seen after oestrogen treatment (139, 140). In untreated men, the PRL response to TRH was larger after prolonged GnRH pretreatment (141).

PRL responses are not always observed in normal people but can be seen under certain pathological conditions, such as anorexia nervosa and bulimia (142, 143), in hypergonadotrophic hypogonadal women (144, 145) and women with functional hypothalamic amenorrhea (145), in polycystic ovary syndrome (146) and in women treated with benzodiazepines (147). In human pituitary cell cultures, GnRH stimulated PRL secretion in an oestrogen-free environment and was inhibited by pretreating the cells with oestradiol (148).

It should be noticed that all these data only show that there is a PRL response to GnRH *in vivo* but it cannot be distinguished where these effects are established by a paracrine mode of action or by an indirect action at the median eminence or by a direct action at the level of the lactotrophs. Paracrine and direct actions of GnRH on lactotrophs are not mutually exclusive. It is known that GnRH is taken up in gonadotrophs, as expected, but also in lactotrophs, most likely by receptor-mediated endocytosis (149). Furthermore, it should be noticed that GnRH has been reported to stimulate PRL release from a clonal cell line derived from embryonic pituitary cells (150). Also in teleost fish, GnRH stimulates PRL release in culture and this must be a direct effect on PRL cells since the rostral pars distalis is a nearly homogeneous population of PRL cells without gonadotrophs (151).

Whatever the mechanism, it seems that the effect of GnRH on PRL release *in vivo* is complex due to the fact that it may be part of a communication network of which some components can compensate for each other and the behaviour of that system seems context-dependent. It may also be difficult to disrupt by interference with exogenous GnRH. Moreover, PRL responses to GnRH during fetal and immature life could be based on different mechanisms than that observed in adult life or in certain pathological states. Consistent with this line of thinking are the observations that a PRL response to GnRH is not elicited immediately following a bolus injection, but does occur after some time during an infusion (140). Injection of a single dose of anti-GnRH antiserum into female rats is followed immediately by blockade of LH and ovulation but the pre-ovulatory PRL peak is normal, but only after several days hypoprolactinemia is seen (136). Another important parameter that might explain some controversies in findings may be the interaction between the hormonal status and the day–night cycle, since GnRH failed to elicit a PRL response in women in the early follicular phase of their cycle when given in the day, but did so when given at night and even more so when given during sleep (152).

Several investigators have shown experimentally that gonadotrophs are important for a normal activity of lactotrophs during adult life, and the data confirm that the system may be context-dependent. Infusion with a high dose of a GnRH agonist is known to down-regulate gonadotroph secretion. Torres-Aleman *et al*. (153) showed that, during the first 30 min of such an infusion, LH release rises but that plasma levels return to normal level within the next 2 h and that, during this time, interval PRL plasma levels also decrease significantly. When pituitary tissue was tested *in vitro* at that time point, they also released significantly less PRL than the controls. Furthermore, long-term *in vivo* treatment with a GnRH agonist was also shown to reduce basal PRL secretion strongly when examined *in vitro*, but this was only observed in female rats and not in males (154). Agonist treatment also blocked the increase in serum PRL concentration induced by the dopamine receptor antagonist haloperidol as well as hyperprolactinemia obtained by transplantation of the pituitary under the kidney capsule (155). In perifused pituitary cells, basal LH and PRL release is pulsatile and administration of a GnRH agonist at more than two pulses per hour was reported to cause desensitisation of the LH response simultaneously with a disappearance of pulsatile PRL release (156).

### Identification of paracrine factors involved in lactotroph development

#### αGSU

The first candidate paracrine factor from gonadotrophs proposed to be involved in the development of lactotrophs was αGSU. In fetal rat pituitary explants, Bégeot showed that the stimulation of lactotroph development by GnRH could be mimicked by addition of αGSU to the explants and blocked by an LH antiserum cross-reactive with αGSU (103). We found large amounts of αGSU in conditioned medium from gonadotrophs and a semipurified fraction containing αGSU immunoreactivity stimulated PRL mRNA expression and lactotrophs mitogenesis, clearly suggesting that αGSU may be implicated in a paracrine gonadotroph network. Chronic (8 days) treatment with αGSU also stimulates PRL production in ovine fetal pituitary explants taken at gestational day 50 (114), only the acidic variant of the natural (free) αGSU and not the αGSU dissociated from LH showing this activity (157). These data are of substantial interest because, until now, a specific receptor for αGSU has not been identified. A stimulatory action of αGSU on PRL secretion has also been reported in frogs (158) where αGSU is coexpressed with PRL in and released from some lactotrophs (158, 159).

It should be realised, however, that αGSU may also be derived from other cell types. Recently, we showed that prolonged treatment of aggregates from 14-day-old rat pituitary with GnRH expands the population of cells expressing PRL mRNA as well as of cells expressing αGSU mRNA, but that a population of cells developed (approximately 20%) that expresses both PRL and αGSU mRNA (119).

#### N-pro-opiomelanocortin (POMC)

We have identified one of the trophic factors in gonadotroph-conditioned medium mediating lactotroph recruitment and PRL mRNA expression. This factor was purified from that medium and found to be the glycosylated N-terminal fragment of POMC, POMC1-74 (N-POMC) (120, 121). We also showed that N-glycosylation is essential for bioactivity and that certain glycosylated isoforms stimulate PRL mRNA level whereas others have a mitogenic action in lactotrophs (160). That a subpopulation of gonadotrophs is capable of synthesising N-POMC was shown at the mRNA level by means of single cell reverse transcription-polymerase chain reaction (RT-PCR) (161, 162), and some cells also show immunoreactive N-POMC and αGSU colocalisation (161). Nonglycosylated N-POMC, prepared by recombinant synthesis, keeps a specific mitogenic action on lactotrophs (and no other cell types) but its efficacy is weaker and, importantly, the effect is mediated via the γ3-melanocyte-stimulating hormone (MSH) moiety in its C-terminal domain by the melanocortin-3 receptor, whereas glycosylated N-POMC has no action through the melanocortin-3 receptor (163). Moreover, γ3-MSH is mitogenic for somatotrophs and thyrotrophs, in addition to lactotrophs (164). These data suggest that paracrinicity can be tuned to highly specific targets by differential post-translational modifications of the paracrine pro-peptide.

We also presented evidence that endogenous N-POMC is tonically involved in lactotroph mitotic activity and PRL gene expression, as treatment of pituitary aggregates with polyclonal or monoclonal antibodies raised against N-POMC significantly decreased the lactotroph mitotic rate (164). Other investigators also found N-POMC increases PRL and GH mRNA expression and stimulates PRL and GH secretion (after 12 h of treatment) in frog pituitary cell cultures (165). A crucial experiment still lacking is to determine whether N-POMC is a mediator of the action of GnRH on lactotrophs by investigating whether the GnRH effect can be blocked by treatment with an anti-N-POMC antiserum. In this respect, it should be realised that gonadotrophs may not necessarily use GnRH as their physiological agonist and that different agonists may elicit different paracrine contacts with lactotrophs. For example, neuropeptide Y (NPY) can mimic the action of GnRH on lactotroph mitosis in pituitary cell aggregates (116) but it inhibits the stimulation of PRL mRNA by GnRH (118).

The paracrine action of N-POMC in the pituitary has not yet been studied *in vivo*. However, some indirect data are available from mice in which the POMC gene was disrupted. These mice develop a profound phenotype, characterised by obesity, extreme adrenal hypoplasia (166), a more active hypothalamic-pituitary-thyroid (HPT) axis (167) and development of nonfunctional adenomas in the pituitary intermediate lobe (168). As to the status of the lactotrophs axis in these animals, no data are available yet. However, it has been reported that selective transgenic ablation of corticotrophs and melanotrophs in the pituitary (POMC neurones in the arcuate nucleus remain intact) does not result in a manifest change in the proportions of lactotrophs, somatotrophs and thyrotrophs (169), suggesting that paracrine actions of N-POMC are not essential and that other factors can compensate for its loss. However, the absence of a change in pituitary cell type distribution in the latter transgenic mice may also be explained by the fact that not all POMC cells are destroyed in these transgenic mice (170), and that the promoter fragment used to drive specific expression of the toxigene in classical POMC cells may not be the same as that used for activation of POMC expression in gonadotrophs, which would preserve POMC-expressing gonadotrophs from being destroyed.

#### Link with EGF-like molecules

A peculiar characteristic of the gonadotroph–lactotroph–somatotroph axis, which further supports the complexity and context-dependency of that system, is that the mitogenic effect of GnRH, as well as the mitogenic action of the substances in gonadotroph-conditioned medium, can be blocked by an inhibitor of the EGF-receptor (-R) tyrosine kinase (171). Since EGF or TGF-α are expressed in the pituitary of different species including human (172–175), more precisely in gonadotrophs (176), somatotrophs and lactotrophs (173, 174, 176), and since these growth factors are mitogenic for these cell types (171, 173, 175, 177, 178) and stimulate PRL gene expression and production (179, 180), these growth factors may have a permissive function in the paracrine action of gonadotrophs. This is supported by other findings. Treatment of cultures with antisense oligodeoxynucleotide to TGF-α (but not antisense to EGF) inhibited lactotroph cell proliferation induced by oestradiol (177). The mechanism may be direct or may rely on transactivation of an EGF-R present on lactotrophs and somatotrophs. It is well established that the EGF-R can be transactivated by agonists of numerous G protein-coupled receptors (GPCRs) (181). It is therefore plausible that various peptides from gonadotrophs act on the target PRL and GH cells via transactivation of the EGF-R expressed on lactotrophs and somatotrophs. Interestingly, expression of TGF-α is inhibited by TGF-β1 (182), which itself inhibits PRL gene expression and lactotroph proliferation, creating in this way a feedforward amplification of a negative control.

### Many other peptides in gonadotrophs may stimulate lactotroph function, but none have been shown to be involved yet

**Fig. 1:**
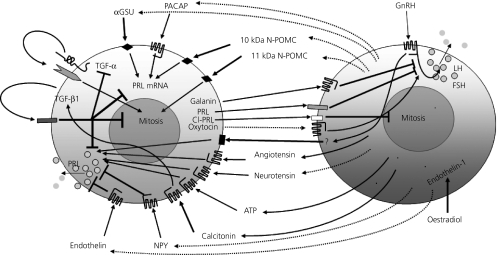
Schematic representation of the paracrine systems acting between gonadotrophs and lactotrophs. Full lines indicate pathways for which experimental criteria for paracrine action have been largely met. Interrupted lines are hypothetical interactions proposed on the basis of the presence of the indicated factors in the cell and their pharmacological effects on the other cell. →, Stimulatory effect; ⊥, inhibitory effect; Cl-PRL, cleaved prolactin; αGSU, glycoprotein hormone α-subunit; FSH, follicle-stimulating hormone; GnRH, gonadotophin-releasing hormone; LH, luteinising hormone; NPY, neuropeptide Y; PACAP, pituitary adenylate cyclase-activating peptide; POMC, pro-opiomelanocortin; PRL, prolactin; TGF, transforming growth factor.

An impressive number of peptides have been identified in gonadotrophs and, as they are secreted (183), they are potential candidates for a paracrine action on lactotrophs (Fig. 1), namely angiotensin II (184), neurotensin (185, 186), pituitary adenylate cyclase-activating peptide (PACAP) (187, 188), calcitonin (189), calcitonin gene-related peptide (CGRP) (190), atrial natriuretic peptide (ANP) (191), C-type natriuretic peptide (CNP) (192), proenkephalin A and B-derived peptides (193–195), cocaine and amphetamine-regulated transcript (CART) (196), NPY (197), endothelins (ET) (198, 199) and leptin (200, 201). TRH has been located in gonadotrophs maintained in culture, although this observation was not confirmed yet (202). Among these peptides, angiotensin II (184) and neurotensin (203–206) have well documented PRL-releasing activity in *in vitro* pituitary cell systems from adult rats. However, expression of neurotensin in gonadotrophs *in vivo* coincides with the prepubertal rise in plasma oestradiol throughout the second and third weeks in both sexes (185, 186) whereas, in the intact pituitary, the PRL response to GnRH is already decreasing by that time (see above). PACAP effects on PRL release are controversial and depend on the test system used (207–211). Whereas PACAP inhibits PRL release in monolayer cell cultures, it stimulates release in aggregate cell cultures and *in vivo* (207, 212). In studies where a stimulation of PRL release by PACAP was found in monolayer culture, the effect is probably on PRL gene expression and translation as it was only found after several hours of treatment (213). PACAP activates PRL gene expression (209, 214, 215) and is therefore a candidate peptide to participate in gonadotroph-mediated increase in PRL mRNA levels. ANP has no PRL-releasing action in mammals (216, 217) and whether CNP has such an effect seems unknown for mammals. In fish, ANP was reported to have PRL-releasing activity, although only after hours of exposure and not acutely as seen in our experiments (217). Leptin may be involved as it has been found to strongly stimulate PRL release (218). NPY is also reported to be stimulatory for basal PRL release by some investigators but others found it to inhibit basal and TRH-stimulated PRL release (219). CART has been reported to stimulate PRL release by some investigators but others found it to be inhibitory (196, 220). As to angiotensin II, a debate has going on for many years on whether or not there is an independent renin–angiotensin system expressed in the pituitary and in which cell types the different components are located (184). According to recent studies, there are two different renin-angiotensin systems (221): one is fully expressed within the gonadotrophs, with both renin and angiotensin II detectable in the regulated secretory pathway, but angiotensinogen appears to sort into the constitutive secretory pathway, raising a puzzling question how angiotensin II can then be formed within the regulated pathway. The second system seems to be extracellular with angiotensinogen located in perisinusoidal cells and angiotensin produced by circulating renin in the sinusoid lumen after release of angiotensinogen (221). Angiotensin II could then affect various other cell types downstream in the gland. Several investigators have reported PRL-releasing activity in response to GnRH in pituitary monolayer cultures, although only upon using a very high dose of GnRH (222). In reaggregate cell cultures kept in serum-free medium, we found that physiological doses of GnRH stimulate PRL release and, at these doses, neither an angiotensin-converting enzyme inhibitor, nor angiotensin receptor-1 antagonists were capable of inhibiting the GnRH-stimulated PRL release (222). Only at 100 nm GnRH could a partial inhibition of the PRL response by angiotensin receptor-1 antagonists be detected (222). Thus, angiotensin II may be involved in GnRH-stimulation of PRL release, but it seems to play only an accessory role at high concentration of GnRH. Possibly, the more physiologically relevant effect of GnRH is not involving the local renin–angiotensin system at all and gonadotroph-mediated stimulation of PRL release is mediated by another molecule or by a combination of substances.

A novel candidate may be PRL-releasing peptide (PrRP) (223) that does stimulate PRL release in aggregate cell cultures (224). In both intact pituitary and aggregates, we recently found PrRP mRNA and PrRP-like immunoreactivity, particularly in gonadotrophs associated with cup-shaped lactotrophs (Swinnen and Denef, unpublished observations). The peptide was also found in the culture medium by radioimmunoassay (Swinnen and Denef, unpublished observations).

### Gonadotrophs contain peptides that can affect somatotroph function

**Fig. 2:**
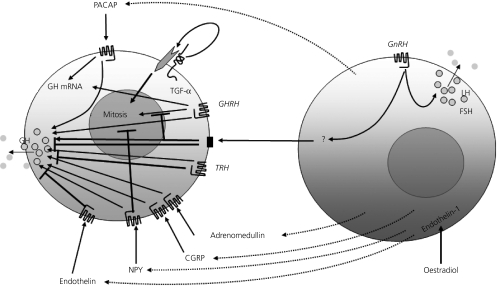
Schematic representation of the putative paracrine systems acting between gonadotrophs and somatotrophs. Full lines indicate pathways for which experimental criteria for paracrine action have been largely met. Interrupted lines are hypothetical interactions proposed on the basis of the presence of the indicated factors in the cell and their pharmacological effects on the other cell. →, Stimulatory effect; ⊥, inhibitory effect; CGRP, calcitonin gene-related peptide; FSH, follicle-stimulating hormone; GH, growth hormone; GHRH, growth hormone-releasing hormone; LH, luteinising hormone; NPY, neuropeptide Y; PACAP, pituitary adenylate cyclase-activating peptide; TGF, transforming growth factor; TRH, thyroid-releasing hormone.

Several peptides in gonadotrophs are also candidates for mediating the gonadotroph-induced effects on somatotrophs, although none of them have been shown to be implicated yet (Fig. 2). PACAP stimulates GH release and GH gene expression in several species (197, 207–211, 225, 226). CNP stimulates GH secretion in GH3 cells (227). ANP inhibits basal and GHRH-stimulated GH secretion in rat (228) but other studies have found it to be ineffective (216). We found that angiotensin II is a peptide displaying both inhibition and stimulation of GH release (229). However, angiotensin II antagonists were inactive in opposing the GH response to GnRH (100). Endothelins have a short-lasting stimulatory effect on GH release followed by a sustained inhibitory one (230). NPY stimulates basal and inhibits GHRH-stimulated GH release in swine pituitary *in vitro* (231). Also, TRH has dual effects on GH secretion, with a stimulatory component more pronounced in neonatal rats (232) and an inhibitory component prevailing at later age (233). TRH appears to have an inhibitory effect on GH gene expression (234). CGRP stimulates GH release (235). Another valid candidate is leptin as it has been shown to stimulate GH secretion and to inhibit GH cell proliferation (GH3 cells) (236). NPY stimulates basal GH secretion in rat pituitary cells loaded on a Bio-Gel P-2 column (237), in porcine monolayer cultures (238) and in gold fish pituitary fragments (239), but inhibits GHRH-stimulated GH release in porcine pituitary cultures (238).

### Calcitonin-like peptides, ET and NPY as inhibitory paracrine signals from gonadotrophs

Gonadotrophs have also been shown to interact with PRL cells in a negative fashion (Fig. 1), a phenomenon that makes sense in view that most stimulatory systems need an attenuating feedback mechanism for equilibrated functionality. Well-studied players here are calcitonin-like peptides and ETs.

#### Calcitonin

Calcitonin-like immunoreactive material has been detected, is synthesised and released by rat and chicken pituitary cells (240–242), is present in rat gonadotrophs (189) and in an αGSU expressing cell line (243), and the pituitary also expresses the calcitonin receptor (244). Treatment of pituitary cell cultures with calcitonin inhibits basal and TRH-stimulated PRL release (245, 246), lactotroph mitosis and PRL mRNA expression (247), but not TRH-induced TSH release or GnRH-induced LH release (245). Conversely, treatment of the cultures with an anticalcitonin antibody enhances PRL mRNA expression and PRL release (241), as well as lactotroph mitosis (247), suggesting that endogenous calcitonin is tonically active in a paracrine manner. Calcitonin has a similar antimitogenic action *in vivo* and passive immunisation with anticalcitonin antibody increases the mitotic index in lactotrophs (248) as well as serum PRL levels (249). Targeted overexpression of calcitonin in gonadotrophs of mice leads to long-term hypoprolactinemia, decreased PRL gene expression, female subfertility and a selective underdevelopment of lactotrophs (250). A calcitonin-like substance is also expressed in the chicken pituitary, although there it colocalises with PRL, but, as in the rat, its level fluctuates inversely with PRL mRNA level (251). In both rat and chicken, oestradiol is a negative regulator of calcitonin mRNA level (251, 252). In the rat, progesterone is a positive regulator. Most interestingly, calcitonin immunoreactivity is found mainly in gonadotrophs that are associated with cup-shaped lactotrophs (253), a clear-cut morphological characteristic supporting a paracrine role. Rat calcitonin produced in the anterior pituitary was shown to have the same amino acid sequence as calcitonin from the thyroid C cells, but, recently, another peptide, named calcitonin receptor-stimulating peptide, has been discovered that shows sequence homology with CGRP and has also been found to be expressed in the pituitary (254).

The paracrine action of calcitonin appears to be indirect, and at least in part, mediated by endogenous TGF-β1. TGF-β1 is expressed in lactotrophs (255, 256), its expression is enhanced by calcitonin and TGF-β1 in turn inhibits lactotrophs mitosis (256), PRL mRNA expression and secretion (257, 258). TGF-β1 acts as a paracrine and not as an autocrine factor on lactotrophs. In single cell experiments where PRL expression was followed in ‘real-time’ by quantification of photons emitted by the living cells by means of a luciferase reporter (injected in individual cells) under the control of the PRL promoter (259), PRL gene expression decreased upon exposure to TGF-β1 and treatment of the cells with a TGF-β1 antibody increased PRL gene expression. The latter, however, was only seen in lactotrophs that were adjacent to another lactotroph, suggesting that TGF-β1 acts in a paracrine and not in an autocrine manner. However, these experiments cannot exclude that TGF-β1 is acting in an autocrine manner and needs the context with other lactotrophs (i.e. that an intimate contact between lactotrophs is a prerequisite for the response to autocrine TGF-β1). We have found several examples of secretory responses that needed close association between cells, such as the GH response to angiotensin II (229) and the inhibitory PRL response to acetylcholine (260). Again, these observations emphasise the importance of context in paracrine and autocrine regulation.

#### Endothelins

Another inhibitory paracrine signal from gonadotrophs to lactotrophs may be mediated by ETs. ET1 and 3 are present in the mammalian anterior pituitary (261–263), more precisely in gonadotrophs of humans (264) and rats (199), although it as also expressed in subpopulations of somatotrophs and lactotrophs (199) and in gonadotrophs of frogs, particularly in female frogs (198). Treatment with ET1 or ET3 in culture profoundly inhibits basal PRL release (265–267), but, initially, there is a short-lived stimulatory action as well (268) or higher doses may be stimulatory (267). It remains to be shown whether the endogenous peptides exert such an action. As will be discussed below, ETs make an inhibitory autocrine loop in lactotrophs, which is well documented.

#### NPY

NPY has been found in gonadotrophs but whether it acts in an autocrine or paracrine fashion is not experimentally illustrated yet. Nevertheless, NPY has been reported to inhibit basal and TRH-stimulated PRL release and to be additive with dopamine in inhibiting PRL release (219). NPY also blocks the action of GnRH on PRL gene expression and mitosis and on GHRH-stimulated mitosis of GH cells in aggregate cell cultures.

### Gonadotrophs may positively and negatively interact with corticotrophs

**Fig. 3:**
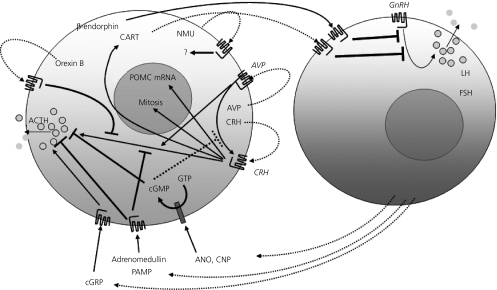
Schematic representation of paracrine systems acting between gonadotrophs and corticotrophs. Full lines indicate pathways for which experimental criteria for paracrine action have been largely met. Interrupted lines are hypothetical interactions proposed on the basis of the presence of the indicated factors in the cell and their pharmacological effects on the other cell. →, Stimulatory effect; ⊥, inhibitory effect; ACTH, adrenocorticotrophic hormone; ANP, atrial natriuretic peptide; AVP, arginine-vasopressin; CART, cocaine and amphetamine-regulated transcript; CGRP, calcitonin gene-related peptide; CNP, C-type natriuretic peptide; CRH, corticotrophin-releasing hormone; FSH, follicle-stimulating hormone; LH, luteinising hormone; NMU, neuromedin U; POMC, pro-opiomelanocortin.

Several peptides located in gonadotrophs modulate corticotroph activity when added exogenously to pituitary cell preparations and it is striking that most of these peptides are involved in volume homeostasis in other parts of the body. Among them are the calcitonin-like peptides adrenomedullin and CGRP and the natriuretic peptides (NP) ANP (191) and CNP (192, 269–272) (Fig. 3). The importance of these peptides is also emphasised by their similar distribution in gonadotrophs in other species, even nonmammalian (273) and by the observation that, during a 50% reduction in maternal adrenomedullin, gene expression in mice lacking one allele of the gene leads to profound defects in implantation, placentation and fetal growth (274).

#### NP

The NPs ANP, brain natriuretic peptide (BNP) and CNP have been shown to inhibit corticotrophin-releasing hormone (CRH)-stimulated ACTH release from mouse pituitary fragments (275) and isolated E21 fetal pituitary (276). Moreover, these peptides also reduced POMC mRNA levels (276). Receptors for NPs (NPR-A and NPR-B), both membrane guanylyl cyclases that generate cGMP as second messenger, have also been identified at the mRNA level in the anterior pituitary (277). That the effect is of physiological significance *in vivo* has also been shown (278).

#### Adrenomedullin (AM)

AM, a 52 amino-acid peptide, and proadrenomedullin N-terminal 20 peptide (PAMP), are peptides derived from the same precursor with important roles in the physiologic regulation of volume and electrolyte homeostasis (279). The peptides belong to the calcitonin peptide family together with CGRP, calcitonin, and amylin (280). In cell culture AM and PAMP inhibit ACTH secretion (269) and blunt CRH- and oxytocin-stimulated ACTH secretion (281–284). Intravenous administration of AM in sheep also lowered the plasma level of ACTH (285).

#### CGRP

A positive paracrine regulator of corticotrophs released from gonadotrophs may be CGRP as this peptide stimulates basal and CRH-stimulated ACTH secretion (286). These observations are interesting as intracerebroventricular administration of CGRP activates the hypothalamic-pituitary-adrenal (HPA) axis as well (287). It remains to be seen whether all these peptides act similarly as their pharmacology predicts and under which conditions they are important.

## Gonadotrophs as paracrine targets

There is substantial evidence for paracrine communication in the direction of the gonadotrophs. Cell separation experiments have shown a positive influence of nongonadotrophs on gonadotroph FSHβ expression (288) and the secretory FSH : LH ratio (93). The best characterised signalling systems in rats are from lactotrophs to gonadotrophs through galanin and from corticotrophs to gonadotrophs by opioid peptides (Figs 1–3).

### Galanin

This oestrogen-dependent peptide is found mainly in lactotrophs in female rats (289) and exposure of pituitary monolayer cell cultures or pituitary tissue fragments to galanin results in an acute inhibition of GnRH-stimulated LH and FSH release. Conversely, treatment with galanin antiserum augments the LH and FSH response to GnRH (290). Although these data support a paracrine inhibitory role in the rat, galanin was found to stimulate LH secretion in the porcine pituitary, whereas the use of antigalanin antiserum weakened GnRH-stimulated LH release (291). This may suggest an opposite regulation in porcine pituitary but the data were obscured by the finding that an anti-NPY antiserum had the same weakening effect on GnRH- as well as galanin-stimulated LH release (291).

### β-endorphin

It was found that β-endorphin inhibits basal as well as GnRH-stimulated LH release in pituitary cell culture (292, 293). This apparently also occurs in the tissue *in situ*, as treatment of cultured pituitary cells with naltrexone, an opioid receptor antagonist, or with β-endorphin antibodies, increased basal LH release (292). Moreover, CRH depresses basal LH release by cultured pituitary cells, and this was also blocked by naltrexone (292).

### Cart

CART is expressed in the pituitary (294) and it is striking to find the peptide in several cell types that can exert paracrine inhibitory feedbacks on GnRH-stimulated LH release. It is found in gonadotrophs (196, 295), lactotrophs (295, 296) and corticotrophs (297). CART release is increased by GnRH and TRH (295) and production is up-regulated by CRH (297). CRH is known to be a negative regulator of gonadotrophin secretion. Pituitary CART expression and secretion is up-regulated during lactation and down-regulated by dopamine, and fluctuates up and down during the oestrous cycle (lowest at dioestrous) (295), suggesting a broad role for the peptide in reproduction (296). Also leptin increases CART release (196). These regulatory findings are consistent with the localisation of the peptide in the different cell types. CART inhibits K^+^- (295) and GnRH-stimulated (220) but not basal (295) LH release in culture. CART also inhibits PRL release (196). These findings point towards a significant paracrine inhibitory action of CART on gonadotrophs, which can occur during different physiological changes via different cell types or in an autocrine manner when released from gonadotrophs. However, the paracrinicity potential remains still to be confirmed by immunoneutralisation studies and by examining release from identified cells.

### PRL and cleaved PRL

Lactotrophs may also signal to gonadotrophs through PRL and certain cleaved forms of PRL. Hyperpolactinemia is well known to inhibit LH secretion and to depress the hypothalamic GnRH pulse generator (298). The PRL receptor is already expressed in the rat anterior pituitary during fetal life (299) and has been found in subpopulations of different hormonal cell types, including gonadotrophs in rats (300) and sheep (301) but not in the horse (302). PRL has been shown not to tonically inhibit GnRH-stimulated LH release in rat monolayer cell culture (303). However, either intravenous administration of PRL or raising plasma PRL levels by immobilisation stress, reduced the LH response to GnRH pulses and GnRH-stimulated increase in GnRH-R density, whereas addition of antiserum against PRL lowered the responsiveness of LH cells to GnRH, consistent with a paracrine inhibitory tone of PRL on LH secretion (304). In sheep, PRL on its own was found to inhibit (305) or not affect (306) GnRH-induced LH release in culture but, in the presence of a dopamine agonist, it completely blocked it, at least when cells are taken in the nonbreeding season (306). Dopamine agonists were also reported to improve the LH response to GnRH when no exogenous PRL is present (305), which led the authors to speculate that this was the consequence of inhibiting endogenous PRL secretion. Whether immunoneutralisation of PRL also results in opposite effects needs to be studied.

We have reported that the large disulphide loop of PRL can be cleaved between Tyr145 and Leu146 with preservation of the disulphide bridges (307). The compound was isolated from spent culture medium of pituitary cell aggregates from 14-day-old rat pituitary and represented 0.6–1% of total bulk PRL. This cleaved PRL stimulates mitotic activity of LH and TSH cells but of no other cell types in rat pituitary aggregates (307), whereas treatment with polyclonal antibodies, raised against the new N- or C-terminals of cleaved PRL and not recognising native PRL, inhibits mitosis in these cells (308). Although these experiments show a paracrine tone by cleaved PRL on gonadotrophs, the physiological significance remains unknown. The compound is stereoselective as neither PRL, nor another somewhat larger cleaved PRL showed the latter bioactivity. Interestingly, in a recent study it has been shown that a 16-kDa cleaved PRL may be responsible for the postpartum cardiomyopathy syndrome (309), suggesting that cleaved PRL variants can be pathophysiological mediators in certain conditions of enhanced PRL output.

### Oxytocin

Another peptide synthesised within the anterior pituitary of which receptors are found in gonadotrophs is oxytocin (310). The cell type expressing oxytocin appears to be the lactotroph and not any other cell type (311). Oxytocin has been shown to stimulate basal LH release in culture as well as to potentiate GnRH-stimulated LH release (312), an effect only seen when pituitaries were tested at pro-oestrous (313). The peptide did not have to be present for the latter potentiation as it also occurred when oxytocin was administered to the perifusion for 2 h prior to GnRH (314). Thus, a paracrine function of oxytocin on LH release seems possible but remains to be experimentally demonstrated.

## Close association between cup-shaped lactotrophs and gonadotrophs in mammals and frogs and between gonadotrophs and somatotrophs in fish

Paracrine interactions with gonadotrophs may be rendered selective by microanatomical associations between cell types. Association between certain gonadotrophs and lactotrophs (often cup-shaped) and between certain somatotrophs and corticotrophs was already reported more than three decades ago in the rat pituitary by Nakane (83). These observations were confirmed by Siperstein and Miller (315) and Yoshimura and Nogami (316, 317). The lactotroph–gonadotroph associations were also reported in horse (302, 318), sheep (301), chicken (319) and frog pituitary (304). Associations between lactotrophs and gonadotrophs reconstitute in monolayer cultures of densely plated cells (320) and in reaggregate pituitary cell cultures (321), indicating that the affinity between cell types is locally regulated. In aggregate cultures, oestradiol significantly decreases the occurrence of cup-shaped lactotrophs embracing gonadotrophs (321). A functional correlate of the morphological association is the observation that the cup-shaped lactotrophs are always associated with gonadotrophs that contain calcitonin immunoreactivity and that the changes in PRL output *in vivo* evoked by ovariectomy, pregnancy and lactation in rats were opposite in direction to that of the PRL change (253), which is consistent with the inhibitory paracrine action of gonadotroph calcitonin on PRL gene expression (see above).

Another peculiar characteristic of anterior pituitary gonadotroph and lactotroph distribution is that these cells are densely represented near the intermediate lobe in several species such as rats and horse (83, 318, 322). In this way, these cells are well exposed to substances made by the neurointermediate lobe, such as the PRL-releasing factor intermedin (see below) and, at the same time, receive modulatory paracrine signals from the adjacent gonadotrophs. The higher incidence of gonadotrophs near the intermediate lobe has also been observed in the monkey pituitary and this is even a selective location in juvenile monkeys (323).

In teleost fish, gonadotrophs distribute in close association with somatotrophs. Unlike mammals, fish pituitary shows a zonation according to cell types (324). Lactotrophs are located in the rostral pars distalis, whereas somatotrophs reside in the proximal pars distalis, but there they distribute together with the gonadotrophs. Most interestingly, gonadotrophs always show a distribution as central cell clusters surrounded by a matrix of somatotrophs. This cellular association may form a microanatomical basis of local functional interactions and many data are consistent with this view. In salmonids, the population size of the somatotrophs fluctuates in parallel with that of the gonadotrophs expressing the gonadotrophic hormone GTH-II (LH in fish) during sexual maturation (325). In goldfish, the rise in GH level during sexual recrudescence and spawning and during the pre-ovulatory GTH-II surge always occurs together with that of GTH-II (326). GH has an important permissive role in reproductive functions in fish (327). Moreover, in fish, GnRH stimulates both GH and GTH-II secretion (328–330). Recently, direct evidence for paracrine interaction between the cell types has been reported. In experiments on carp pituitary cells, the effect of exogenous GTH and GH and of immunoneutralisations of endogenous GH or GTH indicated that GTH released from gonadotrophs stimulates GH release and synthesis in somatotrophs, whereas secreted GH maintains somatotroph sensitive to stimulation by GTH, and inhibits basal GTH release from gonadotrophs (331).

## Possible physiological significance of gonadotroph paracrinicity

Gonadotrophs play the central executive role in the orchestration of reproduction. However, these cells do not work alone. Homeostasis and adaptation of the pituitary to reproductive needs also requires adaptation to metabolic needs by a co-ordinated action between the hypothalamic-pituitary-gonadal (HPG) axis, GH and PRL and the HPT axis. Both PRL and GH have essential roles to play in reproduction and growth (332–335). A proper co-ordination of gonadotrophin, GH and PRL release by the pituitary and the relative representation of the respective hormone-producing cell types are therefore mandatory. This is already the case during development as shown by the accelerated lactotroph development from the second week after birth in the rat (336) that coincides with the rapid expansion of gonadotrophs (88–90), particularly in the females. Somatotrophs, by contrast, are already well developed at birth, and are well proliferating already during the first week of life (336). It therefore makes sense that gonadotrophs stimulate PRL cells during the second week after birth in the rat but that they release an inhibitory paracrine signal to somatotrophs. Since high FSH levels during the second postnatal week are thought to be an important stimulus for ovarian follicle development, and since ovarian maturation also requires GH, it is conceivable that GH output is also under a certain trophic control by gonadotrophs as well, hence, a dual effect of gonadotrophs on somatotrophs is not surprising. Attenuation of postnatal somatotroph expansion is also seen at the level of GHRH-R expression. GHRH-R mRNA is highly expressed just before birth and declines during the perinatal period to reach a nadir at 12 days of age and increases again at 30 days of age (337).

On the other hand, it is well known that stress-induced activation of the HPA axis inhibits the HPG axis, such as during stress and undernutrition, during which conditions for reproduction are not optimal, reproduction is even contraindicated and saving energy is important (338). At the pituitary level, the negative interrelation of the HPA and HPG axis may be mediated by the paracrine negative signals of β-endorphin on GnRH-induced LH release. In addition, CART, being located in several cell types involved in the stress response, can exert paracrine inhibitory feedbacks on GnRH-stimulated LH release.

On the other hand, in a different context, it may be mandatory that the reproductive system attenuates the stress response. Gonadotrophs may contribute to this goal by releasing NPs that in turn attenuate CRH-activated ACTH release. NPs inhibit the HPA axis at a hypothalamic level (339), which makes sense in view of the sodium and water retention properties of glucocorticoids during enhanced HPA axis activity. Stress responses need to be attenuated in certain physiological states, such as lactation and in situations that lead to low levels of visceral adipose tissue (340). It has been shown that, in such cases, responsiveness of the pituitary to hypothalamic CRH/vasopressin appears depressed (340). The cellular mechanisms still need to be explored, but one hypothesis is that the pituitary NPs located in gonadotrophs are involved through their paracrine actions, together with mechanisms located at the level of CRH production in the hypothalamus (340). The NPs are functional opponents of the renin–angiotensin system that is a positive component of the stress response in the control of fluid volume regulation at both central and peripheral level. A question in this respect is whether NPs are up-regulated by oestrogen in gonadotrophs. In heart tissue oestrogens and progesterone are known to up-regulate NP expression (341). An increased inhibitory tone by NP on corticotroph function during increased oestrogen exposure during pregnancy is therefore not unlikely in the pituitary as well.

Another contextual paracrine regulation is the inhibition of ovulation during lactation. The primary factor responsible is at the level of the GnRH pulse generator in the hypothalamus, where suckling-induced increase of endorphinergic input leads to inhibition of the GnRH pulses (298). The local lactotroph–gonadotroph association in the pituitary may contribute to this suckling-induced negative influence on ovulation via an inhibitory input of β-endorphin from corticotrophs. Since the HPA axis is activated during pregnancy and energy normally consumed for ovulation is senseless during pregnancy, the raised pituitary β-endorphin and galanin tone may contribute to silencing of the pre-ovulatory LH release. In addition, pregnancy may antagonise the HPG axis via the growing activity of the lactotrophs that make higher amounts of galanin under the influence of oestrogen, which in turn not only will increase lactotroph activity, but also weaken LH release at the same time in response to GnRH. As will be discussed below, another important local negative regulator of the HPG axis activated during lactation is the NO system in gonadotrophs and FS cells.

Although we have not been able to show a primary role of angiotensin II from gonadotrophs in paracrine regulation of GH and PRL release under basal conditions, such a role could exist in other contexts. For example, we found that angiotensin II is much more effective in releasing GH in pituitary aggregate cell cultures from hypertensive and prehypertensive spontaneously hypertensive rats (SHR) than in cultures from normal littermates (342) and, recently, it has been reported that, whereas normal rat anterior pituitary expresses the angiotensin AT1B-R, the SHR down-regulates AT1B-R and induces expression of the AT1A-R (343). It would be worthwhile to explore whether paracrine angiotensin II acts primarily through the AT1A-R. The SHR is also much more responsive to angiotensin II in terms of ACTH secretion (343). Since there is no evidence for delivery of angiotensin II into the hypophyseal portal blood (344, 345), it seems plausible that either circulating angiotensin II and/or the local renin–angiotensin system in the anterior pituitary is involved in the exaggerated stress response of the pituitary gland under pathological conditions but that, during pregnancy, this system is used to generate a normal increase in HPA axis activity. In addition, CGRP and adrenomedullin in gonadotrophs may help in modulating the HPA output during pregnancy as these peptides stimulate and inhibit, respectively, CRH-induced ACTH secretion at the level of the pituitary.

During recent years, considerable information was obtained in support of a cross-talk between body energy reserves and fertility (346–348). Metabolic demands increase substantially during pregnancy and lactation. Body weight and appetite increase. The mechanisms involved in the regulation of these homeostatic changes are still largely unknown (349). No data are available to associate one of the above discussed peptides with that homeostatic system. However, several orexigenic peptides that are involved in the up-regulation of the energy balance and feeding in the hypothalamus have also been detected in the anterior pituitary, such as ghrelin and orexins and both stimulate GH release (350). Orexins also enhance LH (351) and ACTH (352) secretion. Orexin-A is mostly found in lactotrophs and a small subpopulation of somatotrophs, gonadotrophs and thyrotrophs and orexin-B only in corticotrophs (350). Orexin-1-R is found in GH cells and orexin-2-R in ACTH cells (353). Thus, orexins are located to enable cross-talk at the pituitary level for achieving appropriate adaptations in energy homeostasis during pregnancy, but this area remains to be fully explored.

## Autocrine regulation of lactotrophs

An autocrine regulatory system is a stimulus–response system in which the cell releasing the stimulus is also the target. Various autocrine systems have been claimed in the anterior pituitary on the basis of the following criteria: (i) the cell produces and secretes the stimulus; (ii) the same cell type expresses the receptor for the stimulus; (iii) the cell type under study responds to the exogenously added stimulus; and (iv) addition of a receptor blocker or of an antibody immunoneutralising the endogenous ligand or its receptor has effects opposite to that of the exogenous ligand. Strictly, it should also be demonstrated that single cells plated on a large distance from other cells meet the same criteria. However, under the latter conditions, the context in which the autocrine system operates may be altered or even be destroyed and this may jeopardise the functioning of the system. Evaluation of autocrine loops in single cells has often been performed by means of the reverse haemolytic plaque assay. In this assay, PRL secretion is measured from individual cells that cannot interact by direct contact between each other and paracrine action is avoided if, at least, the distance between the cells is kept substantial (354).

**Fig. 4:**
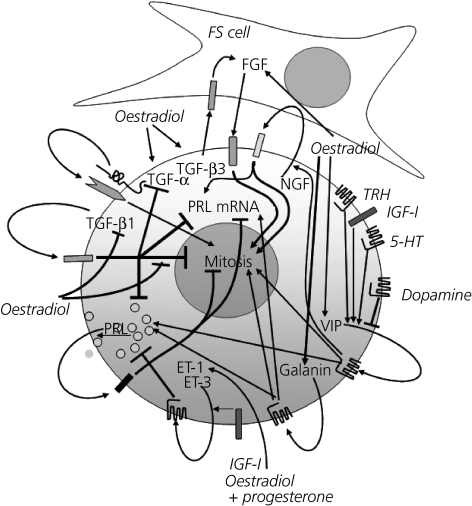
Schematic representation of the autocrine loops acting in lactotrophs. Full lines indicate pathways for which experimental criteria for autocrine action have been largely met. Interrupted lines are hypothetical interactions proposed on the basis of the presence of the indicated factors in the lactotroph and their pharmacological effects on the same cell. →, Stimulatory effect; ⊥, inhibitory effect; ET, endothelin; FGF, fibroblast growth factor; IGF, insulin-like growth factor; NGF, nerve growth factor; PRL, prolactin; TGF, transforming growth factor; VIP, vasoactive intestinal peptide.

There is firm experimental evidence for autocrine control of lactotroph secretion, PRL gene expression and growth of the lactotrophs cell population in terms of the first four criteria, but demonstration that the signalling cell is also the responding cell is only rarely provided. Therefore, autocrine systems can not always be distinguished from paracrine systems among cells of the same type. It is important to distinguish between both, and several theoretical models have recently been presented that show the biological importance of this distinction (see below under ‘The dynamics of autocrine and paracrine systems’). Nevertheless, on the basis of the above four criteria, the most important autocrine loops reported today are made by vasoactive intestinal peptide (VIP), galanin, ET, several growth factors (i.e. TGF-α, TGF-β1, TGF-β3 and FGF-2) and PRL itself. These autocrine loops can be interconnected, resulting in an activation of positive feedforward mechanisms that enable strong auto-activation (Fig. 4). As will be discussed in detail below, the activity of these autocrine loops is strongly context-dependent and the direction of the functional change seen in one condition is sometimes opposite to that in another condition.

### VIP

As repeatedly shown, VIP mRNA and peptide and VIP receptors are expressed in anterior pituitary of several species, including human (355, 356). The peptide is releasable and its expression is regulated by thyroid, gonadal and adrenocortical hormones (183). Moreover, the number of VIP immunoreactive cells in pituitary cell cultures decreases upon dopamine treatment (357), suggesting a link to the protagonist regulatory signal of PRL release. The peptide is located in lactotrophs (358) and in a non-identified cell type with stellate shape (359). After oestrogen treatment, VIP expression is increased and is present in a subpopulation of lactotrophs also containing galanin (289). It is well established that VIP is a PRL secretagogue and the finding that addition of anti-VIP antiserum to cultured anterior pituitary cells decreases PRL release is consistent with a paracrine or autocrine role of the peptide (358–362). There is evidence that the action is at least in part autocrine and not paracrine because the phenomenon is seen in a reverse haemolytic plaque assay set-up (358). The relevance of this *in vivo* is supported by the finding that lactotroph hyperplasia and associated angiogenesis induced by oestrogen *in vivo* can be reduced by treatment with a VIP antagonist (362), indicating that VIP mediates in part the action of oestradiol on lactotrophs. Serotonin-induced PRL release is associated with VIP release and is also blunted in the presence of a VIP antagonist (363). TRH-stimulation of PRL release is associated with VIP release and, when this stimulation occurs in the presence of a VIP receptor antagonist, TRH-induced PRL release is blunted, suggesting an autocrine potentiating role of VIP in TRH action (361). Insulin growth factor (IGF)-I-stimulated PRL release (but not IGF-I-inhibition of GH release) could also be blunted by anti-VIP antibody (360). Also, the rebound in PRL secretion occurring upon withdrawal of dopamine (364) is blunted by anti-VIP antibody (365). Interestingly, the effect of VIP on PRL release itself appears to be mediated by galanin released from a subpopulation of lactotrophs that itself does not secrete PRL in response to VIP (366). Thus, VIP, galanin and IGF-I appear to be linked to each other in a complex autocrine/paracrine network in which VIP is a feedforward system in the local regulation of lactotroph activity by galanin.

### Galanin

This peptide is a well-studied autocrine regulator of PRL gene expression and secretion in a gender-specific manner. It is located mainly in a subpopulation of lactotrophs in female rats, in contrast to its main location in somatotrophs and thyrotrophs in male rats (367). The production of pituitary galanin fluctuates with oestrogen levels during the oestrous cycle, pregnancy and lactation (367, 368), with oestradiol increasing its mRNA level with several orders of magnitude. Oestradiol also increases the number of galanin-positive lactotrophs (366). It is in fact galanin that mediates the stimulatory action of oestradiol on lactotroph proliferation and PRL gene expression. Galanin is releasable and antigalanin antiserum prevents the action of oestradiol on lactotrophs (366). Direct evidence that galanin is acting in an autocrine and not in a paracrine fashion on lactotrophs comes from experiments using the reversed haemolytic plaque assay. Galanin-positive lactotrophs (identified by *in situ* hybridisation) release more PRL than galanin-negative lactotrophs, whereas galanin antiserum significantly blunts PRL secretion from galanin-positive cells. The oestrogen-dependent autocrine action of galanin has been confirmed *in vivo* in transgenic mice with a galanin gene null mutation (369). In female transgenic mice, PRL mRNA levels and hormone content were significantly reduced compared to wild-type controls, leading to a failure in lactation and reduced plasma PRL levels. In the null mice, the proliferative response of the lactotrophs to oestrogen was lost and oestrogen failed to stimulate PRL release. Conversely, in transgenic mice overexpressing galanin in lactotrophs, PRL release and synthesis was increased but only in the presence of oestrogen (370).

### TGF-α, TGF-β1, TGF-β3 and FGF-2

Galanin appears to be linked to another autocrine network that is also recruited by oestrogen for lactotroph mitosis and differentiation. That network includes, besides VIP, as discussed earlier, TGF-α, TGF-β1, TGF-β3 and FGF-2. In rats and mice, lactotrophs produce TGF-α and express the EGF-R (176). Exogenous TGF-α and its homolog EGF have a mitogenic action on lactotrophs (171, 173, 177). Treatment of mouse pituitary cells cultured in serum-free medium with an inhibitor of the EGF-R tyrosine kinase or with antisense TGF-α oligonucleotides reduces the mitogenic effect of oestradiol on lactotrophs (177). As already mentioned earlier, the paracrine gonadotroph–lactotroph connection can only work in context with the TGF-α–EGF-R system for functioning (171). Moreover, oestradiol has a positive feedforward effect as it stimulates TGF-α expression (371). A positive feedforward effect of oestradiol has also been noticed on TGF-β3-producing lactotrophs, as the oestrogen increases both the number of the latter cells and their production of TGF-β3 (372). As immunoneutralisation of endogenously secreted TGF-β3 or blocking endogenous TGF-β3 generation by antisense TGF-β3 oligodeoxynucleotide treatment of cultures reduces the mitogenic action of oestrogen, it is believed that endogenous TGF-β3 mediates the proliferative effect of oestrogen (373, 374). It appears that TGF-β3 does not act directly on lactotrophs because TGF-β3 stimulates lactotroph proliferation in a mixed pituitary cell culture but not in cultures of enriched lactotrophs nor in the RC-4B/C cell line, a pituitary cell line representing all cell types of the pituitary except FS cells (372). The addition of FS cells to this cell line restores the response to TGF-β3 (372). The factor mediating the effect of TGF-β3 appears to be FGF-2 produced by FS cells, because immunoneutralisation of FGF-2 abolishes the effect of TGF-β3 (372). Moreover, oestradiol also favours lactotroph cell proliferation by attenuating an autocrine inhibitory loop. It decreases expression of TGF-β1 and its receptor in a subpopulation of lactotrophs, the number of TGF-β1-containing lactotrophs and PRL secretion (255, 256, 375–377).

The relevance of the TGF-α system has also been evaluated *in vivo* in transgenic mice. Targeting TGF-α overexpression in lactotrophs leads to lactotroph hyperplasia and adenoma formation (378), with no effect on corticotrophs, despite EGF being able to stimulate corticotroph proliferation (175). In another more powerful approach, EGF-R signalling was blocked by overexpressing a dominant negative EGF-R, lacking the intracellular protein kinase domain, into GH and PRL cells. The mutant receptor was targeted by GH and PRL promoters combined with a tetracycline-inducible expression system, that allows expression at a precise age (379). When the dominant mutant gene was overexpressed in GH cells during embryonic life, both somatotroph and lactotroph numbers were strongly depressed in adult life. However, when the dominant negative mutant gene was expressed in the GH cells during the early postnatal period, no change in the adult appearance of the cells was observed. Moreover, when expression of the mutant receptor was started during pregnancy, the typical hyperplastic lactotroph response of pregnancy was maintained (379). These data suggest that EGF-R signalling is only essential for the embryonic expansion or maintenance of the lactosomatotroph lineage and that, later in life, other mechanisms can compensate for the lack of the TGF-α–EGF-R signalling. It should be noticed that initial differentiation into GH cells is not dependent on EGF-R signalling because the expression of the dominant negative EGF-R gene needs an active GH promoter in order to be functional. Thus, TGF-α is required for the expansion of the somatotrophs and lactotrophs and not for their initial differentiation.

TGF-α is first synthesised as an integral transmembrane protein, with the TGF-α sequence present in the extracellular domain. TGF-α and other EGF-like molecules can be enzymatically cleaved and shed into the extracellular space by a metalloproteinase-disintegrin, also expressed as a transmembrane protein and known as ‘TGF-α converting enzyme’ (380) and this processing is usually required in order to show a growth-promoting action (381). Nevertheless, as for several other EGF-like molecules, the TGF-α precursor may also function as an EGF-R agonist in an autocrine manner while still bound to the membrane, and there is evidence that the obtained effect can be different from that of the shed form (382). This may be related to the fact that the EGF-R and the TGF-α precursor are sorted to the same area on the plasma membrane to ensure rapid and efficient recapturing of the shed TGF-α by the EGF-R (381, 383, 384). Exposing the cells randomly to free TGF-α, which occurs when TGF-α is added exogenously, obviously leads to cellular responses that may not be representative of the physiological response to the endogenous ligand.

There is evidence that TGF-α acts in a strict autocrine way because its endogenous action was demonstrated in a reverse haemolytic plaque assay (385). It has even been suggested that the transmembrane form, and not the cleaved form, of TGF-α at the surface of the lactotrophs is the effective form in the pituitary gland (385), but this proposal could be criticised because the method used may have failed to detect small amounts of released TGF-α. It has been shown in other systems that normally processed and cleaved TGF-α may be completely captured by the EGF-R present in the immediate molecular vicinity and become undetectable in the interstitium (386).

Autocrine loops may be essential for preserving specific auto-stimulation of the expressing cell without neighbouring cells are affected. This positional specificity has been assessed by Kudlow's group by comparing mice overexpressing wild-type TGF-α in lactotrophs with mice overexpressing an obligately soluble form of TGF-α in lactotrophs (i.e. a TGF-α molecule lacking the transmembrane region of the gene) (387). Mice overexpressing the soluble form of TGF-α did not display lactotroph hyperplasia, but the pituitary became very large due to proliferation of nonhormonal interstitial cells. These findings led the authors to propose that the specific autocrine mechanism of lactotroph growth requires a membrane bound TGF-α precursor that is shed in a strictly controlled manner, whereas TGF-α shed in large amounts can diffuse widely and act in a paracrine, but nonphysiological manner to stimulate growth of interstitial cells all over the tissue. In the latter studies, however, it is strange that no growth of other glandular cells was seen as these cells also express EGF-R (176) and EGF can stimulate corticotroph proliferation (175). An additional observation by the Kudlow's group, however, was that mice overexpressing the mutant TGFa lacking the intracellular kinase domain developed a normal pituitary, which led the authors to propose that the normal TGF-α precursor may be signalling through its own intracellular domain instead of via the EGF-R autocrine loop (387).

An autocrine EGF-R-implicating loop has also been demonstrated in lactotroph cell lines. Treatment of these cells with the receptor tyrosine kinase inhibitor herbimycin A, markedly reduced basal PRL mRNA levels in a reversible manner, as well as Tyr phosphorylation, and inhibited PRL mRNA gene expression induced by bFGF and TRH (388).

It is important to emphasise here that the VIP-galanin-TGFα–TGF-β1–TGF-β3–FGF-2 system provides a nice example of how an autocrine system functions. It consists of several components that can be recruited by the same factor (oestradiol) in a feedforward manner in order to establish an efficient response. At the same time, stability in the system is preserved by interaction with paracrine substances such as signals from gonadotrophs. Stability and fine-tuning is also reinforced by the existence of an autocrine negative feedback loop by ETs, as explained below, and by redundancy, which can allow compensation in case one of the constituents in the network would be lacking.

### NGF

Another growth factor that may be implicated in autocrine growth of lactotrophs is NGF. According to Missale, NGF is produced in the rat pituitary and selectively expressed in lactotrophs already in early postnatal life and is released by these cells when established in culture (389), although others found NGF also in subpopulations of other cell types (390). Exogenous NGF increased PRL mRNA expression (391, 392) and augmented the number of cells expressing PRL in monolayer cell cultures from newborn rats (391). Immunoneutralisation of secreted NGF completely prevented the generation of lactotrophs (391). Whether the action is strictly autocrine or paracrine on lactotrophs remains unsettled. In reaggregated cell cultures from 14-day-old rats, we also found that exogenous NGF increased mitotic activity in cells identified as lactotrophs and augmented the total number of cells expressing PRL mRNA (393). However, addition of the same antibody that Missale used in her monolayer cultures did not result in a decrease in basal lactotroph expansion in our aggregates, despite it clearly antagonising the action of exogenous NGF (393). A possible explanation of the discrepancy in the findings is that autocrine action of endogenous NGF may be restricted to the neonatal period and that later spontaneous NGF release no longer affects lactotroph cell renewal unless NGF release is specifically activated. Another possible explanation is that, in three-dimensional cultures, the antibody is not efficient enough to capture the released NGF due to a much more restricted and locally aggregated NGF-R–NGF complex, which would require a very high antibody concentration to be broken. NGF secretion has been shown to be activated by IL-1 (394) and VIP (389), whereas GHRH, TNF-α and FGF-2 inhibit it and dopamine completely blocks the VIP-stimulated secretion. Thus, the NGF autocrine loop may be switched on and off according to the context in the microenvironment. Both IL-1 and VIP are molecules that are important during the pituitary response to immune stress. Another possibility is that endogenous NGF can only function in a contextual setting when there is a three-dimensional cellular organisation, and that the contextual setting is not reached in the aggregate culture medium used, leading to a silencing of endogenous NGF. In monolayer culture, NGF could be secreted in an uncontrolled manner due to the loss of intimate cell–cell contact in the latter culture system and, hence, immunoneutralisation would ‘show’ an endogenous NGF activity. We also found that at least part of the action of NGF on lactotroph expansion was through a proliferative effect on lactotroph progenitor cells already expressing Pit-1 but not yet PRL (393). NGF was also shown to be important for normal expression of the dopamine receptor D_2_ (395).

### Endothelins

ET-1 and ET-3-like immunoreactivity and ET-R are expressed in the anterior pituitary (261–263), more precisely in lactotrophs (396, 397). The peptides are secreted as shown in a reverse haemolytic plaque assay (398). The functioning of endogenous ET is highly context-dependent with time, with steroid hormones and dopamine determining the direction of the secretory response to ET. The prominent action of exogenous ET is profound inhibition of PRL secretion (268, 399–401) but, when studied in a perifused cell column, ET-1 initially induces a prompt and short-lasting increase in PRL release, which is followed by a profound sustained inhibition (402). A similar response was seen for GH release (230). The autocrine action of ET via the ET(A)-R has clearly been documented in a reverse haemolytic plaque assay (398). The ET(A)-R antagonists BQ123 and BQ610, and the ET convertase enzyme inhibitory peptide, [22Val]big ET1_(16−38)_, increase basal PRL secretion, whereas the ET(B)-R antagonist BQ788 was ineffective. A peculiar phenomenon in ET action is that, after long-term (48 h) exposure to dopamine in culture, the inhibitory component of ET-1 on PRL secretion reverses into a stimulatory one (403). The stimulatory versus inhibitory component also appears to depend on gender and oestrogen status. Blocking the ET-mediated autocrine loop with the ET(A)-R antagonist resulted in an increase in PRL secretion when pituitary cells were obtained at pro-oestrous, oestrous, and dioestrous-1, whereas PRL secretion was decreased by the antagonist at dioestrous-2 (404). Importantly, the authors found that the concentration–response curves of the stimulatory effect of the ET(A) antagonist were bell-shaped at pro-oestrous and dioestrous-1 but that, at oestrous, the dose–response was monophasic, indicating that endogenous ET at pro-oestrous and dioestrous-1 is both stimulatory and inhibitory depending on receptor occupancy and that, at dioestrous-2, endogenous ET is predominantly stimulatory. These findings led the authors to propose that, at oestrous, the autocrine negative feedback by ET may play a role in restraining PRL secretion following the oestradiol-induced pro-oestrous PRL surge. The gonadal steroid modulation of the lactotrophs ET system is further illustrated by the finding that ET(A)-R antagonism did not affect PRL secretion in cultured cells obtained from progesterone-implanted ovariectomised animals but increased PRL secretion in the cells from oestradiol and oestradiol + progesterone-treated groups (405).

Once released, ETs may also target other autocrine networks or may be itself a target of other networks. ETs release Substance P (406) and ET-3 release is augmented by IGF-I and inhibited by TGF-β, whereas ET-1 secretion is augmented by TGF-β (262). Thus, through altering the availability of ET, these interactions may trigger feedforward inhibition or, depending on the interacting substance, exert negative feedbacks.

As already discussed earlier, ET peptides are also present in gonadotrophs and somatotrophs and could therefore affect lactotrophs in a paracrine manner (199). Moreover, ovarian steroid hormones have a differential effect on the distribution of ET over these cell types (199): the number of ET-1 immunoreactive pituitary cells in ovariectomised rats was unaffected by prior *in vivo* treatment with progesterone alone whereas treatment with oestradiol slightly decreased the number of ET-1-positive lactotrophs and somatotrophs but increased the occurrence of ET-1-positive gonadotrophs. Combined treatment with oestradiol and progesterone robustly increased the proportion of ET-1 immunoreactive lactotrophs and gonadotrophs but had no effect on somatotrophs. Thus, the ovarian oestrogen signal augments the paracrine action of gonadotrophs on lactotrophs whereas oestrogen and progesterone in combination augment autocrine signalling in lactotrophs.

## Autocrine regulation of somatotrophs

**Fig. 5:**
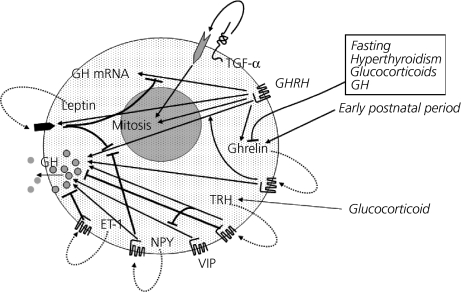
Schematic representation of autocrine loops acting in somatotrophs. Full lines indicate pathways for which experimental criteria for autocrine action have been largely met. Interrupted lines are hypothetical interactions proposed on the basis of the presence of the indicated factors in the somatotroph and their pharmacological effects on the same cell. →, Stimulatory effect; ⊥, inhibitory effect; ET, endothelin; GH, growth hormone; GHRH, growth hormone-releasing hormone; NPY, neuropeptide Y; TGF, transforming growth factor; TRH, thyroid-releasing hormone; VIP, vasoactive intestinal peptide.

Whereas autocrine regulation of lactotroph function is a target of oestrogen and related to reproductive functions, it can be expected that autocrine regulation of somatotrophs may be related to energy homeostasis because GH is one of the protagonist players in energy expenditure, an action performed in concert with TSH and also PRL. Molecules playing a central role in food intake regulation at the hypothalamic level, such as ghrelin, leptin, NPY and TRH, are also expressed in somatotrophs and operate directly in the pituitary (Fig. 5). However, to date, there is only indirect evidence for autocrine regulation by these peptides.

### Ghrelin

Ghrelin, a 28-amino-acid acylated peptide originally isolated from the rat stomach, displays manifest orexigenic activity (407). In the rat pituitary, it is expressed in somatotrophs, lactotrophs and thyrotrophs, but not in corticotrophs and gonadotrophs (408). Ghrelin and GH secretagogue receptor GHS-R1a mRNA expression were also detected in GH3 cells (409) and in human pituitary (410). Ghrelin strongly stimulates GH release (407). Endogenous pituitary ghrelin appears to play a physiological role in GH release because the GHS-R-specific antagonist [d-Lys-3]-GHRP-6 significantly reduces GHRH-stimulated GH release *in vitro* (411). Thus, local ghrelin is a positive feedforward system for GHRH action by sensitising the somatotroph to GHRH. Local regulation is also adapted for such a role because ghrelin mRNA and peptide content change in the same direction as GHRH content in the hypothalamus under various conditions. For example, GH treatment, glucocorticoid excess, hyperthyroidism and food deprivation decrease hypothalamic GHRH and pituitary ghrelin mRNA and peptide levels, whereas the opposite was found in dwarf rats, and during glucocorticoid deficiency and hypothyroidism (408, 411). GHRH treatment increases ghrelin expression in the pituitary (408, 412).

Of note, pituitary ghrelin expression fluctuates developmentally (408), being highest at E18 in rats and then declining with age (412). GHS-R expression is also high in fetal and neonatal life, decreases postnatally and increases again just before puberty to decrease again later (413). Moreover, at early postnatal age, somatotrophs are more sensitive to GHRH (414–417). These high early postnatal levels of ghrelin and ghrelin receptor correlate with higher GH levels at late embryonic and early postnatal ages (418, 419), when hypothalamic GHRH is still low, suggesting a more important local role of pituitary ghrelin system in releasing GH at that young age and possibly also at puberty. Ghrelin is also able to increase Pit-1 gene transcription in neonatal rat anterior pituitary cells (420), suggesting a role in pituitary somatotroph development.

It is interesting to relate these findings to the age-dependent differences in sensitivity of GH secretion *in vitro* in response not only to GHRH, but also to angiotensin II and TRH. We found that all these peptides have dual effects on GH release in aggregate pituitary cell cultures, with the response being predominantly stimulation in neonatal life and inhibitory after puberty (229, 232, 233). Glucocorticoids enhanced the stimulatory component but only in aggregates from prepubertal rats; at adult age, glucocorticoids increased the inhibitory effect (229). It is tempting to speculate that the occurrence of a strong GH response to angiotensin II in the presence of glucocorticoids in neonatal life may be related to the high level of ghrelin at that age, and the increased level of GHS-R1a known to be induced by glucocorticoids (421). In favour of the implication of a paracrine system in establishing the GH releasing effect of angiotensin II is the finding that the GH response to angiotensin II disappears when pituitary cells are used dispersed in a cytodex bead cell column, whereas the PRL response is preserved (229). The stimulation of GH release by VIP in the presence of glucocorticoids (233) is perhaps also related to the ghrelin system.

It is interesting to relate pituitary ghrelin function to pathophysiological phenomena occurring during protracted critical illness in humans. In these patients, the synchrony among pulsatile GH, PRL and TSH secretion is lost and pulse height is depressed. Upon infusion, GHRP-2, a synthetic GH secretagogue acting through the ghrelin receptor, restores these pulses and synchronisation to some extent (422). As ghrelin is expressed not only in somatotrophs, but also in a subpopulation of lactotrophs and thyrotrophs (at least as studied in the rat), it would be worthwhile to test the hypothesis that the local pituitary ghrelin system is depressed in these patients, leading to a depression of pulses and hormone peak magnitude. It is also noteworthy that, in anorexia nervosa, there is a decreased sensitivity to the GH releasing action of ghrelin even though ghrelin plasma levels are increased (423).

### TRH

Whereas thyroid hormone increases appetite, TRH has central effects reducing food intake (424). TRH has also been detected within the anterior pituitary. Gwen Childs was the first to notice the presence of TRH immunoreactivity in the intact rat anterior pituitary in secretory granules of thyrotrophs and PRL cells (202). The presence of the proTRH mRNA was later shown in a subpopulation of somatotrophs by Bruhn *et al*. (425). In culture (from 2-week-old rats), TRH expression was stimulated by thyroid hormone and potentiated by glucocorticoids (426), whereas TRH gene expression was undetectable in cultures deprived of glucocorticoid (427). Cultures from female rats contained and secreted higher amounts of TRH than those from males (428).

Although there is ample evidence for a secretagogue action of TRH in GH, TSH and PRL release, an autocrine action on somatotrophs remains to be demonstrated. Exogenous TRH is capable of stimulating GH secretion *in vivo*, but this is only under particular developmental, experimental or pathological conditions (429, 430). TRH stimulates GH release in neonatal rat pituitary *in vivo* (431) and in cell culture (232, 417) whereas, in adults, it inhibits GH release stimulated by VIP *in vitro* (233). Stimulation *in vitro* has also been observed in pituitary tissue from adult hypothyroid rats (as is the case in hypothyroid humans) (432) or in pituitary tissue from euthyroid rats after pre-exposure to GHRH (433). The TRH-1-R has been detected by *in situ* hybridisation in approximately 50% of the GH cells (434) as well as in human GH adenomas (429). In lower vertebrates (birds and amphibians), TRH is a prominent GH secretagogue, particularly in immature chicken (435). In adult chicken, the GH releasing action of TRH depends on the feeding status of the animal (435) and can also be evoked *in vitro* in the presence of GHRH (436).

Although there is no experimental evidence that TRH is an autocrine GH secretagogue, there is circumstantial evidence for a paracrine action on TSH secretion (437). The latter may be important for TSH release in concert with GH release for the control of energy expenditure. When cultures are treated with disulfiram, an inhibitor of the C-terminal amidation of glycine-extended TRH precursor, the accumulation of TRH is drastically lowered, as is TSH release (437). However, release of GH was not affected, suggesting that no autocrine loop of TRH on GH secretion is operative under conditions where it was acting in a paracrine manner. The latter observation again emphasises the importance of contextual conditioning of autocrine and paracrine interactions.

### Leptin

Leptin is a hormone secreted by adipocytes that signals the energy reserve status stored in fat to hypothalamic centres regulating satiety and energy expenditure. It negatively affects food intake and body mass and increases metabolic rate, psychomotor activity and body temperature (438, 439). A sufficient leptin signal is also essential for normal activity at all levels of the HPG axis (439). Leptin (440, 441) and leptin receptor (440) have also been located in the pituitary gland in several species including humans (442–444). In the rat, leptin is found in subpopulations of somatotrophs, gonadotrophs, thyrotrophs, corticotrophs and FS cells but very little in PRL cells (200), whereas other studies found it only in somatotrophs and gonadotrophs (201), and still others in thyrotrophs (440) or in gonadotrophs and less in thyrotrophs (445). In humans, leptin was found in somatotrophs, gonadotrophs, thyrotrophs and corticotrophs but, again, not in lactotrophs (446). Thus, it appears that leptin is expressed in all hormonal cell types, except lactotrophs. By contrast, the leptin receptor is distributed more restrictively. In the rat, most somatotrophs express leptin receptor, whereas only 1% of the other cell types is leptin receptor-positive (445). It therefore appears that local leptin signals converge the needs of the different hypothalamic-pituitary-peripheral axes to the somatotrophs. Such a convergence is of particular importance whenever there is a need for adaptation in energy homeostasis. Indeed, during starvation, there is a concerted adaptation in the GH axis, the HPT axis and the HPA axis. Leptin plasma levels fall during starvation and this is the physiological stimulus for suppression of pro-TRH mRNA expression in the paraventricular nucleus within the hypothalamus, which in turn will result in decreased activity in the HPT axis in order to save energy (447). During starvation, the TSH response to TRH is diminished and, in most species, there is an increase in GH secretion with a decrease in IGF-1 levels and a rise in GH responsiveness to GHRH (448).

A contribution of the pituitary leptin system for the sake of decreasing metabolic rate but, at the same time, favouring GH output during starvation would require that starvation would lead to less leptin action in somatotrophs, which in turn would lead to an increase in the sensitivity to GHRH. However, to date, no studies have demonstrated a fall in pituitary leptin levels during starvation. Nevertheless, studies with leptin treatment in sheep and swine already revealed actions of leptin that are compatible with a pituitary leptin contribution in the right direction. In sheep, treatment with leptin for 1–3 days reduces mRNA levels of GH and GHRH-R in the pituitary, and decreases the GH secretory response to GHRH (449). In pig pituitary *in vitro*, leptin acutely increases GH secretion and, as in sheep, inhibits GHRH-stimulated GH release (450, 451). Thus, in case leptin levels decrease, such as during starvation, the opposite is expected to occur: a rise in GH output and sensitivity to GHRH. Whether endogenous leptin acts in a similar manner in an autocrine or paracrine way on somatotrophs still remains to be demonstrated.

Nevertheless, pituitary leptin reserves appear to adapt to changes in the HPG axis in a cell type-specific manner. The proportion of leptin-positive somatotrophs increases from dioestrous to pro-oestrous (442) and the number of leptin-positive cells increases after short-time treatment *in vitro* with GHRH and oestrogen (452).

Another potential role of pituitary leptin may be related to the GH axis during development because leptin can stimulate GH secretion at the level of the pituitary in human fetal pituitary *in vitro* (442). By contrast, no such effect was seen in adult rat pituitary when the GH axis is no longer necessary for growth (453).

### Other peptides

Various other peptides have been shown to be present in somatotrophs such as enkephalin (454), NPY and Substance P (particularly in male rats) (183, 455).

NPY has been reported to stimulate basal GH release (197) but to block the GH response to GHRH in porcine pituitary cells (231). Based on the prominent action of NPY at the hypothalamic level in regulating feeding and energy consumption (456) and the role of GH in energy homeostasis, the question can be raised as to whether NPY may participate in such actions by modulating GH secretion at the pituitary somatotroph level.

No effect of Substance P on GH release has been found but several Substance P antagonists reduced the stimulation of GH release by GH-releasing peptides (ghrelin receptor agonists) but not GHRH (457). An inhibitory effect of Substance P antagonists on basal GH release was also reported by us in aggregate cell cultures (458). However, the latter study also showed nonspecific actions of these antagonists. The presence of Substance P in somatotrophs has also been detected in the porcine pituitary (459). Importantly, food restriction during pregnancy increases the activity of the GH axis in the fetus, including an increased somatotroph cell size and the appearance of a higher proportion of somatotrophs expressing Substance P (459). GHRH is known to increase Substance P in the pituitary (460). The significance of these findings is unknown but is intriguing considering that Substance P was found to be ineffective with respect to *in vitro* GH secretion (457, 461).

## Autocrine regulation of gonadotrophs

**Fig. 6:**
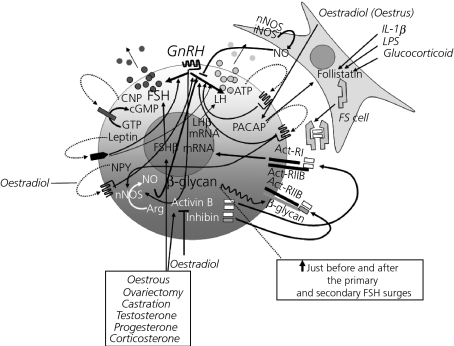
Schematic representation of autocrine loops acting in gonadotrophs. Full lines indicate pathways for which experimental criteria for autocrine action have been largely met. Interrupted lines are hypothetical interactions proposed on the basis of the presence of the indicated factors in the gonadotroph and their pharmacological effects on the same cell. →, Stimulatory effect; ⊥, inhibitory effect; Act-R, activin receptor; CNP, C-type natriuretic peptide; FS, folliculo-stellate; FSH, follicle-stimulating hormone; GnRH, gonadotophin-releasing hormone; IL, interleukin; iNOS, inducible nitric oxide synthase; LH, luteinising hormone; LPS, lipopolysaccharide; nNOS, neuronal nitric oxide synthase; NO, nitric oxide; PACAP, pituitary adenylate cyclase-activating peptide.

Evidence for autocrine control of gonadotroph function is substantial, particularly with respect to the differential regulation of FSH and LH secretion. Various signalling molecules participate, forming a complex network (Fig. 6). Importantly, there is evidence for cross-talk between at least certain autocrine substances, such as between NO, CNP and leptin and between PACAP and the activin–follistatin system.

### The activin–inhibin–follistatin system

Activin and inhibin are growth and differentiation factors belonging to the TGF-β family. Members of this family are involved in many developmental and functional processes of many cell types in normal as well as tumoural state. The system plays an important role at all levels of the HPG axis (462). Inhibin consists of an α-subunit (inhibin-α) and a β-subunit, which can be either a βA or a βB isoform (inhibin A and inhibin B). Activins are dimers consisting only of the inhibin β-subunits [i.e. either two identical βA isoforms (activin A), two identical βB isoforms (activin B) or a hetero βA/βB complex (activin AB)]. Activin βA and βB as well as the activin receptor and coreceptors are expressed in the anterior pituitary and anterior pituitary cells secrete activin A and B (463). In mammals, the main site of synthesis is within the gonadotrophs (462). Also inhibin-α is expressed together with the β-subunits in rat gonadotrophs (464), although secretion of native inhibin B from these cells has not been demonstrated yet. In frogs, activin subunits are located in thyrotrophs, gonadotrophs and somatotrophs (465, 466). In fish both βA and βB subunits have been located in somatotrophs and not in gonadotrophs and inhibin-α is located mainly in nerve endings (467), suggesting that in fish pituitary mainly native activins are operative. In mammals, activin A and B stimulate FSH synthesis and secretion in pituitary cell cultures (468) and there is essentially no effect on LH release except under particular conditions (469). In frogs, activins stimulate FSH as well as LH production and inhibin blocks both these effects (466). In fish, activin A and B as well as inhibin stimulate GH secretion (467), which is interesting in view of the protagonist role of GH in gonadal function in that animal class. Moreover, in cultured gold fish pituitary cells, activin B stimulates the gonadotrophin GTH-Iβ but suppresses GTH-IIβ mRNA levels (470). As shown in mammals, inhibin diminishes FSH production by obliterating the action of endogenous activin at its receptor, for which the coreceptor β-glycan is obligate (471, 472). When activin and inhibin are added to pituitary cell cultures simultaneously, FSHβ mRNA becomes undetectable (473), suggesting that inhibin is dominant over activin in regulating FSHβ expression.

#### Autocrine activin

Activin is acting in an autocrine loop in both basal and specified physiological situations and represents one of the fundamental mechanisms for selective regulation of FSH expression and secretion under the governance of a single GnRH. By using a specific anti-activin B (not cross-reacting with activin A or inhibin) monoclonal antibody, it has been shown that activin B from gonadotrophs in culture exerts a tonic stimulatory influence on FSHβ mRNA levels and FSH secretion, because adding this antibody to cultures resulted in a decrease in basal FSHβ mRNA levels and FSH secretion without affecting secretion of LH (468). The same antibody fully blocked the stimulation of FSHβ expression elicited by added activin B but not the inhibition by added inhibin (albeit it was smaller in magnitude) (468). Similarly, in castrated mice in which the gene for activin receptor type II (Act-R-II) was disrupted, FSHβ expression in the pituitary is lower than in castrated wild-type mice (474).

Several known effects of steroids on FSHβ expression appear to be mediated at least in part by activin B released from the gonadotrophs after application of these hormones. Testosterone, progesterone or glucocorticoid treatment increased FSHβ levels in cell cultures and, again, this effect can be blocked with the monoclonal antibody against activin B (463, 475–478). Immunoneutralisation studies also showed that activin B mediates the rise in FSH after ovariectomy *in vivo* in the rat (479–481) and that, in sheep, oestradiol depresses pituitary FSHβ expression, at least partly via inhibition of activin B expression (482). The latter findings are particularly relevant as the ovine FSHβ promoter has an oestrogen responsive region, but not an oestrogen response element, suggesting that oestrogen regulates FSH expression indirectly (483) via repression by the latter of the activin βB subunit gene.

Even though the above experiments provide strong evidence for a local control of FSHβ expression and FSH secretion by activin B, they leave the question open whether activin B acts in a strict autocrine manner or diffuses to neighbouring gonadotrophs to exert its effect in a paracrine fashion. Some indirect evidence has been found, however, that the action of activin is at least in part autocrine. Using a cell blot assay test system, in which the secretion of single cells can be separately explored and in which no cell–cell contact exists, it was found that testosterone, known to increase FSH production via activin B, does not increase the number of FSH-secreting cells but significantly augments the amount of FSH secreted from the FSH-positive cells (484). Interestingly, the largest secretors did not increase their output upon testosterone application.

Several physiological events appear to operate through alterations of activin B expression. Whereas the pro-oestrous rise in FSH and LH secretion is dependent on the GnRH pre-ovulatory peak, the second FSH rise on oestrous, which is important for recruitment of follicles, is independent on hypothalamic GnRH input (485, 486). It is the latter selective FSH rise that is dependent on activin, as treatment with the activin B monoclonal antibody on the evening of pro-oestrous attenuated the rise of serum FSH on early oestrous (480). The second FSH peak is intrinsic to the pituitary because it persists in culture when pituitaries are isolated at the time that the second FSH peak would have occurred (487). Another argument in support for activin-controlled FSH secretion is the finding of FSH hypersecretion after ovariectomy in hypophysectomised rats receiving a pituitary graft under the kidney capsule and of inhibition of this secretion by quenching the action of activin by follistatin (479). Activins are also permissive for the stimulation of FSH production by GnRH (488, 489).

Activin appears to be involved in the juvenile FSH rise in the female rat. As already mentioned, it is well documented that FSH plasma levels in rats increase to very high levels between the end of the first and second postnatal week, particularly in females (88–90), and that the gonadotroph cell population expands rapidly to proportions never obtained in later life (91, 490), raising the intriguing question of whether this developmental change is also mediated by activin B. Some answers to this question were given by Wilson and coworkers (491, 492). They showed that activin βA subunit mRNA reaches a peak level at 10 days of age and then falls gradually to an adult level at day 21, whereas βB subunit mRNA level displays a marked peak value at day 10 and has already fallen to normal adult level by day 12. Moreover, Act-R-II mRNA was found to be expressed diffusely over the entire anterior pituitary whereas mRNA of Act-R-IIB, which is the preferred receptor for activin B, was almost exclusively found in gonadotrophs, but there was no apparent change in the level of Act-R-IIB during the second and third week of life. These data suggest at least the possibility that activin B can have an autocrine/paracrine role for up-regulation of FSHβ expression during the second week of postnatal life. Furthermore, experiments with androgen and oestrogen antagonists *in vivo* and with androgen agonists in pituitary cell culture have clearly shown that oestradiol and testosterone are responsible for the rise in FSH production at 2 weeks of life (492). Moreover, plasma levels of oestradiol (493) and binding of oestradiol to its receptors (494) in the anterior pituitary increase dramatically around postnatal day 10, declining slightly thereafter, and the expression level of oestradiol receptor-β mRNA is much greater in the gonadotrophs of immature than of adult female rats (495). An additional sensitisation mechanism for FSH production at 1–2 weeks of life is that the conversion of testosterone to 5α-dihydrotestosterone, which is the preferred form for binding to the androgen receptor and the preferential androgen to stimulate FSH production (496), also increases dramatically between days 10 and 15 of life (497), with this conversion occurring mainly in the gonadotrophs (498).

#### Autocrine inhibin

The question whether there is also a negative tone of pituitary inhibin on FSH secretion has not been definitely answered, but there are data supporting this view. Kumar *et al*. (474) compared Act-R-II null mice, which are unable to respond to endogenous pituitary activin, with inhibin-α null mice, which cannot make inhibins; these mice were also castrated to eliminate circulating inhibins from gonadal origin. The inhibin-α null mice showed enhanced levels of FSH plasma levels but the level of FSHβ mRNA in the pituitary was unchanged. Furthermore, in mice that lacked both inhibin-α and the Act-R-II genes (474), FSHβ mRNA levels were as low as in the mice with only the Act-R-II gene disrupted and the absence of inhibin increased serum FSH levels both in the presence and absence of the Act-R-II (474).

#### Paracrine/autocrine follistatin

An important paracrine/autocrine modulator of activin-regulated FSHβ gene expression and FSH secretion is follistatin. This glycoprotein binds activin and in this way masks the binding domain of the activin molecule, preventing it from binding to the Act-R-II; consequently, activation of Act-R-I does not occur (472), resulting in bio-neutralisation of activin. Follistatin has been shown to be present in and secreted by pituitary FS cells (499). It was later found that several pituitary cell types, including gonadotrophs, also express follistatin (500–503). At midcycle, follistatin is expressed mainly by LH gonadotrophs, but it is expressed by all other hormonal cell types earlier in the cycle (503). The local availability of follistatin appears to be important in orchestrating the efficacy of locally secreted activin B to drive basal FSH secretion (502, 504). GnRH, gonadal steroids and plasma all decrease the expression of follistatin (481, 505, 506), as shown by GnRH immunoneutralisation and GnRH antagonist treatments and by gonadectomy experiments, providing evidence that these substances are physiological regulators of follistatin. Activin and PACAP stimulate follistatin expression (507).

Another level of differential FSH and LH regulation is via changes in GnRH pulse patterns. At least in part, these patterns are translated in changes in inhibin, activin βB and follistatin expression that in turn change the FSH : LH ratio (508). During the oestrous cycle in the rat, pituitary follistatin levels are high before the primary gonadotrophin surge, decrease on pro-oestrous evening, and rise again at midnight on pro-oestrous before returning to basal levels on oestrous morning when the second FSH peak occurs (509, 510). The peak in follistatin mRNA levels precedes the peak in FSHβ gene expression by 6 h (510). The fall in serum inhibins, together with pituitary follistatin following the primary surge, is thought to facilitate the generation and bioavailability of the secondary surge of FSH on oestrous morning, necessary for new follicle recruitment for the next cycle (511, 512).

### The NO system

NO is a gaseous transmitter that is produced intracellularly from l-arginine through the enzyme NO synthase (NOS). NO plays an important regulatory role in many tissues including the neuroendocrine system (513). There are three forms of NOS: type 1 (neuronal) (nNOS) and type 3 (endothelial) (eNOS) are constitutively expressed and Ca^2+^-calmodulin-dependent, whereas type 2 is inducible (iNOS) and Ca^2+^-independent. All three types are expressed in the anterior pituitary (514, 515).

#### Distribution and regulation

nNOS is expressed primarily in gonadotrophs and FS cells (516, 517). There are important gender differences in cellular distribution, and expression changes according to reproductive status. In male rats, nNOS resides mainly in FS cells, whereas, in females, it is mainly in LH cells (518). Gonadectomy causes nNOS up-regulation in LH cells and down-regulation in FS cells and this can be reversed by testosterone or oestradiol treatment (517–519). The effect of gonadectomy is mediated by endogenous GnRH, as it was abolished by treatment with a GnRH antagonist and the GnRH antagonist also decreased basal NOS expression (519). A GnRH agonist increased nNOS expression but only in gonadotrophs and not in FS cells (519). nNOS activity fluctuates with the oestrous cycle and pregnancy/lactation. A steep rise in nNOS expression in gonadotrophs (but not FS cells) occurs during the afternoon of pro-oestrous and, again, this effect is abolished by treatment with a GnRH antagonist (520). In lactating rats, LH cells overexpress nNOS, whereas LH cell size decreases and serum LH levels become low (518).

#### Actions of NO

The change in nNOS expression and the concomitant depression of LH cell activity during lactation both suggest a role of the NO system in down-regulating the HPG axis. At the hypothalamic level, however, NO stimulates GnRH secretion whereas, in isolated rat pituitary or dispersed cells, NO stimulates basal LH and FSH secretion, with both actions being through a cGMP-independent mechanism (517). By contrast, NO appears to inhibit GnRH-stimulated LH release at the pituitary level. Several workers found that the NO donor SNP significantly reduced GnRH-induced LH secretion, whereas the NOS inhibitor Me-Arg potentiated it (516, 517, 521). These data support an intracrine or autocrine negative feedback loop on GnRH stimulation of LH release (but not basal LH release). However, anterior pituitary of pro-oestrous rats made NO-deficient by chronic treatment with a NOS inhibitor showed a lower LH response to GnRH than that of normal controls (522). The effect of an NO donor and a NOS inhibitor had clear effects on the LH response but they depended on whether the pituitaries were from normal or NO-deficient rats, indicating the complexity of the local NO actions (522). Possibly, NO can be inhibitory and stimulatory, and it has been suggested that this may depend on the cellular source of NO (518). NO from FS cells may inhibit GnRH-stimulated LH release as, in cocultures of gonadotroph-enriched populations with FS cell enriched populations, the LH response to GnRH is blunted (94). Another aspect suggesting the complexity of the NO system in the gonadotrophs was revealed by looking at the mechanism of action of NO. Most NO actions are established by activation of soluble guanylate cyclase by NO, resulting in a rise of cGMP (523). However, in isolated rat pituitary cells, the stimulatory action of NO on basal LH and FSH secretion is through a cGMP-independent mechanism (517). Moreover, cGMP does not affect LH release (524). It is known that GnRH stimulates cGMP levels in pituitary *in vitro* (blockable by a NOS inhibitor) (520) and that treatment of pituitary slices with NO donors results in the appearance of cells expressing cGMP, mostly gonadotrophs but not FS cells (525). Together, these data support a cross-talk between GnRH and the gonadotroph NO system on cGMP-dependent processes. Below, whether another autocrine cross-talk via cGMP could be established by the natriuretic peptide CNP in the gonadotrophs is discussed.

The importance of the pituitary nNOS system has also been illustrated by the finding that the nNOS promoter contains the transactivation domain AF-2 of steroidogenic factor-1 (SF-1), an essential transcription factor for gonadotroph differentiation. Deletion of this promoter fragment strongly inhibits nNOS promoter activity (526). The importance of nNOS has also been assessed *in vivo* in mice with targeted disruption of the nNOS gene. Although serious hypofertility was found (fewer oocytes in the oviducts), pituitary responsiveness to GnRH was intact, suggesting that compensatory mechanisms can take over control at the pituitary level (841).

In humans, an interesting interaction of the NO system activated by GnRH has been observed. *In vivo* treatment with a NOS inhibitor attenuated the LH and FSH response to GnRH but this characteristic was lost when simultaneously treated with oxytocin whereas, by itself, oxytocin did not affect the response to GnRH (527)

Interestingly, in frog pituitary, NO increases both basal and GnRH-stimulated LH release (528) and, in goldfish, in which GH is even more essential than in mammals for gonadal function, GnRH stimulates GH secretion and this effect also is attenuated by endogenous pituitary NO (529).

### CNP

CNP is the third member of the NP family, comprising ANP, BNP and CNP. As all NPs, it acts via a one-pass transmembrane receptor with intrinsic guanylyl cyclase (GC) activity, located in the intracellular domain (339). The highest tissue concentration of CNP is found in the anterior pituitary where CNP is synthesised in a subpopulation of gonadotrophs (192). The A-type (GC-A) and B-type (GC-B) receptors are selectively activated by ANP and CNP, respectively, and are both expressed in the pituitary as well (192, 530, 531). Since the GC-B mRNA is detectable in gonadotrophs, CNP is a candidate autocrine regulator of gonadotrophs (192). Such an autocrine action remains to be experimentally demonstrated. However, the actions of exogenous CNP have been explored in primary pituitary cell cultures and in the gonadotrophic cell line αT3-1 (192, 532), and these observations indeed support a putative autocrine role. Strikingly, CNP robustly increases cGMP levels and the intracellular free Ca^2+^ concentration, but does not affect GnRH-stimulated LH release. Nevertheless, GnRH reduced CNP-stimulated cGMP accumulation by a protein kinase C-mediated mechanism, suggesting that there is cross-talk between the GnRH signal transduction pathways and the CNP-activated pathways. Secondarily, a cross-talk via cGMP may also be generated with the gonadotroph NO system. Interestingly, ANP is also expressed in gonadotrophs and does stimulate LH and FSH release (216).

### Leptin

Since leptin is present in gonadotrophs (200, 201, 445, 446) and the number of gonadotrophs that contain leptin increases at pro-oestrous and during pregnancy, and since GnRH stimulates leptin secretion in cultured pituitary cells (201), an autocrine function may exist. Leptin has been shown to stimulate basal and to magnify GnRH-stimulated LH and FSH release from rat pituitary *in vitro* and from rat (218, 533, 534) and sheep (535) pituitary cell cultures. However, in rats, the leptin receptor has been detected mainly on somatotrophs (236), a finding pleading against an autocrine action of leptin in gonadotrophs, unless leptin receptors on gonadotrophs fell below detection limits in the respective study. In sheep, leptin receptors have indeed also been located in gonadotrophs (536).

Interestingly, there may be a connection between leptin and the NO system, described above, as leptin-induced LH release from isolated pituitary appears to be mediated by NO, with the effect being most pronounced in pituitary from pro-oestrous female rats (537, 538).

Final proof of leptin autocrine/paracrine action by immunoneutralisation experiments still needs to be given.

### NPY

Although NPY is secreted in portal blood (539), it is also synthesised in the pituitary gonadotrophs and released from this source, as mentioned earlier. Several research groups have shown that NPY is essential for ovulation. At pro-oestrous, its synthesis in the arcuate nucleus and its release into portal blood is increased (540). At the pituitary level, NPY augments the magnitude of the LH response to GnRH during the pre-ovulatory LH surge (541). For this purpose, NPY appears to act only when the appropriate endocrine milieu that exists just before ovulation is present. *In vitro*, NPY augments GnRH-stimulated LH secretion from anterior pituitary removed from pro-oestrous, but not metoestrous, rats (542). When NPY action is blocked by infusion of anti-NPY antiserum (539) or by deleting the NPY gene in transgenic mice (543), the ovulatory LH surge is significantly attenuated. In pentobarbital-blocked rats, ovulation can only be restored by combined treatment with GnRH and NPY (544). One mechanism of sensitisation of the response to NPY is enhancement of the expression of the NPY receptor Y1 under the influence of the pre-ovulatory increase in oestrogen secretion (541, 545). Thus, the well known positive feedback of oestrogen is realised partly via augmentation of NPY action. NPY also acts as a facilitatory stimulus for the onset of puberty, as shown by the effect of immunoneutralisation of NPY on the release of LH and LHRH (546).

Before we can establish NPY as an autocrine factor, it remains to be demonstrated whether immunoneutralisation of NPY in pituitary cell cultures or selective deletion of NPY expression in the pituitary decreases the LH response to GnRH.

### PACAP

Although PACAP is present in portal blood, it may also have an autocrine action on gonadotrophs, as already above. PACAP is transiently present in the gonadotrophs during pro-oestrous (187, 188, 547). The peptide stimulates LH release in pro-oestrous pituitary (548). Treatment with oestradiol or oestradiol + progesterone rapidly enhanced basal and GnRH- or PACAP-stimulated LH secretion, whereas prolonged treatment with oestradiol and progesterone reduced the response to GnRH (549). The peptide was also reported to increase expression of αGSU but to depress FSHβ (550). The latter appears to be brought about by stimulating follistatin gene transcription in the FS cells that bear PACAP receptors (550, 551). Thus, PACAP may set the balance between LH and FSH secretion in favour of LH.

It is noteworthy that PACAP interacts with the NO system by increasing nNOS expression and potentiating the cGMP rise in response to GnRH (552); NADPH diaphorase staining revealed that these changes occurred in gonadotrophs (552).

### Endothelins

As already mentioned, ETs have been located in gonadotrophs and are releasable as far as tested in monolayer cell cultures. ET receptors are found on αT3-1 cells and ET1 has been shown to acutely stimulate intracellular Ca^2+^ and LH secretion in cultured pituitary cells (553). These data should prompt studies investigating whether the immunoneutralisation of ET would lead to attenuated LH release.

### ATP

The purine ATP, and the derivatives ADP and adenosine, are known to induce various responses in a number of biological systems (554). ATP is coreleased with neurotransmitters and hormones during exocytosis and is believed to augment the exocytotic process through an autocrine positive feedback. Also in the anterior pituitary, ATP is costored with hormones in secretory granules and cosecreted (555, 556). The gland has been shown to express different subtypes of purinergic receptors: P1-R, P2Y-R and P2X-R, in a cell type-specific manner (557) and their activation leads to amplification of intracellular free Ca^2+^ responses to secretagogues (558). The pituitary is also equipped with enzymes (ectonucleotidases) that degrade extracellular ATP (559). The enzymatic cascade generates ADP, the primary agonist for some P2Y-Rs, and adenosine, the agonist for purinergic P1-R (560).

GnRH enhances ATP release from dispersed pituitary cells (555, 556). Addition of ATP to cultured pituitary cells produces a rapid increase in intracellular free Ca^2+^, blockable by a P2Y-R antagonist (561). The effect was seen in identified gonadotrophs and in the gonadotrophic cell line αT3-1. In perifused pituitary cells ATP promptly caused a more than ten-fold rise of basal LH release (556), with an agonist-order of potency typical for the P2Y-R. Gonadotrophs were also reported to express functional P2X-Rs, which are ion-channel receptors (562), that may have a role as pace-making channels (563). Through these receptor channels, ATP induces non-oscillatory, depolarising, slowly desensitising, and rapidly deactivating Ca^2+^ currents, leading to initiation of firing in quiescent cells, an increase in the frequency of action potentials in spontaneously active cells, and a transient stimulation of LH release. The ATP signalling is contextual, because inositol-1,4,5-triphosphate-dependent oscillations were found to be facilitated, slowed, or stopped, depending on ATP concentration.

The available data clearly suggest a putative autocrine role for ATP in gonadotroph function, although it cannot be excluded that ATP also acts in a paracrine way on other gonadotrophs in the neighbourhood. The physiological conditions under which ATP action takes place also remains to be studied.

## Corticotrophs as autocrine/paracrine cells and targets

Corticotrophs transduce the stress response registered in the brain towards peripheral organs. They do this directly by releasing ACTH in response to CRH and vasopressin but also by transmitting this response to other hormonal cell types in the pituitary. Again, tuning of the corticotrophs via autocrine loops can be anticipated due to the complexity and context of the stress phenomenon (Fig. 3).

### Vasopressin and paracrine communication among corticotrophs

It is well known that arginine-vasopressin (AVP) is one of the corticotroph secretagogues. It releases ACTH via the V1b-R (564). In addition, AVP enhances the responsiveness of corticotrophs to CRH in terms of ACTH secretion (565) but not in terms of POMC gene transcription (566). AVP plays an important role in chronic stress (564) and appears to compensate for the lack of CRH drive on ACTH secretion in CRH-R1 knockout mice (567). The main source of AVP is the hypothalamic paraventricular nucleus but the anterior pituitary itself contains AVP (568–570) and pro-AVP mRNA (569). Pro-AVP mRNA is located mainly in corticotrophs, although AVP immunoreactivity has been detected in all hormone-secreting cell types except somatotrophs (571). Approximately 45% of the anterior pituitary cells are AVP-immunoreactive (569). AVP is secreted by cultured pituitary cells but this is not augmented by CRH (569) and remains elevated during exposure to glucocorticoids, which is consistent with its role in chronic stress situations. Glucocorticoids even increase the coupling efficiency and signal transduction of the V1b receptor (564).

To what extent pituitary AVP contributes to the output of pituitary ACTH under basal conditions or during enhanced input of CRH and AVP from the hypothalamus, however, remains unexplored. This is surprising because highly selective antagonists of the different AVP receptors are available that would allow experiments to demonstrate an autocrine action of endogenous AVP.

Nevertheless, there are data supporting inhibitory and stimulatory paracrine control of corticotrophs amongst each other in relation to the actions of AVP in the pituitary. Not all corticotrophs are responsive to CRH or AVP and a remarkable observation is that AVP augments the number of corticotrophs that are responsive to CRH. CRH also increases the percentage of corticotrophs that bind AVP (572–574). The latter regulation is modulated by paracrine interactions.

#### Inhibitory paracrinicity

Schwartz and Cherny (574) have demonstrated a peculiar auto-control system among corticotrophs in the rat that regulates the proportion of AVP- and CRH-responsive corticotrophs, and this may obviously modulate the overall responsiveness of the pituitary HPA axis at the pituitary level. The authors showed that elimination in culture of CRH target cells, by treatment with a CRH-toxin conjugate (taken up by receptor-mediated endocytosis, the toxin being released intracellularly), did not result in a fall of basal ACTH secretion, which was even elevated, despite the number of corticotrophs and overall ACTH content being decreased by the treatment, suggesting that CRH-responsive corticotrophs inhibit the secretion of the other corticotrophs. A similar observation was later made using sheep pituitary cells (575). This inhibitory paracrine mechanism was confirmed by means of the reverse haemolytic plaque assay (576). By comparing the ACTH response of cells seeded at different densities, it was observed that the number of CRH-responsive corticotrophs increased to almost double when a certain distance between the cells was exceeded, presumably because the paracrine factor depressing responsiveness dilutes out and becomes ineffective when distances between cell become too large. That factor appears to be delivered by the CRH-responsive cells themselves because selective laser-ablation of the CRH-responsive cells allowed ACTH secretion in response to CRH by cells that before were not secreting ACTH. The latter mechanism was proposed to have a role in holding corticotrophs in reserve.

#### Stimulatory paracrinicity

There is also evidence for the existence of a stimulatory paracrine factor released from non-CRH-responsive cells, as medium conditioned by exposure to a pituitary cell population, in which CRH-target cells were destroyed by a CRH-toxin conjugate, was found to increase ACTH secretion in naive pituitary cells (574).

AVP also appears to be implicated in the local control of the total size of the corticotroph cell population (577, 578). This population expands after adrenalectomy and chronic stress and involutes by glucocorticoid treatment. Adrenalectomy also increases cell mitosis in the pituitary but the bulk of mitotic cells do not express ACTH. They may be progenitor cells or stem cells. AVP appears to mediate the effect of adrenalectomy because, in V1b receptor null mice and in mice treated with a V1b antagonist, this mitotic response was absent (579). V1b-R gene knockout also prevents the increment of corticotroph number after long-term adrenalectomy. The question remains as to whether this population growth is induced by the risen output of hypothalamic or of pituitary AVP or both. Since destruction of the AVP neurones in the hypothalamus did not affect basal ACTH levels, although it strongly reduced stress-induced ACTH release (580) but was unable to prevent corticotroph proliferation after adrenalectomy (581), it is possible that adrenalectomy induces pituitary AVP to support basal ACTH secretion and to mediate a trophic effect on the corticotroph population. In support for the latter hypothesis is the finding that adrenalectomy increases pituitary AVP content (568) and exogenous AVP increases cell proliferation in the anterior pituitary (578, 582, 582). Very interestingly, the same population of progenitor cells that develops after adrenalectomy also proliferates after gonadectomy (578). We also found enhanced cell mitosis in pituitary aggregate cell cultures during the first week of culture (116) and this mitotic activity was for a large part in nonhormonal cells (393). Other investigators found a three-fold increase in pituitary AVP secretion after a 3-day culture period (570). Thus, there may be a relationship between pituitary AVP and progenitor cell mitosis.

### CRH and urocortin peptides

Pecori *et al*. (583) have provided evidence for an autocrine or at least paracrine activity of CRH on corticotrophs in the anterior pituitary. Combined *in situ* RT-PCR and immunocytochemistry demonstrated the presence of CRH in corticotrophs and CRH was found in the medium of anterior pituitary cell cultures. Incubation of anterior pituitary cells with an anti-CRH antibody reduced basal ACTH secretion compared to non-immune serum-treated controls. The antibody as well as α-helical CRH_(9−41)_, a CRH antagonist, also blunted the ACTH response to K^+^ and forskolin.

The recently identified urocortin peptides play an important role in the HPA axis, in part by attenuating various functions activated by CRH. The urocortin gene is highly expressed in the anterior pituitary of the rat and human (584, 585). An autocrine or paracrine action of urocortin is feasible because urocortin was localised in corticotrophs in fetal sheep pituitary and transfection of sheep pituitary cells in culture with urocortin antisense oligonucleotides depressed ACTH secretion, whereas exogenous urocortin stimulated ACTH release (586). In human, the great majority (75%) of urocortin-immunoreactive cells were shown to be somatotrophs, whereas 20% were lactotrophs and only a few were corticotrophs, suggesting that urocortin is a paracrine rather than an autocrine peptide on corticotrophs. Urocortin II is also expressed in the anterior pituitary (587), in the rat more specifically in the corticotrophs, where its expression is increased by CRH and inhibited by glucocorticoids (588). To date, no evidence for the expression of urocortin III in the pituitary has been found (589).

CRH and the different urocortin peptides bind with similar affinity to the CRH1-R, but the affinity of urocortin for the CRH2-R is much higher than that of CRH, whereas urocortin II has no affinity for CRH1-R (590). Since CRH1-R is mainly expressed on corticotrophs and CRH2-R mainly on gonadotrophs (591), it can be expected that urocortins have an autocrine and paracrine function on both the HPA and HPG axis but this needs still to be demonstrated experimentally.

The physiological significance of CRH and urocortins within the anterior pituitary needs to be further investigated but it can be proposed that the presence of CRH and urocortins in the pituitary may explain why POMC mRNA levels are not decreased during hypothalamic-pituitary disconnection (584) and why humans with panhypopituitarism due to agenesis or transsection of the pituitary stalk still have ACTH secretion (592). Division of labour between the hypothalamus and anterior pituitary for providing basal ACTH secretion would make sense for a system that has been of crucial importance for survival and evolution.

An interesting observation is that in lower vertebrates CRH stimulates the release of TSH, and hence of αGSU, via the CRH2-R (593). This phenomenon may pave the way to explore whether urocortins influence the release of αGSU in mammals as well, particularly because cells exist in rat as well as chicken pituitary that express both POMC and αGSU (161).

### Acetylcholine

#### A neurotransmitter in non-neuronal tissue

The anterior pituitary has been one of the first non-neuronal tissues in which a cholinergic system has been identified. Today, it has become clear that extra-neuronal cholinergic systems are present in many tissues (65–71). Anterior pituitary acetylcholine has been established as a paracrine factor by Carmeliet and Denef (65, 66, 260, 594). Choline acetyltransferase (ChAT) has been demonstrated in the cytoplasm of rat pituitary corticotrophs by means of different polyclonal and monoclonal antibodies. Production and release of acetylcholine was demonstrated in cultured rat anterior pituitary cell aggregates using [^3^H]choline as precursor. Acetylcholine synthesis was blocked by classical inhibitors of neuronal acetylcholine production. Both synthesis and release of acetylcholine are increased by glucocorticoids. Also, the corticotroph cell line AtT20 expresses a functional cholinergic system.

#### The pituitary cholinergic system appears to be highly context-dependent

Perifusion of anterior pituitary cell aggregates or organ-cultured anterior pituitaries with the muscarinic agonist carbachol can stimulate or inhibit basal PRL and GH release, depending on the hormonal environment. PRL release is stimulated in the presence of T3 but inhibited in the presence of T3 and glucocorticoid simultaneously; GH release is stimulated in the presence of T3 but inhibited in the presence of glucocorticoid. A paracrine action of endogenous acetylcholine could only be demonstrated for the inhibitory component of acetylcholine on secretion. Indeed, in perifused pituitary aggregates, muscarinic receptor antagonists evoked a dose-dependent (0.1–100 nm) increase in basal PRL and GH secretion only when glucocorticoids had been added to the culture medium. No effect of the antagonists was seen under conditions in which carbachol showed a stimulatory effect on secretion, indicating a stimulatory paracrine action is not active or desensitised under basal conditions. Muscarinic antagonists also potentiated the stimulation of GH release by the β-adrenergic agonist isoproterenol and of PRL release by VIP in glucocorticoid-supplemented aggregates.

As shown in frogs, acetylcholine is a putative autocrine factor in melanotrophs in the intermediate lobe of the pituitary. These cells also express ChAT, and acetylcholine stimulates α-MSH release via an M1-R (595–597). Whether endogenous acetylcholine has a similar action and when it is recruited physiologically, remains to be studied.

#### Cross-talk with the NO system?

There are highly suggestive data that the cholinergic system cross-talks with the NO system (1). FS cells express nNOS (2, 517, 518). Carbachol inhibition of GH release in aggregates can be blocked by the calcium channel blockers cadmium and verapamil (594), consistent with the activation of nNOS by intracellular free calcium (3, 598). FS cells express muscarinic receptors, probably of the M1 type, that mediate activation of phospholipase C and intracellular free Ca^2+^ rises (4, 599). Carbachol does not inhibit GH secretion in the GH3 cell line cultured as aggregates (5, 594). The muscarinic inhibition of PRL release is abrogated by the NO synthase inhibitor L-NAME (6, 600). Muscarinic inhibition of PRL release is more prominent in pituitary cell aggregates from male rats than in those from females (260), which is consistent with the knowledge that, in male rats, nNOS resides mainly in FS cells whereas, in females, it resides mainly in LH cells (7, 518). The inhibition of PRL release by acetylcholine found in aggregates is lost when cells are attached to cytodex beads, which precludes a tight contact with FS cells (260).

#### Putative functions of pituitary acetylcholine

The role of pituitary acetylcholine remains to be identified but several data at least suggest some putative links. The glucocorticoid dependency of paracrine inhibition of GH and PRL release suggests a relationship of the pituitary cholinergic system with the modulation of these hormone secretions during stress. The suggested implication of the FS cell NO system (see above) in the inhibitory response points towards a role of the pituitary cholinergic system during immune or inflammatory stress. The FS cells are important targets for inflammatory molecules in the pituitary and during inflammatory events GH and PRL secretion change (see below). Moreover, the pro-inflammatory molecule IL-1 was found to down-regulate ChAT (601). IL-1 is known to activate the HPA axis and to alter GH and PRL secretion via hypothalamic and pituitary sites of action (see below). The inhibition of acetylcholine production by IL-1 has biological sense as, during immune stress, GH and PRL secretion increase, and inhibiting ChAT would result in less tonic inhibition of these secretions by paracrine acetylcholine.

The pituitary cholinergic system might also be related to the HPG axis. Inhibition of PRL release by acetylcholine is not uniquely dependent on glucocorticoids and can be seen in the combined presence of T3 and oestradiol (600). Here, a possible link may exist with the NO system in gonadotrophs. Moreover, there is ChAT expression also in some lactotrophs (65, 66, 260, 594) and this expression may be affected by oestrogens, although this remains to be demonstrated.

It is also interesting to relate the well-known rise in PRL plasma levels during ageing to the pituitary cholinergic system, since basal and TRH-induced PRL release become less sensitive to inhibition by acetylcholine with age (602).

It is noteworthy that a subpopulation of POMC neurones in the arcuate nucleus of the basal hypothalamus also expresses the elements of a cholinergic system (603, 604). POMC neurones in the arcuate nucleus also coexpress CART and are central regulators of energy homeostasis by suppressing food intake (456). Cholinergic mechanisms are known to interfere in energy homeostasis. Nicotine reduces appetite and body weight (605, 606) and a M3-R-mediated cholinergic pathway operates downstream of the hypothalamic POMC system, facilitating food intake (607). Just like POMC cells in the arcuate nucleus (456), pituitary corticotrophs express CART and a cholinergic system. Thus, it appears that the coexpression pattern in POMC cells of the pituitary and hypothalamus are very similar to each other, suggesting a concerted action of the expressed molecules for a common aim. It would be worthwhile to evaluate whether ACh co-operates with POMC-derived α-MSH and CART to increase energy expenditure via a pituitary-located effect on GH and PRL secretion. GH is catabolic in terms of promoting lipid and carbohydrate breakdown (448). During periods of fasting, lipid utilisation is promoted in part via GH secretion. Fasting increases plasma GH levels in all species although, in the rat, the amplitude of GH pulses is decreased but basal levels may be increased (608, 609). During fasting, pituitary GHRH receptor and ghrelin receptor mRNA level and sensitivity to GHRH and ghrelin is increased (610, 611). Since acetylcholine is inhibitory on GH release in the presence of glucocorticoids (594), it could be assumed that GH release in response to a GHRH pulse will be smaller in the rat when the HPA axis is activated. Since, during fasting, the HPA axis is activated (612), the pituitary cholinergic system may contribute to the decrease in GH pulse height during fasting in the rat.

### Neuromedin U (NMU) and apelin

Several other peptides are located in corticotrophs and are putative autocrine factors, namely NMU) (613, 614) and apelin (615). NMU is anatomically and functionally linked to the HPA axis at several levels. It amplifies the stress response and reduces food intake (616). In the rat, the pituitary is the tissue with highest expression level of NMU mRNA. Significant expression is also found in the pars tuberalis (617). The pituitary does not appear to express the NMU receptor NMU-R1 (618, 619) (which, until recently, was an orphan receptor called FM-3 or GPR66) (620), but low level expression of the NMU-R2 (previously called TGR-1) was found (621, 622). So far, direct effects of NMU on anterior pituitary function have not been reported. However, in obese (fa/fa) Zucker rats, NMU expression in the anterior pituitary (and pars tuberalis) is decreased, whereas fasting in rats lowers the anterior pituitary NMU mRNA level, suggesting a role for NMU in adapting ACTH secretion to lower energy expenditure during fasting (617). Another interesting observation is that anterior pituitary NMU content increases several-fold upon administration of TRH (623), an effect established via increased T3 secretion, suggesting a link between the HPT and HPA axes via NMU in the pituitary.

Apelin, the endogenous ligand of the human orphan GPCR APJ, as well as the apelin receptor, are located in corticotrophs and melanotrophs, although, in part, also in other unidentified cells (624). Since apelin is capable of stimulating basal ACTH secretion (624), it is a putative autocrine ACTH secretagogue but the secretion of apelin still needs to be shown. Interestingly, apelin inhibits cAMP levels (625, 626). The negative coupling to adenynyl cyclase also suggests an inhibitory component in apelin action, which may be unmasked in the presence of CRH.

### Intermedin/adrenomedullin-2, the PRL-releasing factor (PRF) from the neurointermediate lobe (NIL)?

It is known for many years from the work of Nira Ben-Jonathan and Georges Nagy that the NIL produces PRL releasing factors that appear to be important for the suckling-induced PRL release (627). This factor may reach the anterior pituitary lactotrophs, particularly those that are concentrated near the NIL (83), by diffusion or via the small portal vessels. One of these PRFs may be oxytocin (628) and another salsolinol (629), a dopamine-derived compound. There are still other PRFs that can be distinguished from β-endorphin, α-MSH, β-MSH, ACTH, TRH, angiotensin II, VIP and corticotrophin-like intermediate peptide (630).

Recently, a novel peptide belonging to the calcitonin/CGRP/amylin/adrenomedullin family has been discovered. It was named intermedin (IMD), also known as adrenomedullin-2 (631, 632). The peptide is located in both central and peripheral tissues and in the anterior and intermediate lobe of the pituitary (626, 631), more precisely in corticotrophs and melanotrophs, but not in other cell types (631). IMD has a selective PRL-releasing activity in pituitary cells of female (631), but not male rats (633), and inhibits GHRH-stimulated (but not basal) GH secretion and cAMP levels in the latter (633). A physiological role of IMD in lactation is supported by the finding that, during lactation, the expression of IMD doubles. Conversely, ovariectomy causes a 90% reduction of IMD expression in the pituitary whereas oestrogen treatment is stimulatory (631). On the basis of these data, IMD has been proposed to represent the PRF from the NIL (631).

It is important to realise that the specificity in the action of the individual calcitonin-like peptides is dependent on interaction with coreceptors. Calcitonin classically acts through the calcitonin receptor (CR), a GPCR, but the other calcitonin-like peptides act through a heterodimer consisting of the CR or the calcitonin receptor-like receptor (CLR), also a GPCR, and either one of the recently discovered ‘receptor activity-modifying proteins’ (RAMP-1, -2 and −3) (634). RAMPS are one-span transmembrane proteins that enable CLR delivery to the cell surface and determine the selectivity of the heterodimer for the individual calcitonin peptide. Thus, the CR/RAMP-1 and CR/RAMP-3 heterodimer form amylin receptors, the CLR/RAMP-1 heterodimer functions as the CGRP receptor, and both CLR/RAMP-2 and CLR/RAMP-3 are functional adrenomedullin receptors. Heterodimerisation of CLR with any of the three RAMPs is sufficient to generate an IMD receptor (631, 632).

The machinery for assembling selective receptors for the calcitonin peptides exists in the pituitary, and regulation fits with a role of IMD during reproduction. The mRNAs coding for RAMP-1 and -3 are detected in the anterior and intermediate lobes (631) and, during lactation, the expression of RAMP-3 doubles whereas levels of CLR and RAMP-1 do not change (631). Changes in RAMP-3 expression are also seen during the oestrous cycle and pregnancy in other parts of the reproductive axis (274). As discussed in the latter review, in most tissues under basal conditions, expression of RAMP-3 is relatively low and mainly RAMP-2 is expressed. During pregnancy, the expression of RAMP-3 is strongly increased whereas RAMP-2 and CLR expression are depressed. Furthermore, regulatory regions of the RAMP-3 gene contain oestrogen response elements. Increased RAMP-3 expression has been suggested to switch the cell from a state of high responsiveness to adrenomedullin (high RAMP-2 expression under basal conditions) to a blunted responsiveness.

To further assess the putative function of IMD, further work is needed to study the consequence of pharmacological IMD receptor blockade or immunoneutralisation of IMD on suckling-induced PRL release. Most interesting in this respect is the compound BIBN4096BS, a nonpeptide CGRP antagonist that acts at the extracellular interface of the RAMP-1–CLR protein interaction (635), already indicating a proof of principle.

It is also most important to realise that calcitonin, adrenomedullin and CGRP are made by gonadotrophs (see above) and IMD is made by corticotrophs and melanotrophs. In this way, the CLR/RAMP-3 could be an interface integrating signalling between gonadotrophs, corticotroph/melanotrophs and lactotrophs, particularly during pregnancy and lactation, when the entire pituitary function is homeostatically adapted.

## Thyrotrophs as autocrine/paracrine cells and targets

Thyrotrophs have attracted relatively less attention with respect to their role as autocrine/paracrine cells or as paracrine targets. Yet, to meet particular physiological needs in the body, the function of these cells also needs to be co-ordinated with that of other pituitary cell types. For example, during cold stress, the HPA axis is activated and there is a need for increased metabolic rate in order to maintain body temperature. This is brought about by activation of the HPT axis (636). During pregnancy and lactation, energy consumption has to be adapted to the needs of the growing fetus and newborn and, during starvation, energy expenditure needs to be minimised. Under these conditions, the metabolic rate needs to be changed through increased or decreased TSH output. Therefore, it is expected that corticotrophs and gonadotrophs may signal to thyrotrophs. Furthermore, thyrotroph function may be adapted to changing needs through alterations in putative thyrotroph autocrine factors, the synthesis and/or release of which may be affected by peripheral or hypothalamic hormones.

### Neuromedin B (NMB)

This peptide belongs to the bombesin peptide family, in mammals together with gastrin-releasing peptide (neuromedin C) (183). The anterior pituitary appears to be the tissue with the highest concentration of NMB in the body (637). In rat, mouse and human, it is mainly found in thyrotrophs (614). There is ample evidence for an autocrine negative feedback of NMB on thyrotroph activity. In isolated pituitary or cultures, exogenous NMB inhibits TSH secretion whereas an anti-NMB antibody has the opposite effect (638, 639). This can be explained by an autocrine action of endogenous NMB, because thyrotrophs are not numerous and can occur rather isolated in the tissue, so that paracrine actions on other thyrotrophs appear unlikely. NMB also attenuates TRH-stimulated TSH release *in vitro* (640). Consistent with the characteristics of an autocrine system, NMB content in thyrotrophs increases with physiological changes that have a negative impact on TSH secretion, such as fasting and diabetes when TSH secretion is decreased (641). The pituitary content of NMB increases in hyperthyroidism and decreases in hypothyroidism. In hypothyroid pituitaries, thyroxine or T3 increases NMB content within 30 min and this is associated with suppression of TSH secretion (642). Somatostatin treatment, which inhibits TSH secretion, also raises NMB content (643). The NMB autocrine loop is physiologically related to energy homeostasis, as TRH and leptin rapidly decrease pituitary NMB levels, which, in turn, might increase the efficacy of TRH on TSH release (644). Mice with a disrupted NMB gene display slightly enhanced TSH plasma levels, reduced T3 levels, increased TRH receptor mRNA level in the pituitary and an enhanced TSH response to TRH compared to wild-type mice, demonstrating that the autocrine action of NMB may also operate *in vivo* (645).

### Leptin

As already mentioned earlier, leptin is found in various cell types in the anterior pituitary, including thyrotrophs. Leptin decreases pituitary NMB levels (644). These data may point towards an inter-relationship between leptin and NMB in the control of TSH secretion at the pituitary level during adaptation to nutritional status. When fat stores are high, circulating leptin is high and TSH secretion should not be restrained. However, during starvation, when circulating leptin is low, NMB would rise, which, in turn, would result in less TSH release, and this would help saving energy stores. This reasoning is confirmed by *in vivo* observations that, in normal fed rats, serum TSH levels increased and pituitary NMB content decreased 2 h after subcutaneous injection of leptin (644). There is direct evidence for an autocrine or paracrine action of endogenous pituitary leptin (646). In isolated pituitaries from hyperthyroid rats (in which NMB is high), leptin reduced TSH release whereas antileptin antiserum increased TSH secretion. In pituitaries from hypothyroid rats (in which NMB is low), however, there was no effect of leptin nor of leptin antiserum on TSH secretion. These data suggest that the leptin–NMB–TSH axis is affected by thyroid negative feedback.

### AVP

This peptide is known to render more cells responsive to CRH (see previous section). The identity of these target cells has not been established (647) but one candidate is the thyrotroph. It has been shown that almost as many thyrotrophs as corticotrophs bind AVP and part of these thyrotrophs starts expressing POMC rapidly when the animals had been subjected to cold stress *in vivo*, an effect that could be simulated by AVP treatment *in vitro* (572).

## Paracrine control by nonhormonal cells

The nonhormonal cell population of the anterior pituitary represents a substantial proportion of the pituitary cell population. Based on estimates by means of single-cell RT-PCR, we count approximately 30% in 14-day-old rats and approximately 10–20% in adult rats and mice as being nonhormonal (648, 649). Part of the nonhormonal cells may be degranulated hormonal cells in a quiescent phase of their secretory cycle but they remain poorly defined as a population due to lack of a biochemical marker. Their proportional number appears to depend on the hormonal status since reciprocal changes in the number of nonhormonal cells and of GH cells or PRL cells have been observed after treatment with glucocorticoids (increasing the number of GH expressing cells) (Pals and Denef unpublished observations) or T3 (increasing the number of PRL expressing cells) (648). The other nonhormonal cells include FS cells (5–10%), dendritic cells (650, 651), macrophages (650), endothelial cells, pericytes (see below), fibroblasts, transferrin-positive cells (652) and, as recently identified, ‘colony-forming cells’ (653–655), nestin-positive cells (656) and cells displaying the peculiar capacity of exporting the dye Hoechst-33342 (657). The latter cells are called ‘side population’ (SP) cells on the basis of their sorting behaviour in a flow cytometry system, and amount to approximately 2% of the cells in mouse pituitary (657).

The nonhormonal cells are small- to medium-sized and display low granularity. When dispersed and sedimented at unit gravity, they remain in the upper layers of the sedimentation gradient system (656). As examined by flow cytometry, most FS cells do not sort within the SP but between the SP population and the bulk of hormonal cells (657). Thus, with appropriate machinery, it is possible to study subpopulations of nonhormonal cells with respect to their putative paracrine actions.

### ‘Side population’ cells may have local activities in cell renewal

The SP cells include nestin-positive cells (656) and cells expressing stem cell antigen-1 (sca-1) and markers typical for stem cells in other tissues (Oct-4 and nanog) and early embryonic markers such as Lhx4 and molecules belonging to the Notch, Sonic hedgehog and Wnt signalling pathways (657). On the basis of the latter expression profile, SP cells are suspected to include pituitary stem cells or early progenitor cells. However, whether these are stem/progenitor cells of hormonal or nonhormonal cells or both, remains unknown. The stem cell/progenitor cell hypothesis is further supported by the finding that the SP cells spontaneously make proliferating ‘spheres’ when kept in suspension culture (656) and grow rapidly in monolayer culture (656) but not in a three-dimensional cell culture system in which hormonal cells are present (648). On the other hand, stem cell markers and early embryonic markers may be phenotypic features of cells with trophic roles, as is the case in embryonic tissue. According to this view, SP cells/nestin-positive cells may play a role in local homeostasis and tissue remodelling, particularly because their number increases in response to various growth factors, such as leukaemia-inhibitory factor (LIF), FGF-2 and EGF, and in response to activating the Notch pathway (658), and as in aggregate cell culture nestin-positive cells organise in a network with long cellular extensions (648). Nestin-positive cells display a high mobility when plated on collagen-coated plastic (656), suggesting their potential to migrate to particular targets. Nestin-positive cells have their unique phenotype because nestin does not localise in fibronectin-immunoreactive cells (mesenchymal cells) or sporadic cells expressing α-smooth muscle actin (656). However, nestin-positive cells often codistribute with the latter cells, mostly around capillaries (656).

### FS cells are the best characterised among nonhormonal cells

The characterisation of FS cells has been considerably improved since these cells can be identified on the basis of both cellular and functional molecules. Most if not all FS cells express the S-100 protein (82). The functional marker of FS cells is uptake of the fluorescent dipeptide β-Ala-Lys-Nε-AMCA (659). Several FS cell lines have been produced, such as TtT/GF (mouse) (660), Tpit/F1 (mouse) (661), FS/D1h (rat) (95) and PDFS cells (human) (662). Recently, transgenic mice have been generated that express green fluorescent protein under the direction of the S-100 gene promoter (663).

### Micro-anatomical architecture of FS cells suggests a role of FS cells in microcirculation of nutrients, ions and waste products in the pituitary

**Fig. 7:**
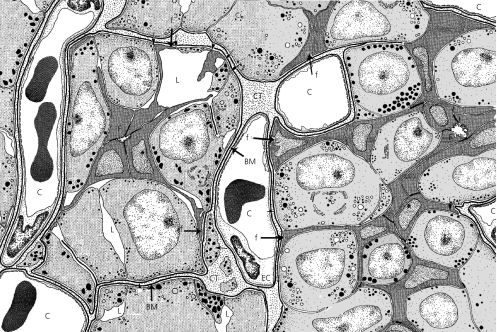
Tissue architecture of the anterior pituitary showing the epithelial cell cords with hormonal cells and folliculo-stellate (FS) cells, the capillaries (C) with fenestrated endothelial cells (EC) and connective tissue (CT). The cell cords are a cluster of endocrine cells surrounding an aggregate of FS cells that make a follicle (F). FS cells also make a meshwork between the hormonal cells, making junctions among each other (thick lines) and extending foot processes (f) ending on the basal membrane (BM) in the periphery of the cord. The cords are surrounded by BM, which may have extensions between some cells. A second BM surrounds the capillary vessels and between these two some connective tissue resides. Small and larger lacunae are present between hormonal cells. Paracrine substances may circulate from cell-to-cell but also could be released in these lacunae and reach more remote places. FS cells make gap junctions mostly among each other, but occasionally also with some hormonal cells. Hormonal cells can make interdigitations with FS cells (small arrows) to favour cell-to-cell communication. Adapted from Vila-Porcile (664).

FS cells form two microanatomical structures (Fig. 7), which may have a large impact on pituitary cell physiology. In the centre of the hormonal cell cord (lobule), they can arrange in clusters and form follicles, usually of submicroscopic size in the rat but in other species, including human, of a larger size (664, 665). In the follicles, numerous microvilli protrude and some cilia are present. Follicle-forming FS cells are polarised. At the apical pole, bordering the follicle, they form tight junctions among each other, although not always fully sealed (666), and, more laterally, junctions of the ‘zonula adhaerens’ type (desmosomes) (664). The basolateral side makes contact with the hormonal cells and with other FS cells, and extends processes that end on the basal membrane surrounding the cell cords (Fig. 7). A second group of FS cells extends long cytoplasmic processes between the hormonal cell types within each glandular cell cord (664). These processes form intercellular junctions, mostly of the zonula adherens-type, amongst each other (667–669), but they are also eletrotonically coupled through gap junctions (665). Some FS cells make intimate foot processes with the basal membrane of the extra-vascular spaces at the periphery of the cell cords (664, 665). In some species FS cells located in the periphery of the cell cords are juxtaposed in a way that they form sinusoid-like spaces (670). Intercellular lacunae are also often seen between hormonal cells (664). Apparently, lacunae between hormonal cells, sinusoid-like spaces surrounded by FS cells and peri-vascular spaces form a microchannel system within the pituitary, through which hormones, paracrine factors, nutrients, ions and waste products can circulate. Such a channel system is thought to play an important physiological role, although the precise details and regulation of flow remain obscure.

Some functional importance can also be inferred from studies on the development of FS cells and follicles. In infant rats (10 days of life) the FS cell follicles are elongated and participating FS cells have a columnar shape without cellular extensions and displaying very little junctions. Later on (30 days), they separate into smaller follicular units and start making extensions and junctions, especially tight junctions (665). It appears therefore that the mico-channel function is more related to the mature pituitary physiology than to the development of the gland. In fact, before postnatal day 10, very little S-100 is expressed in the pituitary, although this expression is induced as soon as the pituitary cells of newborn rats are established in aggregate cell culture (648; Pals and Genef, unpublished observations).

In a series of elegant *in vitro* experiments, using a near-homogenous FS cell population from bovine anterior pituitary or pars tuberalis, Ferrara and colleagues presented strong evidence that FS cells can make tight and functionally polarised epithelia, displaying the typical ion transport characteristics of such epithelia (671, 672). Monolayers of these FS cells, grown on polycarbonate filters and placed in Ussing chambers, show a transepithelial potential difference of approximately 1.1 mV and a short-circuit current, consistent with transepithelial ion transport. These confluent cultures also made domes, a typical feature of cultures of transporting epithelia. The current was inhibited by ameloride applied at the mucosal surface and further depressed by ouabain applied at the serosal surface, indicating a current made by active Na^+^ absorption. Also, the domes collapsed after treatment with ameloride (671). Interestingly, the current was increased by β-adrenergic agonists, prostaglandin E_2_, bradykinin and lysine vasopressin (671, 672).

### Intracellular Ca^2+^ waves from cell-to-cell suggest a role for FS cells in co-ordinating cellular activity

Of utmost functional importance is that FS cells make gap junctions, mostly with the adjacent FS cells (665) but also with a few hormonal cells (673). Junctions among FS cells, however, are incomplete and do not seal off compartments from diffusion of biological molecules; intercellular spaces (between hormonal cells) are also freely accessible for diffusing molecules (668, 669). FS cells are excitable and electrotonically coupled through their gap junctions, as shown by rapid transduction of Ca^2+^ currents over long distances in the gland (674). On this basis, they are thought to co-ordinate activity of hormonal cells. Such co-ordinated activity has been demonstrated at least for somatotrophs. Large clusters of GH cells, visualised by labelling them transgenically with GFP driven by the GH promoter, can display simultaneous intracellular Ca^2+^ transients (675, 676).

Consistent with a function of FS cells in co-ordination of glandular cells is the observation in the mink, that the expression of the gap junctional protein connexin-43 increases in parallel with increased activity of the PRL cells in the breeding season (677). Also in the rat, there is an obvious correlation between the number of gap junctions and reproductive maturation (665). In the immature pituitary, gap junctions are poorly developed, but there is a steep rise at puberty. During the oestrous cycle, their number is lowest at dioestrous. There is an increase at the end of pregnancy and during lactation. GnRH and testosterone markedly increase the number of gap junctions.

The role of follicles remains obscure but the structures are thought to be involved in intercellular transport of metabolic products and ions (82). FS cells also may have a role in phagocytosis as microscopic images showing phagocytised cell debris are more conspicuous when certain hormonal cells regress after a period of hyperplasia (82).

### FS cells mediate and modulate the neuroendocrine response to immune stress and inflammation (Fig. 8)

#### The anterior pituitary drives the response to inflammatory stress

It has been well established that during immune and inflammatory stress, such as induced by bacterial endotoxin (lipopolysaccharide; LPS), secretion of ACTH (678) and of GH and PRL (679) is enhanced whereas pulsatile release of LH (680) and TSH (681) is inhibited. Although this response is initiated in hypophysiotrophic areas in the hypothalamus, which in turn affects pituitary hormone release via the hypothalamic-releasing/inhibiting hormones (682), actions of LPS also occur directly at the pituitary level. Deafferentation of the hypothalamus or surgical removal of the medial hypothalamus does not abolish the activation of the HPA axis by endotoxin (683, 684). Activation of the HPA axis by signals from immune-activated lymphocytes was suggested by Besedovsky in 1981 (59, 685), who observed that cultured immune cells, stimulated with mitogens or antigens *in vitro*, released substance(s) in the supernatant capable of eliciting an adrenal response after intraperitoneal injection. At that time the factor(s) was called ‘glucocorticoid-increasing factor’. Several more recent observations support the essential role of the pituitary itself in driving, at least in part, its response to inflammation. After abolishing the hypothalamic drive of the stress response by transgenic elimination of the CRH-R1 in mice, the activation of HPA axis by local inflammation with turpentine remained pronounced (567). In the latter mice, basal ACTH secretion and the stress response is mediated by AVP but the rise in ACTH and corticosteroid secretion was preserved even after immunoneutralisation of AVP in the latter CRH-R1-null mice (567). CRH knockout mice also retain the capacity to increase adrenal corticosteroid output in response to local inflammation but this no longer occurs after hypophysectomy (686). Thus, it is clear that a direct effect at the pituitary and/or adrenal level by molecules derived from immune cells or microorganisms is essential.

**Fig. 8:**
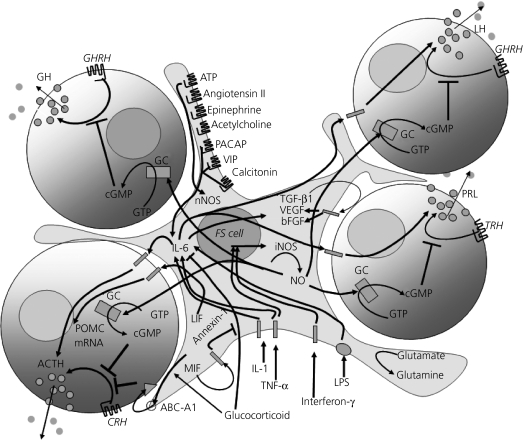
Schematic representation of the paracrine loops thought to act between folliculo-stellate (FS) cells and hormonal cell types and of autocrine loops in FS cells. Interrupted lines indicate hypothetical interactions proposed on the basis of the presence of the indicated factors in the cell and their pharmacological effects on the same cell. →, Stimulatory effect; ⊥, inhibitory effect; ABC-A1, ATP binding cassette A1 transporter; ACTH, adrenocorticotrophic hormone; CRH, corticotrophin-releasing hormone; GC, guanylate cyclase; FGF, fibroblast growth factor; FS, folliculo-stellate; GH, growth hormone; GHRH, growth hormone-releasing hormone; GnRH, gonadotophin-releasing hormone; IL, interleukin; iNOS, inducible nitric oxide synthase; LH, luteinising hormone; LIF, leukaemia-inhibitory factor; LPS, lipopolysaccharide; MIF, migration inhibitory protein; nNOS, neuronal nitric oxide synthase; NO, nitric oxide; PACAP, pituitary adenylate cyclase-activating peptide; POMC, pro-opiomelanocortin; PRL, prolactin; TGF, transforming growth factor; TRH, thyroid-releasing hormone; VEGF, vascular endothelial growth factor; VIP, vasoactive intestinal peptide.

#### FS cells belong to the dendritic cell meshwork of the body

FS cells have characteristics of immune cells. Part of the FS cells expresses markers and functional molecules of monocytes and dendritic cells of the immune system (650). FS cells are now considered to be members of the dendritic cell meshwork throughout the body, together with Langerhans cells in the skin and lymphatic system, ‘veile’ cells, and lymphodendritic and interdigitating cells in a number of tissues (687). All these cells are antigen presenting cells and most of them express S-100, CD1, CD45, CD54, F418, MHC class I and II antigens, Fc and complement receptors (687).

#### FS cells are targets for inflammatory molecules

A potent stimulus for production of the pro-inflammatory cytokines, IL-1, IL-6 and TNF-α by stimulated peripheral immune cells is LPS (688). All these cytokines, including LPS itself, target FS cells (95, 689). FS cells express CD14, which binds the LPS/LPS-binding protein complex and Toll-like receptor type 4, which transduces the LPS signal (689). LPS is known to rapidly activate transcriptional activity of NF-κB, a transcription factor that regulates the expression of many pro-inflammatory cytokines, and this occurs in cells scattered throughout the anterior pituitary (690). Through this action LPS induces expression of IL-1β, IL-1α converting enzyme, IL-1 receptor antagonist and TNF-α (690, 691).

In pituitary monolayer cell cultures, IL-1 stimulates the release of ACTH, LH, GH, and TSH, whereas it inhibits PRL release at concentrations within the range reported for IL-1 in serum (692, 693). However, except for ACTH and GH, the latter response is not a replicate of what happens when LPS is administered *in vivo*. Thus, there is an interplay between hypothalamic and pituitary responses.

#### The anterior pituitary produces cytokines

Various pro- and anti-inflammatory cytokines are produced by the anterior pituitary (e.g. by FS cells). The first cytokine to be identified in the pituitary was IL-6 (694, 695) and we have shown that FS cells are the main cells producing this cytokine (696, 697). Although IL-6 is released from the local inflammatory site (698) and circulates in plasma, local IL-6 from FS cells is involved in the activation of the HPA axis. Indeed, in CRH knockout mice that are still capable of a partial HPA activation via AVP (567), an IL-6 immunoneutralising antibody abolishes this HPA activation (686, 699). An antibody blocking LPS action at CD14 abolished both LPS-induced IL-6, as well as ACTH secretion in aggregate cultures, and a neutralising anti-IL-6 antibody also blocked LPS-induced ACTH secretion in pituitary cell aggregates (700). Interestingly, the latter effect was not seen in monolayer cultures, indicating that intimate intercellular contact is required as is the case in aggregate cell cultures. IL-6 receptors are expressed in corticotrophs and IL-6 can stimulate ACTH secretion directly at the pituitary level (701). The production and release of IL-6 is stimulated by IL-1 in rat anterior pituitary cells *in vitro* (702) and IL-1 and IL-6 act synergistically in stimulating ACTH secretion *in vivo* (703). Also LPS directly stimulates IL-6 release from FS cells (701). Similar local actions of FS cell cytokines appear to be involved as far as the activation of pituitary POMC gene is concerned during inflammation (704).

The paracrine action of IL-6 observed *in vitro* is relevant for the *in vivo* situation because peripheral administration of a neutralising IL-6 antibody or elimination of the IL-6 gene in transgenic mice results in a significant blunting of the plasma corticosterone response to local inflammation (698). Furthermore, also in mice infected with murine cytomegalovirus, IL-6 is mediating the activation of the HPA axis as shown by IL-6 immunoneutralisation in mice in which the central drive of the HPA axis is abolished by CRH gene deletion (686). Remarkably, in mice deficient in either CRH or IL-6, LPS activates the HPA axis less than in wild-type mice but significantly more than in mice deficient in both CRH and IL-6 (705), suggesting the appearance of compensatory mechanisms during severe depletion of stress mediators.

It should be noted that the action of IL-6 is downstream of IL-1, as both induction of IL-6 and activation of HPA axis are inhibited by transgenic inactivation of type I IL-1 receptor (698). Whether this also is the case for the paracrine action of IL-6 in the pituitary remains to be studied.

IL-10, an important anti-inflammatory cytokine, appears to be a regulator in the activation of the HPA axis during immune stress. IL-10 and its receptor are expressed in the anterior pituitary (706, 707). It stimulates ACTH production *in vivo* and *in vitro* (706, 708). However, IL-10 knockout mice secrete more corticosterone during immune stress than wild-type mice (709), suggesting negative control of the HPA axis by IL-10. In human and murine pituitary, IL-11 and IL-11-R mRNA expression has been demonstrated and, as far as tested in corticotroph AtT-20 cells, IL-11 stimulates ACTH secretion and POMC gene transcription (710). The exact pituitary action of IL-10 and IL-11 and their site of production need to be explored further.

Another important endotoxin-inducible cytokine in FS cells is LIF (711). *In vivo*, LIF enhances POMC expression and ACTH secretion in synergy with CRH. LIF is important in mediating the pituitary response to inflammatory stress as transgenic disruption of the LIF gene weakens ACTH secretion (712). However, although null mutation of either CRH or LIF blunts the LPS-induced HPA activation, mice in which both CRH and LIF genes were deleted show a normal HPA axis response to LPS (713), showing that plasticity in the pituitary can compensate for the lacking factors. The latter animals show very high expression of TNF-α, IL-1β and IL-6 in the pituitary (713).

#### Immune stress also activates the GH and PRL axis and inhibits the HPG and HPT axis

Also, in this respect, FS cells are contributing. IL-6 from FS cells stimulates the release of PRL and gonadotrophins in pituitary cell cultures (714), although other studies found it to stimulate LH but inhibit FSH secretion (701, 715). Bilezikjian and colleagues have shown that LPS enhances expression of follistatin and activin B in the pituitary *in vivo* (716), that FS cells are targets for IL-1 (95) and that IL-1 augments the production of follistatin both *in vivo* and *in vitro*, thereby attenuating FSH output in response to activins (716). Noteworthy, the latter effects were not mimicked by LPS or IL-6 *in vitro*, although they were *in vivo*. After LPS treatment, IL-1 appears in rat thyrotrophs (717). IL-1 inhibits TSH secretion (718).

FS cells also contain and release macrophage migration inhibitory protein (MIF) and MIF release is enhanced by endotoxin and glucocorticoids (719). As far as has been tested in the FS cell line TtT/GF, recombinant MIF did not affect basal IL-6 release but antagonised the inhibition of IL-6 release by glucocorticoids, a pro-inflammatory action typical for MIF on classical immune cells (719). MIF has a synergistic effect on LPS.

#### FS cells express several GPCRs possibly modulating the FS cell responses to immune stress

FS cells express β1 and β2-adrenergic receptors (720), acetylcholine (599), VIP and PACAP receptors (721), angiotensin II receptor-1 (722), adenosine A1 and A2B receptors (723) and the TSH receptor (724). Ligands of some of these receptors are known to modulate the function of immune cells. For example, in macrophages, VIP and PACAP prevent iNOS transcription by inhibiting NF-κB and IFN regulatory factor 1 activation. Thus, these GPCRs may function in a feedback scenario to avoid overactivation of the HPA axis, which would lead to immunosuppression by corticosteroid. In FS cells, VIP, PACAP and agents that stimulate cAMP accumulation increase IL-6 production (701). Also, calcitonin was recently found to induce IL-6 production in FS cells (725), representing a putative feedforward mechanism.

#### Inflammatory stress may also implicate the pituitary tachykinin system (e.g. substance P, neurokinin A and B)

LPS was reported to decrease neurokinin A concentration in parallel with PRL secretion in the anterior pituitary (726). Since Substance P may have a paracrine PRL-releasing action (727), the decrease of neurokinin A by LPS may be a mechanism to attenuate excessive PRL secretion during immune stress. It remains unknown where the LPS-induced neurokinin A production is located. Since LPS targets FS cells, they are candidates but, to our knowledge, no data are available to support this hypothesis, the main cell types producing tachykinins in the pituitary being somatotrophs and thyrotrophs (728).

It should be noted that the anterior pituitary also shows vagal innervation from nodose ganglion (729), the nerve endings showing expression of Substance P and CGRP and making close contacts with lactotrophs, somatotrophs, corticotrophs and thyrotrophs (730–734), but not with FS cells (735). Whether these nerve fibres are activated by locally released cytokines and whether Substance P and CGRP are involved in modulating these actions remains unexplored. The issue is important because a neural pathways exists in the transmission of inflammatory signals to the brain via vagal afferents activated by locally released IL-1 (736, 737).

#### The actions of cytokines at the pituitary level appear important for the immune system itself

In a recent review (738), it was noted that GH, PRL and thyroid hormones influence the functioning of the immune system but that these hormones are not obligate for primary lymphopoiesis or for B and T cell-mediated immune responses. Pituitary hormones most likely have a protective action during chronic states of inflammation or other forms of natural prolonged stress. Under particular circumstances, one or more of these hormones can stimulate immune cell function. For example, during pregnancy and lactation, which are physiological states of stress due to the many increased metabolic demands, PRL, GH, thyroid hormones and glucocorticoids are elevated. Glucocorticoids are needed to utilise more glucose and to mobilise protein from mother to fetus. However, the immune-suppressive effects of glucocorticoids might be detrimental and it is thought that the rise of PRL output during pregnancy has an adjuvant role in protecting the immune system from a too profound suppression. Also, GH and thyroid hormone are found to be protective during times of stress.

#### Direct feedback from pituitary hormones to FS cells?

In view of the fact that secretion of all pituitary hormones is altered during immune stress, it has been explored whether FS cells express receptors for pituitary hormones. The TtT/GF cell line indeed expresses mRNA of GH-R, TSH-R and ACTH-R, but not of LH-R, FSH-R and PRL-R (739). This would allow ultra-short feedback actions of these hormones on FS cell signalling to the hormonal cell types that are targeted during immune stress, although this requires experimental verification.

### FS cells may exert scavenger functions through generation of NO (Fig. 8)

Prolonged pituitary activation during immune and other stresses should not lead to excess glucocorticoid hormone production, because this would lead to inhibition of reproduction and to immune suppression, which would predispose to infection or even tumour progression. It is therefore a homeostatic imperative that internal negative feedback loops exist to avoid these digressions. FS cells appear to operate as an interface in these homeostatic reactions as they appear to blunt many stimulated activities of hormonal cells. By making a meshwork with junctional complexes, FS cells create functional cell groups and compartments in the pituitary (665, 674) but they do not shield off cell groups by obliterating diffusion of secreted material (668, 669). Scavenging by FS cells is based on functional inhibition of hormonal cells by material released by them. This was demonstrated by experiments in which hormonal cells were mixed with cell populations enriched in FS cells. When a dispersed cell preparation consisting of approximately 65% FS cells was coaggregated with a highly enriched populations of hormone-secreting cells and the coaggregates, after 5 days in culture, were perifused with various secretagogues, the stimulation of PRL release by TRH or angiotensin II, of GH release by GHRH and adrenaline, and of LH release by GnRH, was blunted, compared to aggregates consisting of the hormonal cells only (94). Interestingly, addition of FS cells also weakened the secretory response to inhibitory signals such as dopamine and somatostatin on PRL and GH release, respectively (668). Under what physiological or pathological conditions FS cells are recruited to attenuate excesses in hormone responses remains to be determined, but it is possible that cytokines, liberated during the activation of the HPA, HPG and/or HPT axis during immune stress may be such mediators or that the FS cells may exert a tonic inhibitory tone. In support for this hypothesis are experiments with pituitary cell aggregates treated with IFN-γ and other cytokines. Prolonged exposure of pituitary cell aggregates to IFN-γ resulted in an inhibition in the secretory response of ACTH, GH and PRL to various secretagogues (740). Also TNF-α and IL-6, but not LPS or IL-1, had an inhibitory action on CRH-induced ACTH release. This inhibition was only seen when FS cells were present in a sufficient number (741). The factor mediating this apparent scavenger effect is, at least in part, NO (742). nNOS is expressed in FS cells and iNOS can be induced in FS cells (but also in some non-identified nonhormonal cells) by IFN-γ (743).

Also, TNF-α may recruit FS cells to blunt other hormonal responses during immune stress. TNF-α is one of the first molecules to appear in blood during inflammation. It is also expressed in the anterior pituitary (744), although the cell type of production remains unknown. TNF-R1 and TNF-R2 mRNA are detectable in the FS cell-derived TtT/GF cell line (745), suggesting that scavenging actions of TNF-α are mediated by FS cells. Although TNF-α was found to stimulate basal ACTH, GH, and TSH, but not PRL secretion in hemipituitaries and dispersed cells upon an acute exposure (746), prolonged (> 4 h) treatment with TNF-α did not affect basal ACTH secretion but depressed CRH- and AVP-stimulated ACTH production and abolished the potentiation of CRH-induced ACTH release by AVP (747). The protein also was ineffective on basal GH and LH secretion, but did inhibit GHRH-stimulated GH and GnRH-stimulated LH release (744, 747). TNF-α-induced inhibition of PRL secretion has been shown to be mediated by NO (748). Of importance, TNF-α augmented GHRH-stimulated GH release in sheep pituitary cell cultures, indicating that species-specific mechanism are to be examined (749).

### FS cells mediate very rapid negative feedback of glucocorticoids via annexin-1 (Fig. 8)

The latter function has been extensively studied by Buckingham and colleagues. Annexin-1 (lipocortin 1) has originally been identified as a glucocorticoid-induced protein that mediates the anti-inflammatory action of the latter hormone in the immune system. Glucocorticoids have a negative feedback on pituitary ACTH secretion and production at the level of the hypothalamus and the pituitary via classic genomic interactions. Glucocorticoids, however, exert also a much faster negative feedback, detectable within 0.5–1.0 h after a rise in plasma glucocorticoid during acute stress and this action appears to be mediated by annexin-1 (750). Annexin-1 is expressed in FS cells and glucocorticoids appear to mobilise annexin-1 to the external cell surface. Addition of annexin-1 to cultured pituitary cells inhibits CRH-stimulated ACTH secretion, whereas the early inhibitory effects of the steroid on secretagogue-stimulated ACTH release *in vitro* and *in vivo* are prevented by blocking synthesis of annexin-1 with antisense oligodeoxynucleotides or by blocking the activity of annexin-1 with anti-annexin-1 antiserum. Interestingly, annexin-1 also appears to inhibit the secretion of PRL, GH, LH and TSH *in vitro* (750). It remains to be investigated whether the scavenging action of FS cells that we have observed previously in coaggregation experiments with enriched FS cells (see earlier in this section) also recruites the annexin-1 system in addition to the NO system. It should be noticed, however, that the latter studies examined long-term presence of FS cells, whereas glucocorticoid-induced annexin-1 actions are examined over a short time interval.

### FS cells may have a role in pituitary plasticity

During postnatal development and during adaptive changes in pituitary hormone secretions, such as during the oestrous cycle, pregnancy and lactation, the pituitary shows remarkable fluctuations in the expansion or involution of certain cell types or functional subtypes. Several growth factors have been identified in FS cells, such as FGF-2 (751), LIF (752), VEGF (44) and, as already mentioned, various cytokines and follistatin. The production of these growth factors is regulated, as indicated by data describing the effect of TGF-β1 and TGF-β3 on VEGF (753) and FGF-2 content (372) and of oestradiol on FGF-2 content (754). Also, PACAP and IL-6 stimulate VEGF secretion whereas glucocorticoid is inhibitory (755). A functional link between FGF-2 from FS cells and lactotroph cell proliferation has already been discussed above, as has the paracrine role of follistatin in relation to the selective regulation of FSH output in gonadotrophs. LIF appears to play an essential role in corticotroph development during fetal life (756).

FS cells also appear to be permissive for the mitogenic effect of oestradiol on lactotrophs and this is far more impressive in rats showing high sensitivity for oestrogen-induced mitogenesis of lactotrophs (Fisher 344 rats) (372, 754). Fisher rats have a higher proportion of FS cells than Sprague-Dawley (SD) rats. Oestradiol itself does not affect the number of FS cells but, when pituitaries from SD rats and Fisher rats are cotransplanted under the kidney capsule or when FS cells derived from Fisher rats are cocultured with either SD or Fisher 344-derived lactotrophs *in vitro*, FS cells from F344 rats increase the mitogenic action of oestradiol, whereas SD-derived FS cells do not (372, 754). FS cells exert their permissive action on oestradiol via FGF-2, the production and secretion of which (but not the action itself) is enhanced by oestradiol to a higher extent in Fisher 344-derived FS cells than in SD-derived FS cells (372, 754). However, the lactotroph's growth response to FGF-2 was similar in both strains. Interestingly, in Fisher rats, but not SD rats, pituitary FS cells show morphological signs of strong hyperactivity when treated with oestrogen (they frequently contained phagosomes including parts of secretory cells, mostly somatotrophs and lactotrophs) (757).

After gonadectomy, FS cells become more numerous and extend cytoplasmic processes to gonadotrophs but, in thyroidectomised rats, this was not observed for thyrotrophs (758), suggesting a particular relationship between gonadotrophs and FS cells. That association is perhaps related to the production of follistatin by FS cells that is well known to attenuate the autocrine action of activin on FSH production. Of note, GH and PRL producing adenomas frequently contain significant numbers of FS cells (759). Furthermore, during lactation FS cells become hypertrophic, displaying an abundant cytoplasm, enlarged Golgi complex, and dilation of the follicles (760). In mice with a genetically induced copper deficiency, the GH axis is dysfunctional and this appears to be due in part to an excessive phagocytic activity of FS cells (761).

Taken together, all these data support a trophic action of FS cells during phases of cell population adaptation to endocrine needs.

### FS cells may be important for glutamate and GABA signalling (Fig. 9)

Rat FS cells have been found to express glutamine synthase, a key enzyme for glutamate metabolism in the central and peripheral nervous system (762). As in other systems, the enzyme is glucocorticoid-dependent (762). As assessed by immunostaining, FS cells also contain high levels of glutamine (763). Expression of glutamine synthase is dramatically age-dependent. Whereas, at 30 days of age, only a small portion of the FS cells is positive for the enzyme, 25% are positive at 60 days and 74% are expressing at 2 years. In the brain, the role of glutamine synthase is to convert glutamate + NH_3_ into glutamine, and is thought to be a protective mechanism against excessive excitation by the neurotransmitter glutamate (764). Glutamate appears to be an intercellular messenger in the pituitary as well. Glutamate or certain aspartate analogues have been found to stimulate PRL, GH and LH release and rat anterior pituitary cells express mRNA of glutamate receptors GluR1, GluR2, GluR3, GluR4, GluR5, GluR6, GluR7, KA1 and KA2 subunits (765). It is therefore feasible that FS cells have a role in preserving optimal levels of glutamate in the microenvironment as is the case for glial cells in the brain. In brain and liver, glutamine synthase is also important for detoxification of ammonium (766).

**Fig. 9:**
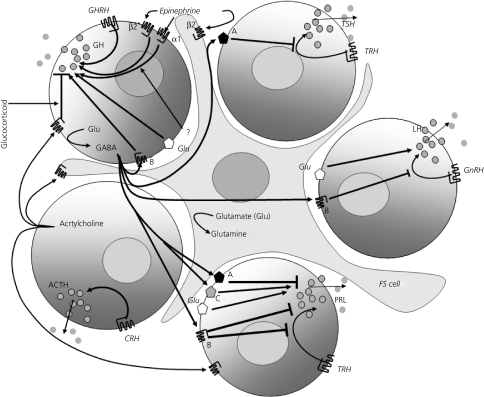
Schematic representation of cholinergic and GABA-ergic paracrine loops thought to act between hormonal cell types and their relationship with nonhormonal cells and adrenergic signals. →, Stimulatory effect; ⊥, inhibitory effect; A, B, C, GABA_A_, GABA_B_ and GABA_C_ receptor subtypes, respectively; 2, 2-adrenergic receptor; 1, 1-adrenergic receptor; ?, unknown factor from unknown small cells, that potentiates the growth hormone response to epinephrine. ACTH, adrenocorticotrophic hormone; CRH, corticotrophin-releasing hormone; FS, folliculo-stellate; GH, growth hormone; GHRH, growth hormone-releasing hormone; GnRH, gonadotophin-releasing hormone; LH, luteinising hormone; PRL, prolactin; TRH, thyroid-releasing hormone; TSH, thyroid-stimulating hormone.

Putative control of glutamate availability by FS cells may also have consequences for the availability of GABA in the pituitary. GABA is delivered to the pituitary via nerve endings (intermediate lobe) and portal blood, but GABA is also synthesised in rat and human somatotrophs by the enzyme glutamate decarboxylase that uses glutamate as substrate (767, 768). Human, rat and monkey somatotrophs express the GABA transporter and GABA_A_, GABA_B_ and GABA_C_ receptors are present in the anterior pituitary (769–771). GABA stimulates GH production via a GABA_B_ receptor, whereas blocking GABA_B_ receptors with phaclofen decreased GH levels in pituitary cell cultures, demonstrating endogenous autocrine or paracrine GABA-ergic modulation of GH production (770). On the other hand, GABA inhibits PRL secretion via GABA_A_ receptor but stimulates it via a GABA_C_ receptor (768). Moreover, endogenous GABA exerts both an excitatory and an inhibitory tone on basal PRL release as shown by the finding that the GABA_C_ receptor antagonist, (1,2,5,6-tetrahydropyridin-4-yl) methylphosphinic acid, and the GABA_A_ receptor antagonist, bicuculline, suppressed and enhanced basal PRL secretion, respectively (768). The GABA_B_ agonist baclofen also inhibited basal and TRH-stimulated PRL secretion in anterior pituitary cells from pro-oestrous rats. Baclofen caused inhibition of GnRH-stimulated LH release in anterior pituitary cell cultures from immature rats (772). GABA also inhibits TRH-stimulated TSH release, probably via the GABA_A_ receptor (773). The importance of GABA_B_ receptors in the HPG and PRL axis is clearly shown in the GABA_B_ receptor-1 knockout mouse (774).

On the basis of all these data, FS cells likely have a local role in glutamate and GABA homeostasis and the latter transmitters exert delicate and balanced paracrine/autocrine effects on GH, PRL and LH secretion, possibly related to basal and immune-stress homeostatic mechanisms.

### FS cells may also be linked to cholinergic and adrenergic signalling in the anterior pituitary

FS cells express muscarinic and β-adrenergic receptors. As already mentioned, acetylcholine is a paracrine factor stimulating GH and PRL release but it becomes inhibitory in the presence of glucocorticoids, an effect possibly mediated through a paracrine inhibitory action of NO released from FS cells by acetylcholine. On the other hand, we have shown that adrenaline stimulates GH release in pituitary cell aggregates through β2- and α1-adrenergic receptors and that this effect is dependent on glucocorticoids (775, 776). Interestingly, the action of glucocorticoids is not a direct one but is mediated by small cells of low density, as demonstrated by coculture experiments of enriched somatotrophs with low density cells obtained after separation by sedimentation at unit gravity (775). The latter cells are probably not gonadotrophs, corticotrophs or thyrotrophs but may be immature lactotrophs or nonhormonal cells or even a subpopulation FS cells that expresses β2-adrenergic receptors. It is tempting to consider that FS cells integrate a network of glutamate, GABA, acetylcholine and adrenergic signals in the anterior pituitary.

### Can FS cells signal through agmatine?

As already discussed, FS cells express nNOS and iNOS, enzymes converting arginine into NO. In neurones and glia, arginine can also be converted to the diamine agmatine by mitochondrial arginine decarboxylase and this substance fulfills most criteria to be a neurotransmitter/neuromodulator (777). Agmatine can be released into the synaptic space, where it can block several ligand-gated ion channels (including NMDA, nicotinic acetylcholine or 5-HT_3_ receptors, all expressed in the anterior pituitary), or bind to either I1 imidazoline binding sites or α2-adrenoceptors. Agmatine inhibits all isoforms of NOS by inhibiting catalytic activity, and reduces expression of iNOS in astrocytes (777). The agmatine system is widely but unevenly distributed among tissues and tissue cell types (778). Agmatine is present at a high level in the hypothalamus (779) and has recently been detected in the anterior pituitary as well (780). Since FS cells already have the machinery to use arginine for NO signalling, it would be worthwhile to investigate whether FS cells are also the site for agmatine formation in the anterior pituitary and whether agmatine has effects on hormone secretion or cellular differentiation, either directly or via its effects on the NO system.

### FS cells can generate retinoic acid

Retinoic acid (RA) plays a critical role in stem cell differentiation and development and is also a regulatory factor in the anterior pituitary. The expression of the RA receptor isoform RXRγ is developmentally regulated in the pituitary and RA is known to activate the GH gene through a RA-responsive element in the GH promoter and in the Pit-1 promoter (781, 782) but to repress the THSβ gene (783). Moreover, RA converts somatotroph progenitor cells into GH-producing cells *in vitro* (784). RA is synthesised from retinoids through retinaldehyde dehydrogenases (RALDH). Recently, it was reported that RALDH2 and RALDH3 are highly expressed in the embryonic rat anterior pituitary (785). Expression was seen already at E12.5, showed peak values at E15.5–17.5 and declined steeply thereafter to disappear after postnatal day 5. The NIL only expresses RALDH3 and no expression was found in the pars tuberalis (αGSU-expressing cells) (785). In the adult gland, RALDH1 is the expressed isoform (786). By *in situ* hybridisation, RALDH1 mRNA was localised in a subpopulation of lactotrophs, in FS cells and in some marginal cells of the cleft. The cell type localisation in the embryonic pituitary was not analysed but since expression was already found at E12.5 when no hormones are expressed yet and *in situ* hybridisation signals were punctuate, it has been proposed that RA is generated in stem/progenitor cells. Taken together, RA from FS cells may be involved in the development of cell lineages in the anterior pituitary.

### Are FS cells related to stem cells?

An old proposal that FS cells are stem cells in the anterior pituitary has still been supported by some investigators on the basis of indirect evidence. Some information discussed in the previous sections, when taken together, may prompt to reexamine that hypothesis. First, FS cells have many characteristics in common with marginal cells facing the pituitary cleft (664, 665). Second, marginal cells are remnant cells of Rathke's pouch, in which proliferation of progenitor cells occurs during embryonic development (787). Third, most recently, expression of retinoic acid forming enzymes has been identified in embryonic rat pituitary progenitor cells and in adult FS cells but also in some marginal cells (785, 786); retinoic acid is important in differentiation of GH progenitor cells (784). Fourth, the first FS cells seem to develop in the postero-lateral region near the marginal layer of the cleft (665). Fifth, cells ultrastructurally identified as FS cells in 10-day-old rats are junction-poor columnar cells making elongated follicles (intragladular extensions of the cleft?) that gradually convert into the normal adult cellular architecture around 40 days of life (665). Sixth, the adult pituitary contains a small and heterogeneous population of nonhormonal cells (side-population cells), not expressing S-100 but showing morphological features (stellate shape, agranular) common to FS cells, which express several genes typical for stem/progenitor cells in other tissues (656, 657); some of these cells also occur in the marginal zone of the cleft.

As already said above, a scenario uniting all proposed concepts concerning the nature of ‘stellate’ cells in the pituitary, is that there is a cell pool in the pituitary that remains primitive and of which part can develop into hormonal cells during postnatal development and part into FS cells. The remainder of these cells may play primarily a trophic paracrine role in basal tissue homeostasis, a role that could be extended to that of being effective progenitor cells when the needs for hormonal output rise to a level that cannot be met by increased cellular activity and cell proliferation alone. Whether such needs exist under normal physiological conditions or whether they arise only under pathological pressure, such as after gonadectomy, adrenalectomy, oestrogen treatment or during tumour development, remains to be seen.

### What do FS cell lines teach us about FS cells?

Because of the extreme heterogeneity of the nonhormonal cell population in the anterior pituitary, significant efforts have been devoted to generate FS cell lines. These are now available, such as TtT/GF (mouse) (660), Tpit/F1 (mouse) (661), FS/D1 h (rat) (95) and PDFS (human) (662). Similar to authentic FS cells, the TtT/GF cells contain many lysosomes and intermediate filaments in the cytoplasm, display phagocytic activity, form follicles, and express GFAP and S-100 (660). The cell line also expresses TGF-β1, TGF-β receptor, IL-6, leptin, leptin receptor, PACAP and PACAP receptors (788). TtT/GF cells express the same regulatory molecules as normal FS cells, obtained by laser-capture microdissection (788). Like normal FS cells, Tpit/F1 cells express nNOS and FGF-2 and respond to PACAP (blunting of IL-6 secretion) and glucocorticoids stimulate glutamine synthase expression (661). Interestingly, ATP stimulates nNOS expression in the FS cell line via P2Y2-purinoceptors (661). Since ATP is cosecreted with hormones, this cotransmitter may provide a paracrine feedback from hormonal cells, activated during immune stress, on FS cells, that, in turn, will dampen the activity of hormone-secreting cells via NO (661).

FS cell lines have so far been studied in monolayer cultures and data obtained in the latter *in vitro* condition may predict what normal FS cells do in monolayer culture. It should be reminded, however, that FS cells in monolayer culture rapidly proliferate (671, 672, 789), while *in vivo* or in a three-dimensional culture system they do not. Thus, the monolayer configuration is artefactual for FS cells. Moreover, it has been observed that, in monolayer cultures, various biological responses in which FS cells might be involved do not occur or are opposite of what is found in reaggregate cell cultures. In the latter, dispersed cells reassociate into a three-dimensional tissue-like structure with a typical FS cell distribution in a meshwork (668). It has been observed that ciliary neurotropic factor and IL-11 have no effect on PRL and GH secretion in monolayer cell cultures, but stimulate these secretions in aggregate cell culture (790). The stimulation of ACTH secretion by LPS is also dependent on a compact cellular organisation. Although LPS stimulates IL-6 secretion in monolayer as well as in mouse pituitary cell aggregates, LPS can stimulate ACTH secretion only in aggregates (700). Another example of differential responses in aggregate or whole pituitary was made in studies on the effect of PACAP on PRL release. In monolayers or in a reverse haemolytic plaque assay system, PACAP inhibits PRL release and has no effect on GH release but PACAP stimulates both GH and PRL release in pituitary tissue blocks and in aggregates as well as *in vivo* in rats with hypothalamic lesions (in which hypothalamic-releasing hormone influence is abolished) (207). Since FS cells organise in an extensive network *in vivo* and have intimate associations with glandular cells, and since FS cells have been shown to co-ordinate cellular activities throughout the pituitary, it is clear that such associations are much better reformed *in vitro* when the cells are allowed to associate in a three-dimensional space. The above experiments therefore clearly suggest that cytokine actions during immune stress can be strongly affected by intimate cell–cell contact between FS cells among each other and with the hormonal cells. Thus, in designing studies on paracrine and autocrine communication in *in vitro* models, the three-dimensional configuration of the tested cells is of utmost importance.

## Paracrine control by connective tissue cells, endothelial cells and pericytes

As is the case for all other tissues, the pituitary contains connective tissue cells and a vascular system. As in other endocrine organs, the vascular system is richly developed. Vessels consist of endothelial cells embedded in the basal membrane and associated with each other by tight junctions. In addition, there are mural cells anatomically and functionally associated with the endothelial cell/vascular tube layer, called pericytes. Pericytes display long cytoplasmic processes embracing the endothelial tube, an ideal position for paracrine signalling (791). In addition, larger vessels are surrounded by smooth muscle cells. The pericyte has a phenotype between vascular smooth muscle cells and fibroblasts with the capacity to differentiate into a myofibroblast.

### Peculiarities of anterior pituitary vessels

The portal vessels of the pituitary lack smooth muscle fibres, but are associated with many pericytes showing highly ramifying processes. Importantly, as in all endocrine organs, the endothelium has many fenestrations and channels, ensuring high permeability for molecules traveling from the interstitial fluid to the blood and vice versa (792). Thus, portal vessels are capillary sinusoids with pericytes rather than typical portal veins. Another peculiarity of pituitary vessels is the lack of von Willebrand factor. We detected cells expressing CD31, a general marker of endothelial cells, in the anterior pituitary of the adult rat, but we found no cells positive for von Willebrand factor, consistent with the sinusoidal nature of the pituitary portal vessels (656). Von Willebrand factor is prominent in veins, but largely absent from sinusoidal endothelial cells (793).

### Plasticity in the anterior pituitary requires tight control of angiogenesis

Once developed, the vascular system is quiescent but new vessels can be made from existing ones by sprouting or intussusception, either during certain physiological conditions where a higher demand for blood supply is required or in pathological states such as during tissue repair after injury or inflammation and tumourigenesis. Angiogenesis starts with basement membrane degradation by activated endothelial cells, then the latter migrate and proliferate, which leads to the formation of endothelial cell sprouts. Subsequently, vascular loops and capillary tubes are formed with tight junctions and, finally, there is new basement membrane deposition (794).

In the pituitary gland, which is highly protected from injury due to its location in the skull underneath the brain, neovascularisation for tissue repair is probably of less importance. However, the pituitary is a plastic tissue capable of remodelling blood supply according to needs, such as during pregnancy and lactation when the gland expands considerably. Thus, tight control of angiogenesis is essential in the adult pituitary. Moreover, the pituitary is prone to develop adenomas and, hence, angiogenesis may be most relevant to their progression.

An important new mechanism recently advanced is that angiogenesis and tissue growth go hand in hand. It appears that neuronal guidance can be mediated by similar factors as vessel guidance (795). Thus, growth factors that are involved in angiogenesis in the pituitary may be relevant to pituitary plasticity as well.

### Pro- and anti-angiogenic factors in the anterior pituitary

In general, angiogenesis depends on the balance of pro-angiogenic and anti-angiogenic growth factors, which are produced by endothelial and tissue cord cells, as well as on remodelling of the extracellular matrix (ECM) to allow endothelial migration. The most important pro-angiogenic factors are VEGF (and different VEGF-like molecules), angiopoietins, FGFs, TGF-α, proliferin, platelet-derived growth factor, IL-8, TGF-β1, and placenta growth factor (796, 797). Anti-angiogenic factors are thrombospondin-1 (a matrix glycoprotein), angiostatin (a cleaved product of plasminogen), endostatin (a cleaved part of collagen XVIII), cleaved PRL and GH fragments, and cleaved perlecan (796). Some angiogenesis inhibitors are intrinsic to endothelial cells such as soluble VEGFR-1, vascular endothelial growth inhibitor (VEGI) and vasoinhibins (798). In addition, many other substances have been found to exert angiogenic (erythropoietin, angiotensin II, ETs, adrenomedullin, proadrenomedullin N-terminal 20 peptide, urotensin II, leptin, adiponectin, resistin, NPY, VIP, PACAP, and Substance P) and anti-angiogenic (somatostatin, natriuretic peptides and neurokinin B) activity (799, 800).

Many of the known angiogenic and anti-angiogenic substances have been localised in the anterior pituitary, but their actual effectiveness on vascularity and permeability in the gland has only been documented for part of them.

#### FGF

Historically, FGF-1 and FGF-2 were the first characterised angiogenic factors, but among the more than 20 members of the FGF family, several other FGFs are angiogenic. FGFs are pleiotrophic: they stimulate endothelial cell proliferation and migration, the plasmin–plasminogen activator system, matrix metalloproteinase (MMP) shedding, integrin and cadherin receptor expression, and intercellular gap-junction communication (801). MMPs degrade ECM and release several growth factors sequestered in the ECM. FGFs participate in vessel assembly, sprouting and vessel branching. The peculiarity of FGFs is that they interact with several binding partners, either located on the endothelial cell surface, or in the ECM, or in the extracellular space as freely moving molecules (801). These molecules strongly affect the angiogenic potential of FGFs. Some of the strongest partners are heparan sulfate proteoglycans (HSPGs), without which FGFs are not active (802–804).

The association of FGF-2 with basal membrane in the anterior pituitary has been clearly demonstrated. In the adult rat pituitary, FGF-2 has been located mainly in FS cells and in a subpopulation of marginal cells of the intermediate lobe facing the pituitary cleft (751). However, during embryonic development of the rat, FGF was localised within all cells of the pituitary (805). Interestingly, FGF from gonadotrophs appears to play a significant role. In 15–20-day-old rat fetuses, dense foci of extracellular FGF were observed at sites of capillary penetration, in the vicinity of partially disrupted gonadotrophs (805). Also, in the adult pituitary, disrupted gonadotrophs containing FGF-2 have been detected, particularly in the postero-lateral zone, closely located near the meningeal membranes, which could represent a site for invading vessels (see below).

#### VEGF

This growth factor was originally identified in FS cell-conditioned medium (44). It is a potent endothelial cell mitogen, stimulates endothelial cell migration and increases fenestration of endothelial cells and, in this way, capillary permeability. VEGF is also found in all hormonal cell types of the pituitary, particularly GH and POMC cells, as well as in various cell lines derived from these cell types (806). The VEGF isoforms, VEGF164 and VEGF120, are expressed in the anterior and neural lobes but not in the intermediate lobe (807). The VEGF receptors flt-1, flk-1 and neuropilin-1 are also expressed in the pituitary (806). Flt-1 was detected in endocrine cells, whereas flk-1 and neuropilin-1 were found to be exclusively expressed in endothelial cells (808).

It is remarkable that VEGF is probably not involved in the initial development of the portal blood vessels to the pituitary, since the formation of portal vessels begins at E13.5, which is 2 days earlier that the first appearance of VEGF-A mRNA in the pars tuberalis and the rostral region of the pars distalis (809). The appearance of VEFG mRNA coincides with the penetration of portal vessels into the pars distalis to connect with the secondary capillary plexus there. In the pars tuberalis, VEGF is located in TSH and FS cells whereas, in the pars distalis, VEGF is located initially in ACTH cells and later also in subpopulations of all cell types (809).

Whereas hypoxia is a strong stimulus for VEGF up-regulation in many tissues, in order to cope with hypoxia and ischemia, it remains to be seen whether this stimulus is also operative in the pituitary. The question seems relevant because the tissue is fed by a portal system that is already less saturated with oxygen.

#### Oestrogens

Oestrogens strongly up-regulate VEGF expression in the anterior pituitary (807). VEGF has therefore been studied in relation to neovascularisation in oestrogen-induced pituitary tumourigenesis and in human pituitary adenomas (807, 810). Oestrogen-induced rat pituitary tumours and GH3 pituitary tumour cells express VEGF164 and coreceptor, neuropilin-1. VEGF164 and neuropilin-1 mRNA and protein levels are significantly higher in tumours and in GH3 tumour cell line (807). VEGF and its receptor Flk-1 are expressed at much higher levels than normal in human nonfunctioning pituitary tumours (808). Also, FGF-2 expression is increased by oestrogen, at least in rats susceptible to develop prolactinomas (e.g. Fisher 344 rats) (811). In the latter rats, oestrogen treatment rapidly leads to lactotroph hyperplasia and causes high FGF-2 expression in the cytosol of gonadotrophs, located in the postero-lateral zone near the intermediate lobe, close to the meningeal blood vessels. The postero-lateral zone is known to be home to many gonadotrophs and lactotrophs and is also the area in which FS cells start to develop during postnatal development (665). Oestrogen treatment leads to neovascularisation growing into the anterior pituitary from these meningeal vessels bordering the postero-lateral zone. ECM-associated FGF was also revealed in foci at the postero-lateral edge. These data clearly demonstrate FGF- and VEGF-mediated angiogenesis in oestrogen-induced tumourigenesis in rats predisposed to tumour development.

#### EG-VEGF

An exciting question is whether the pituitary expresses the endocrine gland-derived vascular endothelial growth factors (EG-VEGF or prokineticin), as has recently been discovered in steroid-producing endocrine glands, the brain, gastrointestinal system and even immune cells (812). These peptides have a wide range of functions but stimulate the endothelial cells in various endocrine glands particularly well (813). However, no data are as yet available for substantial pituitary expression. EG-VEGF cooperates with VEGF in the formation of capillary fenestrations, which, as in other endocrine tissues, are well developed in the pituitary (814).

#### Angiopoietins

The anterior pituitary also expresses angiopoietins (Ang) and their receptor Tie2 (815). These peptides act either as agonist (Ang-1 and Ang-4) or antagonist (Ang-3) in vascular expansion and survival. Ang-2 can stimulate or inhibit angiogenesis depending on contexts. Ang-1 promotes endothelial cell survival (protection from apoptosis) through the Akt pathway and stimulates endothelial cell migration, sprouting and tube formation. The cells that produce Ang-1 and Ang-2 in the anterior pituitary are the gonadotrophs, which is in striking contrast with the neural lobe where strong expression is seen in endothelial cells (816). It is remarkable that, in addition to angiopoietins, gonadotrophs produce FGF-2 and VEGF, which may be relevant to the neovascularisation from meningeal vessels into the postero-lateral zone of the anterior pituitary, known to be rich in gonadotrophs and lactotrophs (see earlier in this section).

#### Pituitary tumour transforming gene (PTTG)

PTTG, an oncogene of which the gene product is a cytoplasmic and nuclear protein (806). It is expressed in low level in many tissues but is strongly up-regulated in many tumours, including pituitary adenomas. It displays a powerful angiogenic effect. Important targets of PTTG are FGF-2 and VEGF, which are both up-regulated. PTTG itself is up-regulated by FGF-2 and oestrogen (817).

#### TGF-β1

TGF-β1 is inhibitory or stimulatory on angiogenesis, depending on the receptor types expressed on endothelial cells. TGF-β up-regulates VEGF production in the pituitary (753).

#### Trombospondin-1 (TSP-1)

The anti-angiogenic factor trombospondin-1 (TSP-1) has been detected in anterior pituitary endothelial cells. Levels go down after oestrogen treatment *in vivo* and in purified pituitary endothelial cells in culture (818). TSP-1 depresses proliferation and migration of pituitary-derived endothelial cells in primary cultures. These data suggest that oestrogen-induced tumour growth may be promoted by down-regulation of locally produced anti-angiogenic TSP-1.

#### Cleaved PRL and GH

Endothelial cells have been shown to produce PRL and GH that exert an autocrine/paracrine angiogenic effects, although this remains to be studied in the pituitary. Most interestingly, PRL and GH can be cleaved by various proteases to smaller fragments of 14–17 kDa (819). Recently, still another cleaved derivative of PRL and GH has been discovered that is specifically processed by the metalloprotease bone morphogenetic protein-1 (BMP-1). The latter cleaves PRL between Ala-159 and Asp-160, turning the molecule from an angiogenic into an anti-angiogenic substance (820). The latter PRL fragment is distinct from the cleaved PRL that we previously identified in the pituitary and is mitogenic for gonadotrophs and thyrotrophs (see above).

## Role of basement membrane and ECM

The basement membrane (BM) is a thin sheet consisting of a meshwork of type IV collagen, laminin, nidogen and HSPG, to which epithelial, endothelial cells or stromal cells are attached. BM therefore provides structural support for cells and makes a barrier between different tissue compartments. Collagen type IV confers structural stability, whereas HSPGs cross-link the collagen type IV and laminins. Many isoforms of the different families that compose the BM exist, so that BM can vary substantially from one tissue to another. In addition, there are several minor components, such as osteonectin, fibulins, collagen types VIII, XV and XVIII, and thrombospondin-1 and -2 (821).

In the rat pituitary, laminin, the HSPG perlecan, and type IV collagen. are found inside nonhormonal cells whereas laminin and, to a lesser extent, type IV collagen are found in hormonal cells, suggesting that hormonal cells participate to the elaboration of BM (822). Laminin is detected in gonadotrophs, thyrotrophs and corticotrophs, little is found in lactotrophs but it is absent in somatotrophs, suggesting differential production according to cell type. Laminin is found in Golgi and secretory vesicles, indicating export of the protein. The pituitary expresses high levels of perlecan, particularly in the subendothelial region of sinusoidal vessels (823) and also shows sequestration of FGF-2 in BM (811, 824).

Increasing evidence suggests that BM has also an important functional role in cell physiology, differentiation and homeostasis. BM laminin can signal to cell surface adhesion receptors, such as integrins, which can also function in concert with growth factors (821). The highly glycosylated nature of BM and the heparin-binding feature of HSPGs make BM a high-affinity and high-capacity binding place of growth factors, like VEGF and FGF-2 (821). Laminins are the functionally active components, with different isoforms generating different signals in different tissues (821). In the pituitary, laminin affects PRL and gonadotrophin secretion and collagen IV has been reported to affect the release of prolactin (825).

Assuming that the BM signals to the cord cells, it is clear that regulatory mechanisms must exist that modulate this signalling, particularly when the tissue has to remodel. MMP isoforms play an essential role in remodelling BM. This is particularly prominent in pituitary adenomas in which very high levels of active MMP-2 and MMP-9 and low levels of tissue inhibitor of metalloproteinases-1 have been reported (825). It has also been shown that MMPs can release growth factors sequestered in the ECM, that, in turn, stimulate pituitary cell proliferation and hormone secretion (825). Another relevant proteases is BMP-1 (820). It plays an important role in the deposition of fibrous ECM. Moreover, it processes perlecan to produce a potent anti-angiogenic factor. BMP-1 activates growth factors such as TGF-β1. BMP1 mRNA is one of the most highly elevated transcripts in endothelial cells of tumours.

ECM and MMPs and their inhibitors play also an important role in angiogenesis (826), which again is prominent in pituitary tumours. In aggressive prolactinomas, expression of the polysialylated neural cell adhesion molecule is up-regulated whereas that of the E-cadherin/catenin complex is reduced (826), indicating altered cell adhesion and cell migration. Chronic oestrogen treatment increases gelatinase (pro-MMP-9) levels in the pituitary of tumour-susceptible Fischer 344 rats.

Another important protein expressed in the anterior pituitary with a potential role in modifying paracrinicity by interaction with the ECM is connective tissue growth factor (CTGF) (827). It is a secreted protein with a main function of promoting cell adhesion through an integrin binding domain, the type of interacting integrin being tissue-specific. Importantly, cell surface HSPG is a necessary coreceptor, interacting with the carboxyl-terminal domain. The latter domain also promotes fibroblast proliferation. A von Willebrand factor domain located more N-terminally interacts with TGF-β and in this way assists in presenting TGF-β to the TGF-β type II receptor. Thus, CTGF seems to exert context- and cell-specific effects. As shown by gene deletion studies, the molecule is essential in the development of mesenchymal cell lineages, but it plays also an important role in the adult, where it is expressed in endothelia and the cerebral cortex, consistent with a role in promoting angiogenesis and tissue integrity (828).

## The dynamics and the spatial dimensions of autocrine and paracrine systems

Perhaps the most difficult aspects of autocrine and paracrine systems are the spatial dimension over which autocrine and paracrine factors work and the dynamics of the receptor–ligand interactions. These aspects are very difficult to study as ligand and receptor densities cannot directly be altered by the investigator, since it is the cell itself that determines them. In the case that the ligand of a receptor is a hormone or a drug, traveling to its target via the bloodstream, receptor-ligand dynamics follow Michaelis–Menten kinetics. By contrast, when the ligand is an autocrine substance that is released by the cell in the vicinity of the surface receptors, only the molecules present in a ‘thin secretion layer’ surrounding the cell, are relevant for the dynamics of the receptor interaction, because all other molecules that diffuse further are indefinitely diluted and lost for receptor interaction (829). One has to consider competition between the ligand capture avidity of the receptor and the diffuse rate into the bulk environment to determine the efficacy of signalling, rather than the *K*_D_ of the receptor and ligand concentration. A situation leading to equilibrium between a receptor and its ligand solubilised in the bulk environment, as is the case for pharmacological interactions, is not existing in autocrine interactions, and, hence, the kinetic model used to describe the interaction quantitatively must be different. For the TGF-α–EGF-R autocrine system it has been demonstrated by biocomputing modelling that the dynamics of cellular responses are directly proportional to the ratio of the rate of ligand production and the rate of receptor production (386). Surprisingly, with a ratio of <0.2, no ligand was found free in the medium, but up to 20% of the EGF-Rs were occupied. Thus, an EGF autocrine system functions by immediately capturing the released ligand and hence the ligand does not reach neighbouring cells and is often undetectable in the culture medium. When this ratio was experimentally increased, the ligand could be found in the medium. It was found, however, that various EGF autocrine systems operate at a ratio of ligand : receptor production rates much smaller than 0.2 (388), indicating that EGF-R autocrine systems are regulated primarily through ligand availability and capture and not through regulation of receptor number. The latter is in clear contrast with what usually happens in classical hormonal or pharmacological systems.

Signalling in autocrine systems appears to be strongly influenced by extracellular molecules that bind the released ligand. The TGF-α–EGF-R loop, for example, was found to only transiently stimulate the intracellular transduction pathway when TGF-α was released and allowed to be bound by certain molecules in the microenvironment, whereas the pathway was activated over a prolonged period when EGF-R could recapture the released TGF-α (830). Furthermore, if several ligands compete for binding to the receptor, the type of ligand will be important for the magnitude and duration of the response. For example, heparin-binding EGF (HB-EGF) and TGF-α are both agonists at the EGF-R, but the former and not the latter binds to HSPG present in the extracellular microenvironment; consequently, the dynamics of the same cell through the same EGF-R will be different when stimulated with TGF-α than with HB-EGF, even when both are shed at the same rate.

Another important consequence of the peculiar dynamics of autocrine systems is the fact that neutralisation of the ligand-receptor signalling by the use of extracellular compounds requires unexpectedly high doses of blocking agent of up to eight orders of magnitude greater than the *K*_D_ for ligand binding to the receptors) (831) and that the efficacy of signalling disruption is much higher when compounds are used that bind the receptor than when substances are used that bind the ligand (831, 832). Complete annihilation of the autocrine response is not obtainable until all bulk ligand has been bound by the administered compound. Moreover, annihilation can only occur when the association rate constant of the compound for binding the ligand is much higher than the association rate constant of the autocrine ligand for its cellular receptor (831). The latter observations are of paramount importance for setting-up experiments as well as strategies aimed to target autocrine systems in the treatment of disease.

In a regular experimental set-up, it is very difficult to distinguish between a pure autocrine effect of a signal on a receptor expressed on the same cell and a paracrine effect on similar receptors on neighbouring cells. Neither is it possible to evaluate whether both mechanisms are operating and what the consequences are for cell physiology. As an autocrine agent, a signal has an advantage compared with its action as paracrine signal: it has a spatial proximity advantage (i.e. when examined in a temporal sequence, the autocrine effects occur before the paracrine effects due to a shorter distance between site of release and site of primary action in case of the autocrine situation). In addition, there is a concentration gradient from the autocrine compartment (high concentration) towards the paracrine compartment (low concentration), which is important to consider if exogenous molecules can interfere with this gradient. Recently, a mathematical model was proposed that allowed to distinctions to be made between auto- and paracrine actions by registering the effect of increasing the volume distribution, in which the secreted signal diffuses, on the auto/paracrine action under study (a macrophage cholesterol efflux model in monolayer) (833). It was found that only the paracrine contribution was affected by an increase in distribution volume, presumably because the autocrine action occurs too closely to the cell surface and cannot be affected by increasing the bulk volume surrounding the cells. Thus, the relative importance of autocrine and paracrine mediation depends on the size of the local distribution volume. These considerations have never been made in the pituitary field and may have great repercussions on interpreting data obtained with monolayer cultures versus data obtained with aggregate cell cultures. The above findings also predict that tissue architecture, ECM elements and size of intercellular spaces and lacunas may profoundly affect cell physiology based on autocrine and paracrine mediators.

## Conclusions

When the first hypothalamic-releasing and inhibiting hormones were discovered and their structure identified some 30 years ago, no one realised that the hierarchy of one releasing hormone–one pituitary hormone–one pituitary cell type was an over-simplification of the hypothalamic hypophysiotrophic hormonal system. Although the rigid boundaries between endocrine systems and neural systems were already fading due to the growing impact of neuroendocrinology, it was not realised that the correct release of each hormone is an integrative phenomenon in which a plethora of signals participates. Many hormones from the hypothalamus, endocrine feedback glands, fat tissue, the immune system and metabolic tissues have been discovered to signal to the pituitary. An astonishing discovery was the masses of regulatory signals that are generated within the pituitary itself, influencing pituitary function either in an independent fashion or by modulating the action of other intra- and extra-pituitary signals.

Several autocrine loops have been discovered that operate in a particular cell type. The best studied autocrine systems are: (i) the stimulatory VIP and galanin, and the inhibitory ET and PRL loops in the lactotrophs; (ii) the stimulatory activin and inhibitory inhibin loops in gonadotrophs; (iii) the inhibitory NMB loop in thyrotrophs; and (iv) cytokine loops in FS cells. Ghrelin in somatotrophs, and CRH and urocortin II in corticotrophs may also form stimulatory autocrine loops. Well explored paracrine systems are: (i) the gonadotroph–lactotroph axis, of which the mediators remain incompletely defined but could be in part angiotensin II, PACAP, N-POMC and calcitonin; (ii) the gonadotroph–somatotroph axis, which is particularly well developed in fish; (ii) a cholinergic system in corticotrophs targeting somatotrophs, lactotrophs and FS cells; (iii) a GABA-ergic system in somatotrophs; (iv) several inhibitory systems targeting gonadotrophs (e.g. galanin and PRL from lactotrophs and β-endorphin from corticotrophs); and (v) several cytokines, such as IL-6, LIF, MIF, follistatin and NO, produced in FS cells and targeting several cell types, and annexin-1 from FS cells mediating the rapid negative feedback of glucocorticoid on ACTH secretion. Many other autocrine and paracrine interactions may exist on the basis of the presence and pharmacology of bioactive substances in particular cell types, but these still need to be characterised.

It is clear that the response to these different signals is not simply the sum of them but that there is integration of hypothalamic, peripheral and local signals that can differ highly according to context. Autocrine and paracrine systems associate in networks that are recruited to establish functional changes. One of these networks consists of VIP–galanin–TGF-β1–TGF-β3–bFGF that is activated by oestrogen to establish its action on PRL release and lactotroph expansion. Networking ensures the positive feedforwarding necessary to elicit the required functional activity. Another integrative system is based on EGF-R transactivation by various GPCR-mediated signals. FS cells have their own cytokine network but can also interact with other networks, such as with the paracrine cholinergic and GABA-ergic systems and the endocrine adrenergic system. FS cells also come in interplay with the oestrogen-dependent autocrine systems in lactotrophs. Many examples demonstrate that autocrine and paracrine systems enable context-dependent signalling and biocomputational models have been presented (830). Responses seen in the presence of adrenal steroids can reverse in the absence of the latter, such as the GH response to acetylcholine and angiotensin II and the PRL response to acetylcholine. Other responses depend on the presence of thyroid hormone, such as the PRL response to acetylcholine. Still other responses, such as responses to galanin, VIP, calcitonin, oxytocin and gastrin-releasing peptide, require the presence of oestrogens. The direction of a response (stimulation or inhibition) may depend on the concentration of oestrogen such as the response of lactotrophs to ETs. Certain receptor subtypes mediate an inhibitory response to GABA, whereas others mediate a stimulatory one.

Integration of signalling in the pituitary is also illustrated by the fact that almost all autocrine and paracrine activities are up- or down-regulated during the physiological change that has an impact on the hormone output during that change, either by changing the expression level of the ligand or by changing the expression level of the receptors, or both. Moreover, the same paracrine factor can be expressed in different cell types, but the change in its expression occurs only in the cell type that is relevant for supporting the changing pituitary function. An example is leptin, which is expressed in all cell types except lactotrophs, but the change in expression that occurs at pro-oestrous is only seen in somatotrophs, consistent with the surge in GH release at that time. Also, gender differences in hormone output are often based on gender differences in the activity of the underlying paracrine system, such as is the case of the galanin system in lactotrophs.

The multiplicity of paracrine factors can be viewed as a biological system aimed at preserving stability in a complex integrating tissue such as the pituitary. Maintaining a certain level of basal hormone release, particularly in somatotrophs and lactotrophs, which are both under substantial hypothalamic inhibitory tone, may also profit from underlying paracrine mechanisms. However, the pituitary is also a plastic tissue that needs to adapt to many life-essential changes such as during the reproductive cycle, stress, metabolic needs, day–night rhythm and changes in energy stores and needs. Changes in hormone output are imposed by the hypothalamic and peripheral hormone signals but are executed in the pituitary. A correct response needs a microenvironment that is well stabilised under basal conditions but can adapt and fine-tune activity when necessary. The latter can occur when the system disposes of autocrine loops that together create a positive feedforward mechanism as well as negative feedbacks.

Nevertheless, despite enormous progress, many questions remain open. For example, although AVP is synthesised in corticotrophs and the potentiation of the ACTH response to CRH by AVP has been extensively studied, it is not known whether AVP from the hypothalamus is the player here or whether it is (also) AVP from corticotrophs. The pituitary displays the highest expression level of various putative paracrine/autocrine factors such as NMU and CNP, yet the function of these peptides in the pituitary remains elusive. Certain peptides, such as the bombesin-like peptide gastrin-releasing peptide, do not show up the endogenous action that is predicted by their pharmacology (183). There are many pituitary peptides that show various effects when added to test systems but for which the endogenous action has not been convincingly demonstrated yet, such as neurotensin, prodynorphin-derived peptides, enkephalin, adrenomedullin, CGRP, NPY, natriuretic peptides, leptin, neuropeptide B and W, calcitonin-receptor stimulating peptide, orexins, and adipokines. Also, the role of local classical releasing/inhibiting hormones TRH, GnRH, GHRH and somatostatin made within the pituitary remains to be specified. Peptides for which an autocrine role has been shown may also function as paracrine factors because receptors for these peptides are not only expressed by the cell of origin, but also by other cell types, although whether this also occurs remains unexplored. In gonadotrophs, NPY, CNP, PACAP and leptin are putative autocrine factors but such a role remains to be demonstrated by direct experimental evidence.

The ultimate question is whether paracrinicity can lead to pathology, particularly with respect to pituitary tumourigenesis. To date, there is no evidence for a primary causal role of disturbed paracrinicity in the pathogenesis of pituitary adenoma but there is indirect evidence for a role in the progression of these tumours. Pituitary adenomas may over-express certain growth factors or their receptors such as FGF-2, EGF, TGF-α, EGF-R, Notch-3, FGF-R1, and VEGF (834–838). Other growth factors, known to be antiproliferative, are down-regulated, such as sonic hedgehog (839) and GFG protein, which is encoded by a bFGF antisense gene (840). The clinical observation of paradoxical hormone secretory responses may also be explained by abnormal expression levels of certain peptide receptors in the tumours (834). It therefore appears that pituitary adenomas may be growing faster by both increased growth factor stimulation and the decreased availability of endogenous antiproliferative protection mechanisms.

It is hoped that the story of 30 years of cellular pituitary cross-talk will inspire future approaches for medical treatment by realising that the basis of disease, and of medically altering its course, is made by a multiplicity of agents and not by solitary or protagonist factors alone. Biophilosophically spoken at least, we have learned that cells determine their fate themselves by recruiting many resources in the microenvironment rather than by obeying only gods acting from remote places.
